# Structure-based design of pan-coronavirus inhibitors targeting host cathepsin L and calpain-1

**DOI:** 10.1038/s41392-024-01758-8

**Published:** 2024-03-06

**Authors:** Xiong Xie, Qiaoshuai Lan, Jinyi Zhao, Sulin Zhang, Lu Liu, Yumin Zhang, Wei Xu, Maolin Shao, Jingjing Peng, Shuai Xia, Yan Zhu, Keke Zhang, Xianglei Zhang, Ruxue Zhang, Jian Li, Wenhao Dai, Zhen Ge, Shulei Hu, Changyue Yu, Jiang Wang, Dakota Ma, Mingyue Zheng, Haitao Yang, Gengfu Xiao, Zihe Rao, Lu Lu, Leike Zhang, Fang Bai, Yao Zhao, Shibo Jiang, Hong Liu

**Affiliations:** 1 Drug Discovery and Design Center, State Key Laboratory of Drug Research, CAS Key Laboratory of Receptor Research, Shanghai Institute of Materia Medica, Chinese Academy of Sciences, Shanghai, 201203 China; 2 University of Chinese Academy of Sciences, Beijing, 100049 China; 3 Key Laboratory of Medical Molecular Virology (MOE/NHC/CAMS), School of Basic Medical Sciences, Shanghai Institute of Infectious Disease and Biosecurity, Fudan University, Shanghai, 200032 China; 4 Shanghai Institute for Advanced Immunochemical Studies and School of Life Science and Technology, ShanghaiTech University, Shanghai, 201210 China; 5 State Key Laboratory of Virology, Wuhan Institute of Virology, Center for Biosafety Mega-Science, Chinese Academy of Sciences, Wuhan, 430071 China; 6 School of Chinese Materia Medica, Nanjing University of Chinese Medicine, 138 Xian Lin Road, Jiangsu, 210023 Nanjing China; 7 School of Pharmaceutical Science and Technology, Hangzhou Institute for Advanced Study, UCAS, Hangzhou, 310024 China; 8 National Clinical Research Center for Infectious Disease, Shenzhen Third People’s Hospital, Shenzhen, 518112 China

**Keywords:** Structural biology, Preclinical research

## Abstract

Respiratory disease caused by coronavirus infection remains a global health crisis. Although several SARS-CoV-2-specific vaccines and direct-acting antivirals are available, their efficacy on emerging coronaviruses in the future, including SARS-CoV-2 variants, might be compromised. Host-targeting antivirals provide preventive and therapeutic strategies to overcome resistance and manage future outbreak of emerging coronaviruses. Cathepsin L (CTSL) and calpain-1 (CAPN1) are host cysteine proteases which play crucial roles in coronaviral entrance into cells and infection-related immune response. Here, two peptidomimetic α-ketoamide compounds, **14a** and **14b**, were identified as potent dual target inhibitors against CTSL and CAPN1. The X-ray crystal structures of human CTSL and CAPN1 in complex with **14a** and **14b** revealed the covalent binding of α-ketoamide groups of **14a** and **14b** to C25 of CTSL and C115 of CAPN1. Both showed potent and broad-spectrum anticoronaviral activities in vitro, and it is worth noting that they exhibited low nanomolar potency against SARS-CoV-2 and its variants of concern (VOCs) with EC_50_ values ranging from 0.80 to 161.7 nM in various cells. Preliminary mechanistic exploration indicated that they exhibited anticoronaviral activity through blocking viral entrance. Moreover, **14a** and **14b** exhibited good oral pharmacokinetic properties in mice, rats and dogs, and favorable safety in mice. In addition, both **14a** and **14b** treatments demonstrated potent antiviral potency against SARS-CoV-2 XBB 1.16 variant infection in a K18-hACE2 transgenic mouse model. And **14b** also showed effective antiviral activity against HCoV-OC43 infection in a mouse model with a final survival rate of 60%. Further evaluation showed that **14a** and **14b** exhibited excellent anti-inflammatory effects in Raw 264.7 mouse macrophages and in mice with acute pneumonia. Taken together, these results suggested that **14a** and **14b** are promising drug candidates, providing novel insight into developing pan-coronavirus inhibitors with antiviral and anti-inflammatory properties.

## Introduction

To date, seven human coronaviruses (HCoVs) have been identified: HCoV-229E, HCoV-OC43, HCoV-NL63, HCoV-HKU1, severe acute respiratory syndrome coronavirus (SARS-CoV), Middle East respiratory syndrome coronavirus (MERS-CoV), and SARS-CoV-2. Among these, the first four typically cause mild symptoms resembling the common cold. However, the remaining three are highly pathogenic and associated with severe disease and fatalities.^1,2^ The coronavirus disease 2019 (COVID-19) pandemic caused by SARS-CoV-2 is raging globally with deaths in the millions (https://covid19.who.int). Significant efforts have been dedicated to the development of effective prevention and treatment strategies for COVID-19. Several vaccines and antiviral drugs have been authorized, and widespread vaccination campaigns, together with the availability of antiviral drugs, have largely reduced morbidity and mortality. Nonetheless, antigenically distinct variants of wild-type SARS-CoV-2 have consistently emerged with enhanced transmissibility and/or pathogenicity, such as Omicron and its sublineages, potentially compromising the efficacy of existing vaccines and antiviral treatments.^3–5^ Instances of transmissible SARS-CoV-2 variants with resistance to existing SARS-CoV-2 main protease (M^pro^) inhibitors have occurred in the real world,^6^ and many cases of SARS-CoV-2 rebound infection have been reported after completing the recommended course of Paxlovid.^7^ In addition, MERS-CoV infection cases continue to be reported from the Middle East region,^8^ and HCoVs are estimated to account for 15–30% of common cold.^9^ Furthermore, it is concerning that animal coronaviruses may undergo spillover infection, jumping the species barrier in the future.^10,11^ This calls for the immediate development of pan-coronavirus inhibitors that meet clinical standards for the treatment of current SARS-CoV-2 infection, as well as its variants of concern, while in order to manage potential future outbreaks of zoonotic coronaviruses.

Despite continuous strides in the development of direct-acting anticoronavirus drugs, resistance and the lack of broad-spectrum profiles are still essential issues to resolve. Host-targeting therapeutics and prophylactics provide promising strategies to limit resistance and manage emerging coronaviruses. Host proteases are implicated in multiple stages of coronavirus infection, indicating their potential as ideal targets for the development of pan-coronavirus drugs.^12^ Coronaviruses release their genome into host cell cytoplasm after attachment to host surface-specific receptors. To accomplish this, coronaviruses rely on the activation of their enveloped glycoproteins by host proteases, including lysosomal CTSL and transmembrane protease serine 2 (TMPRSS2).^13,14^ Genomic RNA of coronaviruses can enter *via* both endosomal and non-endosomal pathways, depending on the distribution of proteases involved in spike (S) protein activation. The endosomal pathway is taken when CTSL proteolytically activates S protein within lysosome to facilitate fusion of viral envelope and lysosomal membrane. Accumulated evidence has shown that the Omicron variant is more dependent on the CTSL-mediated endosomal pathway.^15,16^ Although the detailed mechanism is unknown, evidence suggested that calpains, another class of intracellular calcium-activated cysteine proteases, also play a key role in the early steps of the coronaviral life cycle.^17,18^ As such, both proteases are potential targets for broad-spectrum antiviral intervention.

Dysregulated immune response to viral infection is a prominent feature of highly pathogenic coronaviruses.^19,20^ CTSL participates in priming innate and adaptive immunity by controlling the secretion of cytokines, antigen processing and presentation within antigen-presenting cells (APCs), and processing with different cytokine receptors.^21^ Upon viral infection-induced inflammatory conditions, CTSL in excessive amounts is activated and secreted into the extracellular environment for the purpose of recruiting immune cells to inflamed tissues, potentially resulting in the so-called “cytokine storm” and resultant uncontrolled inflammation that is strongly implicated in multiple organ injury. A recent study found that the circulating level of CTSL was elevated in COVID-19 patients, precisely reflecting the progression and severity of COVID-19.^22^ More strong evidence demonstrated that Cas13d knockdown of lung CTSL in mice could prevent and treat SARS-CoV-2 infection by decreasing lung virus load and improving virus-induced pathological changes.^23^ In addition to CTSL, calpains are also involved in the inflammatory process through regulating immune cell migration, modulating the activation of inflammatory mediators and degrading apoptosis-associated proteins.^24^ Viral infection affects ionic flow and membrane integrity, leading to calcium influx into cells to activate calpains, thereby triggering calcium channels to further promote calcium influx and inhibit calcium efflux.^25,26^ Increased intracellular calcium concentration is related to inflammation and cell death. In COVID-19, the low serum calcium level is closely correlated with clinical severity and induction of the primary inflammatory cytokine IL-6.^27^

The multiple roles of CTSL and calpains in the establishment and progression of viral pathophysiology highlight the importance of developing protease-targeting bispecific inhibitors with both antiviral and anti-inflammatory effects to prevent and treat coronavirus infection-related diseases. Recently, there have been several reports on the effective coronavirus inhibitors that target host CTSL and/or CAPN1 (Supplementary Scheme S1).^17,28–30^ Most of them were identified through drug repurposing efforts. Several promising repurposed drugs are undergoing clinical trials for the treatment of COVID-19, e.g., CTSL inhibitor SLV213 (https://clinicaltrials.gov/ct2/show/NCT04843787), which was originally developed as a therapeutic for the treatment of Chagas disease,^31^ and calpains inhibitor BLD2660 (https://clinicaltrials.gov/ct2/show/results/NCT04334460), which was originally developed for the treatment of fibrotic diseases. Among these repurposed drugs, several compounds, including MG-132, calpain inhibitor I, III and XII (Supplementary Scheme S1), exhibit potential host CTSL and CAPN1 dually inhibitory activity. However, a mismatch has arisen between their potent enzyme inhibitory activity and relatively limited antiviral activity, indicating the potential shortcomings with drug-like profile, such as cell permeability. Moreover, these inhibitors are still in the early stages of biologically testing and lack comprehensive medicinal evaluation. Therefore, whether human CTSL and CAPN1 dual inhibitors confer antiviral and anti-inflammatory activity remains unclear. Here, for the first time, two peptidomimetic α-ketoamide compounds, **14a** and **14b**, were developed as potent dual inhibitors targeting both host CTSL and CAPN1 with IC_50_ values at low nanomolar level based on structure-based drug design. The crystal structures of human CTSL and CAPN1 in complexes with **14a** and **14b** unveiled the detailed binding modes, offering a solid structural foundation for the development of host-targeting antivirals within this class. In addition, these complexes contributed to a better understanding of the underlying mechanism of the aforementioned dual inhibitors. The in vitro antiviral tests demonstrated both compounds as potent pan-coronavirus inhibitors, notably displaying low nanomolar potency against SARS-CoV-2 and its variants (VOCs). Furthermore, **14a** and **14b** showed favorable oral pharmacokinetic and safety properties in vivo. Additionally, treatment with both **14a** and **14b** demonstrated potent anti-SARS-CoV-2 efficacy in a SARS-CoV-2 XBB 1.16 variant infection K18-hACE2 transgenic mouse model, resulting in a significant reduction in lung viral load. And **14b** also exhibited effective antiviral activity in an HCoV-OC43 infection mouse model, achieving a final survival rate of 60%. Additionally, both **14a** and **14b** displayed excellent anti-inflammatory activity in the inflammation models using RAW 264.7 mouse macrophages and mice. The extensive and robust results suggest that human CTSL and CAPN1 dual inhibitors possess potent antiviral and anti-inflammatory activity in vitro and in vivo. In summary, this work demonstrated that **14a** and **14b** are promising drug candidates and provided novel insight into the development of pan-coronavirus inhibitors with both antiviral and anti-inflammatory properties.

## Results

### Design, synthesis, and preliminary structure–activity relationship exploration of the dual inhibitors

**Fig. 1:**
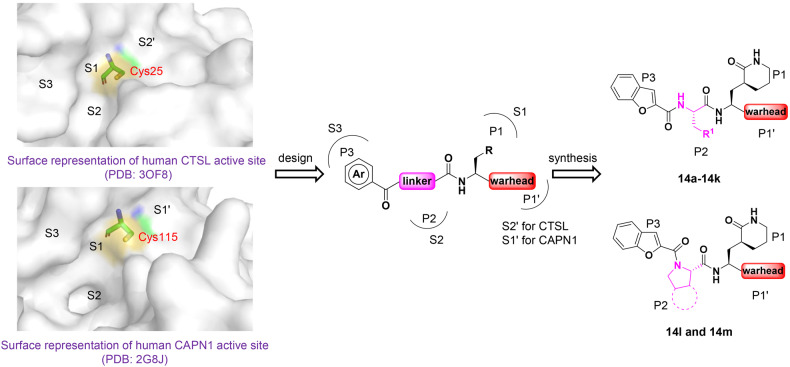
Design strategy of dual inhibitors of human CTSL and CAPN1

Four similar subsites,^32–35^ including S3, S2, S1, and S2’ (CTSL) or S1’ (CAPN1), have been identified in catalytically active clefts of human CTSL (PDB ID: 3OF8) and CAPN1 (PDB ID: 2G8J) (Fig. 1). Both proteases are thiol-dependent endopeptidases with a Cys-His catalytic dyad for CTSL and a Cys-His-Asn catalytic triad for CAPN1 at the active sites. Furthermore, the thiol group of the reactive cysteine residues could covalently anchor the electrophilic warhead, which is poised to enable the development of potent covalent inhibitors. Therefore, it is possible to design dual inhibitors targeting CTSL and CAPN1. The rational drug design for the peptidomimetic protease inhibitors by mimicking their native substrates or catalytic transition state is a well-established strategy. In order to occupy four subsites of the two proteases (S3, S2, S1 and S2’ (CTSL) or S1’ (CAPN1)), dipeptidyl covalent inhibitors composed of four fragments (P3, P2, P1 and P1’, respectively) were designed and synthesized. Based on the analysis of crystal structure of both proteases, the S1 and S3 subsites are relatively wide and unrestricted, which could tolerate a wide variety of substitutes. The S2 subsites are strongly hydrophobic and deep, indicating their essential roles for specificity. The S2’ subsite of CTSL and S1’ of CAPN1 containing nucleophilic cysteine residues are shallow and large, which are important for maintain inhibitory activity *via* covalently anchoring with compounds. Therefore, in design of dual inhibitors, we fixed the P1 and P3 fragments and allowed P1’ and P2 fragments to change. The flexible (*S*)-δ-lactam ring was chosen as P1 to form potential hydrogen bond with S1 subsites and benzo-2-furanyl group was introduced into P3 to enhance drug-like properties and occupy S3 subsites. Initially, the benzyl α-ketoamide moiety was used as electrophilic warhead in P1’ to covalently bind to the catalytic cysteines, which is essential for the inhibitory activity. Compared to small warhead, the bulky benzyl α-ketoamide was expected to afford additional hydrophobic interaction with targets and α-ketoamide moiety has been successfully applied in approved HCV NS3/4 A protease inhibitors, such as Telaprevir and Boceprevir.^36–38^ Then, the hydrophobic and large 4-fluorophenyl and cyclohexyl group were introduced into P2 to occupy S2 subsites, generating compounds **14a** and **14b**, respectively. A fluorescence resonance energy transfer (FRET)-based cleavage assay was used to evaluate their enzymatic inhibitory activity against human CTSL, and the results showed that **14a** and **14b** exhibited potent inhibitory activity against human CTSL with IC_50_ values of 3.34 nM and 6.88 nM, respectively (Table 1, Supplementary Fig. S1 and Table S1). Subsequently, a range of warheads with different size and electrical property, including vinyl sulfone, hydrazone, and Michael receptor derived from α-fluoro-acrylic acid, were introduced into compounds **14a** and **14b** to displace benzyl α-ketoamide moiety, and the results indicate that the inhibitory activity of the compounds against human CTSL is significantly reduced or even lost. Finally, in order to improve oral pharmacokinetic profiles, we aimed to reduce hydrogen bond donor (HBD) count *via* cyclizing the side chain with main chain NH group, as HBD count has been negatively correlated with oral bioavailability. However, the results demonstrated that cyclized P2 fragment showed a significant loss of inhibitory activity against human CTSL compared to compounds **14a** and **14b**, may be due to the rigid P2 fragments, which are incompatible with deep S2 subsite (Supplementary Table S1). Therefore, compounds **14a** and **14b** were selected to further study, and the assessment of their inhibitory activity against human CAPN1 revealed that these two compounds had potent activity against human CAPN1 with IC_50_ values of 375.1 nM and 347.6 nM for **14a** and **14b**, respectively (Table 1 and Supplementary Fig. S1).

**Table 1:** Enzymatic inhibitory and anticoronaviral activity of **14a** and **14b**^a^

Enzymatic inhibitory activity (IC_50_)				
Target		Unit	14a	14b
Human CTSL		nM	3.34 ± 0.52	6.88 ± 0.81
Human CAPN1		nM	375.1 ± 49.8	347.6 ± 34.0

Broad-spectrum anticoronaviral activity (EC_50_)				
Virus	Cell line	Unit	14a	14b
SARS-CoV-2 PsV	293 T/ACE2	nM	3.33 ± 1.90	0.61 ± 0.06
SARS-CoV PsV	Huh-7	nM	8.24 ± 2.98	10.06 ± 7.77
MERS-CoV PsV	Huh-7	nM	29.75 ± 5.72	13.57 ± 3.05
HCoV-229E PsV	Huh-7	nM	18.80 ± 4.18	13.05 ± 6.26
SARS-CoV-2	Vero-E6	nM	51.70 ± 10.78	< 40
HCoV-229E	Huh-7	nM	18.99 ± 4.17	20.58 ± 8.19
HCoV-OC43	RD	μM	3.38 ± 2.07	1.31 ± 0.32
B.1.351 (Beta)	Vero-E6	nM	161.7 ± 5.3	58.12 ± 1.07
B.1.617.2 (Delta)	Vero-E6	nM	10.98 ± 1.80	28.61 ± 3.79
B.1.617.2 (Delta)	293T/ACE2-TMPRSS2	nM	0.80 ± 0.16	0.84 ± 0.13

^a^Each sample was tested in triplicate unless otherwise indicated. Data are shown as mean ± SD

### Off-target selectivity profile of **14a** and **14b**

To assess the off-target selectivity profile of these two compounds, screen of a broad panel of host proteases was carried out. The compounds **14a** and **14b** demonstrated a favorable off-target selectivity profile in a series of human proteases (Supplementary Fig. S1 and Table S2). Among the 13 human proteases tested, both compounds only showed potent inhibitory activity against human cathepsin B (CTSB) with IC_50_ values of 1.23 nM and 1.01 nM for **14a** and **14b**, respectively. CTSB is another important lysosomal cysteine protease involved in the infection of diverse viruses and host immune response.^21,39^ However, the results showed that **14a** and **14b** did not exhibit inhibitory activity against TMPRSS2 (at a concentration of 10 μM) and furin (at a concentration of 20 μM), two serine proteases involved in coronaviral entrance. Several important viral targets related to coronavirus infections were also studied. The results showed that **14a** and **14b** had weak inhibitory activity against SARS-CoV-2 M^pro^ with IC_50_ values of 12.57 μM and 0.99 μM, respectively. They also showed very weak inhibitory activity against SARS-CoV-2 RNA-dependent RNA polymerase (RdRp) with IC_50_ values at micromolar levels. Finally, no inhibitory activity occurred against papain-like protease (PL^pro^) at a concentration of 20 μM for **14a** and **14b**, respectively.

### Structures of human CTSL in complex with **14a** and **14b**, respectively

**Fig. 2:**
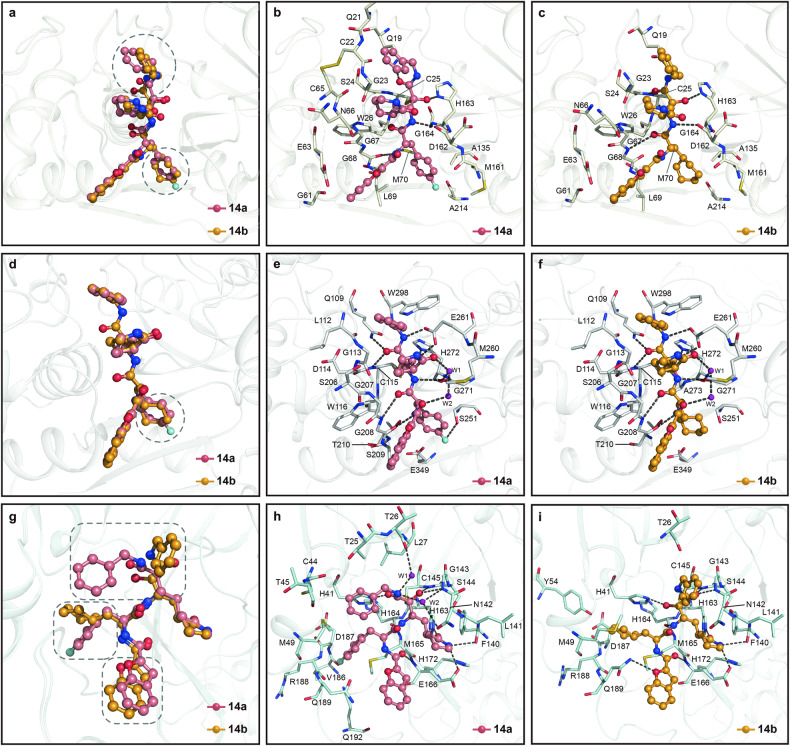
Crystal structures of human CTSL, CAPN1 protease core, and SARS-CoV-2 M^pro^ in complex with **14a** and **14b**, respectively. **a** A comparison of the binding modes of **14a** and **14b** in human CTSL substrate-binding pocket. The major difference between both compounds is marked with a dashed rounded circle. Both compounds are shown as ball-and-stick models with the carbon atoms in salmon **(14a)**, bright orange **(14b)**, oxygen atoms in bright red, nitrogen atoms in blue, and fluorine atom in cyan (the same as below, unless otherwise indicated). **b**, **c** Close-up view of inhibitor binding pocket for **14a** (**b**) and **14b** (**c**) in human CTSL. Residues involved the binding of inhibitors are shown as milky sticks. Hydrogen bonds are indicated as dashed lines. **d** A comparison of the binding modes of **14a** and **14b** in human CAPN1 protease core substrate-binding pocket. The major difference between both compounds is marked with a dashed rounded circle. **e**, **f** Close-up view of the binding pocket of **14a** (**e**) and **14b** (**f**) in human CAPN1 protease core. Residues involved in the binding of inhibitors are shown as light gray sticks. Hydrogen bonds are indicated as dashed lines. **g** A comparison of the binding modes of **14a** and **14b** in SARS-CoV-2 M^pro^ substrate-binding pocket. The major difference between both compounds is marked with a dashed rounded rectangle. **h**, **i** Close-up view of the binding pocket of **14a** (**h**) and **14b** (**i**) in SARS-CoV-2 M^pro^. Residues involved in the binding of inhibitors are shown as pale-cyan sticks. Hydrogen bonds are indicated as dashed lines

To elucidate the mechanism underlying the potent inhibition of human CTSL by **14a** and **14b**, the crystal structures of human CTSL in complex with these two compounds were solved at 2.4 Å and 2.9 Å resolution, respectively (Fig. 2a–c, Supplementary Fig. S2 and Table S3). The structures of both complexes belong to the space group of *I*422 and contain one CTSL molecule in an asymmetric unit (ASU). The overall structure of these two complexes is similar, except for several flexible loops. CTSL, which belongs to the papain superfamily, adopts a cysteine protease fold.^32,40^ The overall structure of mature CTSL is composed of two domains, and the active site is located at the ‘V’-shaped cleft between them (Supplementary Fig. S2a–c). Domain I is an α-helix-rich domain that contains three α-helices and one β-sheet. Domain II is a β-barrel fold domain that consists of five β-sheets and one α-helix. C25 from domain I and H163 from domain II constitute a catalytic dyad at the substrate-binding site.^32^ According to unambiguous electron density maps, these two inhibitors are covalently linked to catalytic C25 with similar binding modes.

As a peptidomimetic inhibitor, compound **14a** occupies the S1, S2, S3, and S2′ subsites of human CTSL (Supplementary Fig. S2d). The thiol group of catalytic C25 attacks the electrophilic α-keto group of the α-ketoamide warhead, resulting in the formation of a thiohemiketal. Apart from covalent interaction, four hydrogen bonds can be identified in the complex structure (Fig. 2b and Supplementary Fig. S2e, f). At S1 subsite, the hydroxyl group of this thiohemiketal forms one hydrogen bond with the Nδ atom of H163. Additionally, the amide oxygen of the α-ketoamide warhead interacts with the Nε2 atom of Q19 through a hydrogen bond. The NH group at the P1 site also forms a hydrogen bond with the carbonyl oxygen of D162. The (*S*)-δ-lactam ring at the P1 site is surrounded by the main chains of G23, W26, C65, N66, and G67, as well as the side chain of W26 at the S1 subsite, through hydrophobic interactions. The benzyl group at P2′, which is surrounded by the backbone parts of Q21, C22, and G23, interacts with CTSL mainly through hydrophobic interactions. As expected, the deep hydrophobic S2 subsite, which is formed by the side chains of L69, M70, A135, and M161 and the main chains of M161, D162, H163, and G164, was occupied by the 4-fluorophenyl group at P2 through extensive hydrophobic interactions. In addition, the NH group at the P2 site plays a crucial role in inhibitor recognition by forming one hydrogen bond with the backbone carbonyl oxygen of G68. The 2-benzofuranyl group at P3, which is surrounded by G61, E63, G67, G68, and L69, fits well into the S3 subsite through hydrophobic interactions.

The crystal structure of CTSL in complex with **14b** is quite similar to that of CTSL in complex with **14a** (Fig. 2a–c and Supplementary Fig. S2). The diverse binding modes are most likely caused by the cyclohexyl group at the P2 site. Compared with the 4-fluorophenyl in **14a**, the cyclohexyl group has less steric hindrance and extends into the S2 subsite. Thus, the whole inhibitor moves slightly downward in comparison with **14a** (Fig. 2a and Supplementary Fig. S2b, c). The side chains of residues L69, A135, M161, and A214 and the main chain parts of M161 and D162 stabilize the cyclohexyl ring through hydrophobic interactions. Compared with the complex structure of CTSL-**14a**, the carbonyl oxygen at the P2 site forms one hydrogen bond with the backbone NH of G68. The amide oxygen of the α-ketoamide warhead in the complex structure of CTSL-**14b** occupies the oxyanion hole and forms one hydrogen bond with the backbone NH of C25.

### Structures of human CAPN1 protease core in complex with **14a** and **14b**, respectively

The crystal structures of human CAPN1 protease core in complex with **14a** and **14b** were solved at 1.6 Å and 1.8 Å resolution, respectively (Fig. 2d-f, Supplementary Fig. S3 and Table S3). Both complex structures belong to the space group of *P*2_1_2_1_2_1_ and have similar unit cell parameters. Only one CAPN1 protease core molecule was observed in an ASU. CAPN1 also belongs to the papain superfamily which is featured with the classical mixed α/β structure. The overall structure of CAPN1 protease core also contains two domains (domain I: aa33-219, domain II: aa220-353). The substrate binding site features a catalytic triad (C115, H272, and N296) that lies in the deep cleft between these two domains.^34^ Similar to the aforementioned complex structures, the catalytic cysteine C115 anchors these two inhibitors to the active site through covalent linkage.

Compound **14a** occupies the S1, S2, S3, and S1′ subsites of human CAPN1 protease core (Supplementary Fig. S3d). Apart from covalent interaction, twelve hydrogen bonds are formed between **14a** and the CAPN1 protease core (Fig. 2e, f and Supplementary Fig. S3e, f). At the S1 subsite, the hydroxyl group of the thiohemiketal forms hydrogen bonds with the Oε2 atom of E261 and the Nδ1 atom of H272, respectively. The NH group at P1 position forms a hydrogen bond with the carbonyl oxygen of G271. The (*S*)-δ-lactam ring is surrounded by S206, G207, M260, and E261 through hydrophobic interactions. The carbonyl oxygen of (*S*)-δ-lactam ring forms a hydrogen bond with the main chain carbonyl oxygen of G271 through an ordered water molecule. The amide oxygen of the α-ketoamide warhead forms hydrogen bonds with the Nε2 atom of Q109 and the backbone NH group of C115, respectively. The amide nitrogen of the α-ketoamide group at P1’ site also forms a hydrogen bond with the Oε1 atom of E261. The P1’ benzyl group is surrounded by the main chains of L112 and G113, as well as the side chains of Q109, E261, and W298, and it interacts with the CAPN1 protease core through hydrophobic interactions. S2 is a deep hydrophobic subsite, which can sufficiently accommodate the 4-fluorophenyl group. Residues W116, S209, T210, S251, G271, H272, and E349 encompass the 4-fluorophenyl group and stabilize this site through extensive hydrophobic interactions. The NH group at P2 site forms a hydrogen bond with the backbone carbonyl oxygen of G208. The carbonyl at this position also interacts with the backbone NH group of G206 through a hydrogen bond. Besides, another hydrogen bond is formed between the fluorine atom of the 4-fluorophenyl group and the Oγ atom of S251. The 2-benzofuranyl fits into the S3 subsite and interacts with the main chains of G207, G208, and S209 through hydrophobic interaction. The carbonyl oxygen beside the 2-benzofuranyl group interacts with the backbone carbonyl group of G271 through two ordered water molecules.

The binding modes of human CAPN1 protease core in complex with **14b** are very similar to those of CAPN1 protease core in complex with **14a** (Fig. 2d–f and Supplementary Fig. S3). The single difference results from the 4-fluorophenyl group of **14a** at P2 position. Compared with the 4-fluorophenyl group of **14a**, the cyclohexyl group in **14b** cannot form hydrogen bonds with S251.

### Structures of SARS-CoV-2 M^pro^ in complex with **14a** and **14b**, respectively

Previous assessments found that **14a** and **14b** also showed weak inhibitory activity against SARS-CoV-2 M^pro^ with IC_50_ values at micromolar levels (Supplementary Fig. S1 and Table S2). In order to delineate the inhibitory mechanism of **14a** and **14b**, the crystal structures of SARS-CoV-2 M^pro^ in complex with these two compounds were solved at 1.7 Å and 1.6 Å resolution, respectively (Fig. 2g-i, Supplementary Fig. S4 and Table S3). The overall structure of these two complexes is similar to that of numerous previously reported structures, forming a functional dimer.^41–43^ Each protomer consists of two domains, and the substrate-binding pocket is located in the cleft between domain I and domain II (Supplementary Fig. S4a–c). H41 from domain I and C145 from domain II form a catalytic dyad indispensable for hydrolysis. As peptidomimetic inhibitors with α-ketoamide-reactive warheads, both compounds localized to the substrate-binding pocket and covalently linked to C145 to inhibit the protease activity of SARS-CoV-2 M^pro^.

Potent compound **14b** can be clearly traced according to the electron density map. Compared with compound **14a**, it has stronger inhibitory activity (Supplementary Fig. S1 and Table S2). The thiol group of catalytic C145 attacks the electrophilic α-keto group of **14b**, resulting in the formation of a thiohemiketal (Fig. 2i and Supplementary Fig. S4h, i). A standard 1.8 Å C-S covalent bond is then formed between the carbon atom of the α-keto group and the Sγ atom of C145, which can be unambiguously observed in the electron density map. Overall, compound **14b** occupies the S1, S2, S4, and S1′ subsites of SARS-CoV-2 M^pro^ consistent with our design (Supplementary Fig. S4g–i). At the S1 subsite, the hydroxyl group of this thiohemiketal forms one hydrogen bond with the Nδ atom of H41. Meanwhile, the amide oxygen of the α-ketoamide warhead occupies the oxyanion hole stabilized by the backbone NH group of C143 and C145 through two hydrogen bonds. The NH group at S1 subsite also interacts with the backbone carbonyl oxygen of H164 through a hydrogen bond. Similar to the localization of glutamine during polyprotein processing, three hydrogen bonds are also involved in the localization of the designed (*S*)-δ-lactam ring. The carbonyl oxygen of (*S*)-δ-lactam ring forms a hydrogen bond with the Nε2 atom of H163. Additionally, the NH group of the (*S*)-δ-lactam ring forms hydrogen bonds with the carbonyl oxygen of F140 and the Nε2 atom of E166, respectively. The benzylamine group at P1′ position is surrounded by T26, N142, and G143 and stabilized by extensive hydrophobic interactions. The cyclohexyl moiety of compound **14b** is stabilized by the imidazole ring of H41 through π-π stacking interaction and is surrounded by the side chains of H41, Y54, M165, and Q189 and the main chains of D187 and R188 through hydrophobic interactions, fitting well into the deep hydrophobic S2 subsite. The designed benzofuran-2-carbonyl group at P3 position occupies the S4 subsite. The carbonyl oxygen of the benzofuran-2-carbonyl group interacts with the backbone NH group of E166 through a hydrogen bond. Additionally, the 2-benzofuranyl group interacts with the side chain of Q189 through another hydrogen bond.

The crystal structure of SARS-CoV-2 M^pro^ in complex with **14a** is similar to that of the structure of M^pro^-**14b** complex with an RMSD (Root Mean Square Deviation) value of 0.103 Å (Fig. 2g–i and Supplementary Fig. S4). The binding modes of **14a** and **14b** are very close, except for the P2 and P1’ positions (Fig. 2g and Supplementary Fig. S4b, c). Compared to the cyclohexyl moiety at the P2 position, the 4-fluorophenyl moiety of **14a** exhibits a linear distribution and does not fit well into the S2 subsite (Fig. 2h and Supplementary Fig. S4d-f). Consequently, the π-π stacking interaction with the imidazole ring of H41 is disrupted, and the inhibitor molecule is slightly shifted outward by 0.5 Å. The hydrogen bond interactions between the 2-benzofuranyl group and the side chain of Q189 also disappeared. Owing to strong interactions at the S1 subsite, this positional shift has limited influence on the (*S*)-δ-lactam ring at the P1 position (Fig. 2g and Supplementary Fig. S4b, c). However, the positions of the NH group, hydroxyl group, and carbonyl group at P1 position were all shifted. Compared with compound **14b**, the hydroxyl group of the thiohemiketal occupies the oxyanion hole and forms hydrogen bonds with C143 and C145, respectively (Fig. 2h and Supplementary Fig. S4d–f). Correspondingly, the remaining amide oxygen of the α-ketoamide warhead interacts with the side chain of H41 and forms a hydrogen bond. Interestingly, the benzyl group of **14a** is flipped backward nearly 90 degrees and surrounded by the side chains of T25, L27, H41, and M49 and the main chains of C44 and T45 (Fig. 2h and Supplementary Fig. S4d-f). The NH group of the benzylamino group also interacts with M^pro^ through two ordered water molecules.

### **14a** and **14b** showed broad-spectrum anticoronaviral activity

CTSL and CAPN1 play significant roles in the entry and infection of multiple enveloped viruses and thus may serve as ideal targets for inhibition of diverse coronaviruses. Thus, we investigated whether **14a** and **14b** possess broad anticoronaviral activity against various coronaviruses. Based on our coronavirus pseudovirus (PsV) inhibition assay,^44,45^ we found that **14a** potently and broadly inhibited pseudotyped SARS-CoV-2, SARS-CoV, MERS-CoV, and HCoV-229E infection with EC_50_ values ranging from 3.33 nM to 29.75 nM (293 T/ACE2 cells for SARS-CoV-2 PsV and Huh-7 cells for other PsVs). Similarly, **14b** also potently and broadly inhibited infection of various pseudotyped HCoVs with EC_50_ values ranging from 0.61 nM to 13.57 nM in corresponding cells (Table 1**and** Supplementary Fig. S5). Next, we assessed authentic coronavirus inhibitory activity of both compounds,^46,47^ and the results showed that **14a** and **14b** could potently inhibit infection by authentic SARS-CoV-2 wild-type strain (Vero-E6 cells) with EC_50_ values of 51.70 nM and less than 40 nM, respectively. Meanwhile, both compounds had potent and pan-HCoV inhibitory activity against HCoV-229E (Huh-7 cells) and HCoV-OC43 (RD cells) strains with EC_50_s ranging from 18.99 nM to 3.38 μM. Host-targeting antivirals have an advantage in overcoming drug resistance resulting from viral mutations as compared to direct-acting antivirals. To prove this, we tested the inhibitory activity of **14a** and **14b** against two prevalent SARS-CoV-2 VOCs, B.1.351 (Beta) and B.1.617.2 (Delta) variants, in Vero-E6 cells. The results showed that **14a** and **14b** could inhibit Beta variant infection with EC_50_ values of 161.7 nM and 58.12 nM, respectively. Both compounds could potently block Delta variant infection of host cells with EC_50_ of 10.98 nM and 28.61 nM, respectively. Since Vero-E6 cells lack the TMPRSS2-mediated non-endosomal entry pathway, it is possible that the antiviral activity of both compounds might be overestimated. Therefore, we conducted additional experiments to assess the efficacy of **14a** and **14b** in human ACE2-TMPRSS2-expressing HEK293T cells (293T/ACE2-TMPRSS2) infected with the Delta variant. The results demonstrated a potent inhibitory activity against Delta variant with EC_50_ values of 0.80 nM and 0.84 nM for compounds **14a** and **14b**, respectively. These results strongly demonstrated that compounds **14a** and **14b** possess potent and broad-spectrum anticoronaviral activity. Meanwhile, the Cell Counting Kit-8 (CCK8) assay was used to assess the cytotoxicity, and the results demonstrated that **14a** and **14b** displayed very low or no cytotoxicity on the cell lines mentioned above (Supplementary Fig. S6).

### Mechanistic exploration of **14a** and **14b** harboring broad-spectrum and excellent anticoronaviral effects

**Fig. 3:**
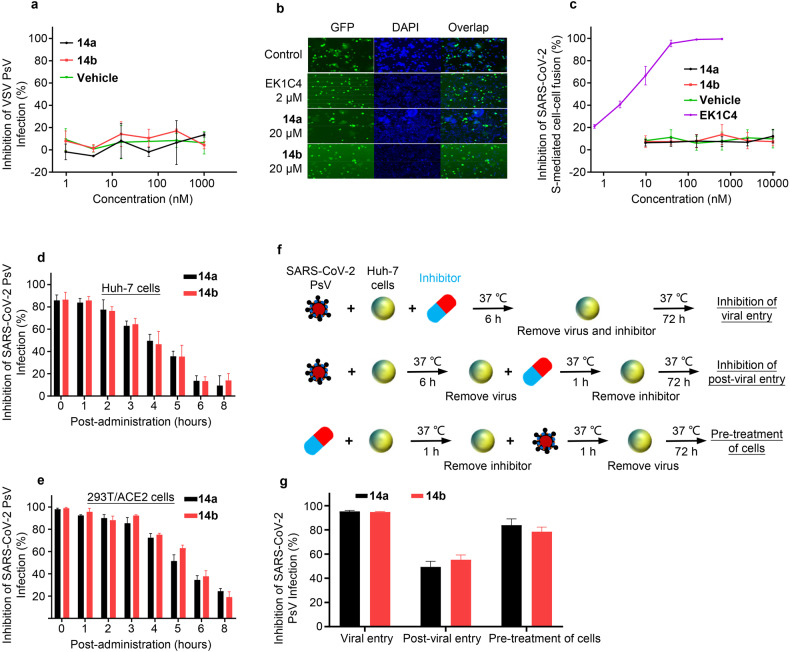
Mechanistic exploration of **14a** and **14b** harboring excellent anticoronaviral effects. **a** **14a** and **14b** have no inhibitory activity on VSV-G protein-mediated entry. **b** Representative pictures of SARS-CoV-2 S protein-mediated cell-cell fusion experiments. **c** Inhibitory curve of compounds against SARS-CoV-2 S protein-mediated cell-cell fusion. **d**, **e** Compounds lost most inhibitory activity 5 h after infection in Huh-7 and 293T/ACE2 cells, respectively. **f** Scheme of time-of-addition experiments for Fig. 3g. **g** Compounds inhibit viral entry, but not post-viral entry stage. Data are represented as mean ± SEM

We previously noted that CTSL could facilitate the entry of virus into the host cell by the endosomal route, as preliminarily confirmed by the highly effective inhibition of **14a** and **14b** against various HCoVs PsV (Table 1 and Supplementary Fig. S5). To further investigate the effect of **14a** and **14b** on the viral entry process, we tested their inhibitory activity on vesicular stomatitis virus G glycoprotein (VSV-G) mediated entry, and the results showed that **14a** and **14b** had no inhibitory activity (Fig. 3a), indicating that both compounds can effectively and selectively block the entry process of HCoVs into the host cell. Meanwhile, we also found that both compounds had no effect on SARS-CoV-2-S-mediated cell-cell fusion (Fig. 3b, c), suggesting that the target of both compounds is not the viral S protein, but rather other cellular proteins that play key roles in viral entry and infection. Furthermore, we used time-of-addition assays and found that both compounds lost most of their antiviral activity 5 h after infection by pseudotyped SARS-CoV-2 (Fig. 3d–g), strongly suggesting that the tested compounds may target the early stage of virus entry, possibly during the process of S protein activation by CTSL in the endocytic pathway, further highlighting cellular CTSL as their potential target.

### In vivo pharmacokinetic and toxicity studies of **14a** and **14b**

The pharmacokinetic (PK) properties of **14a** and **14b** were evaluated to explore further druggability, and a summary of PK parameters for **14a** and **14b** is shown in Supplementary Table S4. Both showed good PK properties after administration by intravenous (i.v.), intraperitoneal (i.p.), and oral (p.o.) routes. In mice, the area under the curve (AUC) values of **14a** were 1260, 1270, and 126 ng*h/mL after intravenous (5 mg/kg), intraperitoneal (10 mg/kg), and oral (10 mg/kg) administration, respectively. The corresponding values for **14b** were 2976, 7635, and 488 ng*h/mL when administered by intravenous (10 mg/kg), intraperitoneal (20 mg/kg) and oral (20 mg/kg) routes. After intraperitoneal administration, **14a** showed a slightly longer half-life (*t*_1/2_) than **14b** (7.01 h versus 5.77 h). The intraperitoneal bioavailability (*F*) of **14a** and **14b** was greater than 100% (101% and 128%, respectively), potentially caused by the saturation of metabolizing enzymes, or other reasons.^48^ Considering the factors of convenience and patient compliance of oral drugs, we placed more focus on the oral PK parameters. In rats, **14a** and **4b** exhibited relatively higher oral bioavailability of 13% and 32%, respectively. After oral (10 mg/kg) administration in rats, **14a** and **14b** showed good maximal concentration in plasma (*C*_max_) values (416 and 982 ng/mL, respectively), high AUC values (1060 and 2680 ng*h/mL, respectively) and acceptable *t*_1/2_ values (2.52 and 3.86 h, respectively). In dogs, **14a** and **14b** also exhibited considerable bioavailability with oral bioavailability values of 10%. When administered orally (5 mg/kg), **14a** and **14b** showed AUC values of 357 and 283 ng*h/mL, respectively. The above data demonstrated that compounds **14a** and **14b** showed good oral PK properties in mice, rats, and dogs, indicating that both compounds show promise for development as oral drugs for the treatment of broad-spectrum coronavirus infection.

Then in vivo acute toxicity studies of compounds **14a** and **14b** were carried out in C57 mice. No mice died after oral treatment (1000 mg/kg/day and 2000 mg/kg/day) with either **14a** or **14b**. And no abnormalities in body weights, food mean daily consumption, general status, and no obvious organs and tissues damage were observed (Supplementary Fig. S7 and Table S6). These data have demonstrated that compounds **14a** and **14b** possess favorable safety profile in vivo.

### In vivo antiviral efficacy of **14a** and **14b** against SARS-CoV-2 infection

**Fig. 4:**
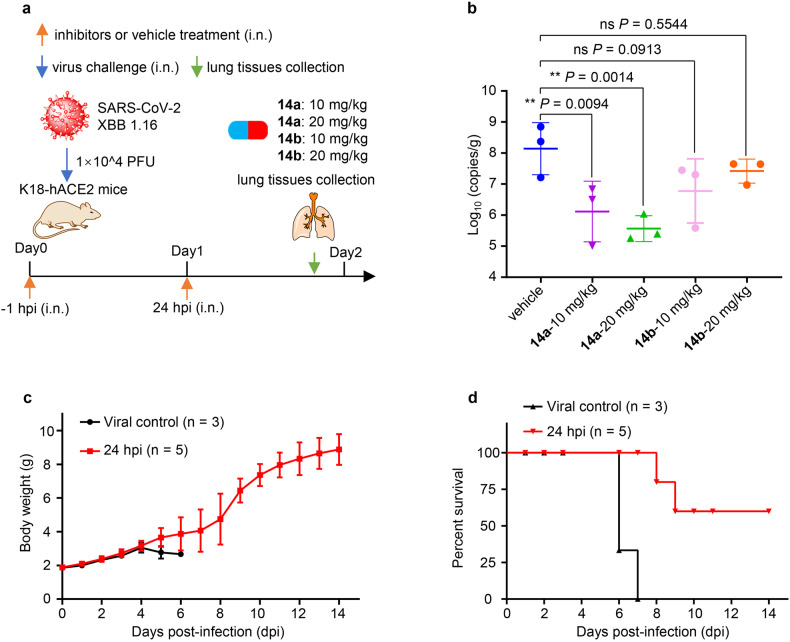
In vivo antiviral efficacy studies of compounds **14a** and **14b**. **a** Schematic diagram of in vivo antiviral potency study process of compounds **14a** and **14b** in K18-hACE2 mice challenged with SARS-CoV-2 XBB 1.16 variant. **b** Viral RNA copy numbers in the lung tissues of SARS-CoV-2 XBB 1.16 variant infected mice after two doses treatment with vehicle, compounds **14a** and **14b**. **c** Body weight changes of HCoV-OC43-infected mice after vehicle or compound **14b** treatment. **d** Survival rate of HCoV-OC43-infected mice after vehicle or compound **14b** treatment. Results are shown as mean ± SEM. **P < 0.01, by two-tailed, unpaired *t* test

We further investigated the efficacy of **14a** and **14b** in K18-hACE2 mice intranasally challenged with SARS-CoV-2 XBB 1.16 variant. After 1 h of compounds or vehicle intranasally treating, the mice were intranasally challenged by SARS-CoV-2 XBB 1.16 (1 × 10^4^ PFU per mouse, designated as day 0). Then, mice were administrated once at 24 hours post infection (hpi, day 1). On day 2, the mice were euthanized to take lung tissues for viral RNA detection (Fig. 4a). The results indicated an effective reduction in viral RNA copies in the lung tissues of the treatment groups receiving inhibitors compared to the vehicle group with a range from 0.7 log_10_ to 2.6 log_10_ units (Fig. 4b). Notably, compound **14a** demonstrated potent antiviral efficacy by significantly and dose-dependently decreasing the lung viral load by 2.0 log_10_ and 2.6 log_10_ units in the 10 mg/kg and 20 mg/kg dose groups, respectively, when compared to the vehicle group. These results demonstrated that compounds **14a** and **14b** had potent effect against SARS-CoV-2 XBB 1.16 variant in vivo.

### In vivo protective efficacy of **14b** against HCoV-OC43 infection

We also further assessed the in vivo antiviral activity of compound **14b** against HCoV-OC43 infection in a mouse model. In the HCoV-OC43 infection mouse model, we treated newborn mice with **14b** at a dose of 5 mg/kg by intraperitoneal injection 24 h after challenge with HCoV-OC43 at 100 TCID_50_. As shown in Fig. 4c, body weight of mice in the viral control group sharply decreased from 4 days post-infection (dpi), and all mice succumbed to death by 6 dpi. In contrast, body weight of mice in the **14b**-treatment group either mildly decreased or rapidly recovered, and the final survival rate reached 60% (Fig. 4d). These results suggested that **14b** has effective anticoronaviral activity in vivo.

### **14a** and **14b** exhibited excellent anti-inflammatory effects in vitro and in vivo

**Fig. 5:**
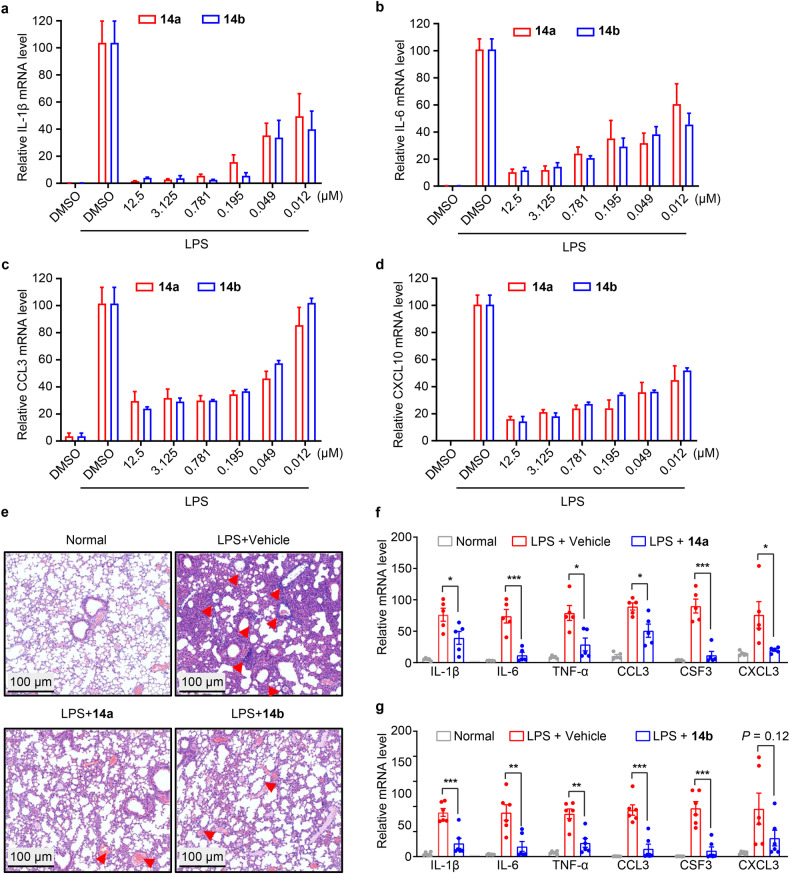
Anti-inflammatory effects of **14a** and **14b** in Raw 264.7 cells and in vivo. **14a** and **14b** inhibited LPS-induced transcription of IL-1β (**a**), IL-6 (**b**), CCL3 (**c**) and CXCL10 (**d**) in Raw 264.7 cells in a dose-dependent manner. **e** Representative images of lung histopathological changes from the indicated groups. The red arrows point to inflammatory cell infiltration. **f**, **g** Representative cytokine assessment of lung tissues of the indicated groups. Results are shown as mean ± SEM. *P < 0.05; **P < 0.01; ***P < 0.001; by 2-tailed, unpaired *t* test

Although the pathophysiology for coronaviruses, especially lethal ones, is poorly studied, it is speculated that acute lung injury mainly results from massive inflammation mediated by viral infection and replication. Infection from coronaviruses can lead to increased secretion of proinflammatory cytokines and chemokines by the so-called “cytokine storm” effect, as noted above. ICU patients with severe COVID-19 had higher plasma levels of IL-1β/2/6/7/10, TNFα, CSF3, CCL3, CCL3, and CXC10, suggesting that these excessive inflammatory responses are related to disease severity.^49^ To further evaluate whether compounds **14a** and **14b** could exert anti-inflammatory activity by dually inhibiting CTSL and CAPN1, we determined the potential anti-inflammatory effect of **14a** and **14b** by measuring their capacity to suppress lipopolysaccharide (LPS)-induced expression of proinflammatory cytokines (IL-1β, IL-6) and chemokines (CCL3, CXCL10). It was shown that **14a** and **14b** significantly inhibited LPS-induced expression of these proinflammatory cytokines and chemokines in a dose-dependent manner in Raw 264.7 mouse macrophages (Fig. 5a–d).

Based on the anti-inflammatory activity in mouse macrophages and good pharmacokinetic properties in vivo of **14a** and **14b**, we next evaluated the impact of **14a** and **14b** on inflammatory response in a mouse model of LPS-induced acute pulmonary inflammation. Twenty-four hours after induction, histopathological and gene expression analysis suggested that all LPS-challenged mice suffered from pneumonia, whereas **14a**- and **14b**-treated mice exhibited reduced inflammatory cell infiltration and alveolar septal thickening compared with vehicle-treated mice (Fig. 5e). Besides, **14a** and **14b** reduced the expression levels of inflammatory cytokines (IL-1β, IL-6, and TNFα) and chemokines (CCL3, CSF3, and CXCL10) in the lung tissues (Fig. 5f, g), suggesting that these two compounds ameliorate lung damage by affecting host immune response. Together, our results show that intraperitoneal administration of 100 mg/kg **14a** or **14b** could efficiently inhibit lung inflammation in mice with acute pneumonia.

## Discussion

Currently, SARS-CoV-2, MERS-CoV, and cold-causing HCoVs are still circulating around the world. New drugs with broad-spectrum anticoronaviral activity and anti-inflammatory activity are still urgently needed to prevent and treat coronavirus infection, especially for current SARS-CoV-2 variants. Drugs with multiple mechanisms of action have advantages in suppressing viral mutations and may have broad-spectrum antiviral activity. Human CTSL and CAPN1 are important drug targets because they play key roles in viral entry into host cells and virus infection-mediated “cytokine storm”. Therefore, dual inhibitors targeting host CTSL and CAPN1 might bring broad-spectrum synergistic efficacy to the fight against coronavirus in the future, while reducing excess inflammation to improve symptoms in patients with viral infections.

In this study, structure-based drug design was conducted to obtain dual-targeting inhibitors **14a** and **14b** against host CTSL and CAPN1. Both compounds potently inhibit human CTSL with IC_50_ values of single-digit nanomole. **14a** and **14b** also showed potent inhibitory activity against human CAPN1 at nanomolar levels. Structural information revealed that both compounds occupy the substrate-binding pockets of CTSL and CAPN1 protease core, blocking their protease activity by forming a covalent bond with the catalytic cysteine. In detail, both **14a** and **14b** occupy the S1, S2, S3, and S2′ subsites of human CTSL. Correspondingly, both compounds occupy the S1, S2, S3, and S1′ subsites of human CAPN1 protease core. Meanwhile, **14a** and **14b** also showed weak inhibitory activity against SARS-CoV-2 M^pro^ with IC_50_ values at micromolar levels. Interestingly, the crystal structures showed that compound **14b** occupies the S1, S2, S4, and S1′ subsites of SARS-CoV-2 M^pro^, while **14a** occupies the S1, S2, S4 subsites and the extended sub-pocket close to the S2 subsite. These structural data lay the foundation for structure-based design of multitarget inhibitors that simultaneously target host and viral proteins.

Further assessment demonstrated that **14a** and **14b** showed potent and broad-spectrum anticoronaviral activity against HCoV-229E, HCoV-OC43, SARS-CoV-2, and its variants prevalent around the world, including Beta and Delta variants. In the presence of TMPRSS2-mediated non-endosomal compensated entry pathway, we were delighted to find that compounds **14a** and **14b** also had excellent inhibitory activity against Delta variant with EC_50_ values of 0.80 nM and 0.84 nM, respectively, in human ACE2-TMPRSS2-expressing 293 T cells. Moreover, in K18-hACE2 mice intranasally challenged with SARS-CoV-2 XBB 1.16 variant, the treatment of **14a** and **14b** showed potent antiviral efficacy, leading to a significant reduction in viral load in lung tissues. Specifically, compound **14a** exhibited robust antiviral effectiveness, demonstrating a significant and dose-dependent decrease in lung viral load by 2.0 log_10_ and 2.6 log_10_ units in the 10 mg/kg and 20 mg/kg dose groups, respectively, compared to the vehicle group. In an HCoV-OC43-infected mouse model, **14b** also displayed effective antiviral activity with a final survival rate of 60%. Further experiments demonstrated that **14a** and **14b** could effectively suppress LPS-induced inflammation in Raw 264.7 mouse macrophages and exhibited anti-inflammatory effects in a mouse model of LPS-induced acute pulmonary inflammation. Both compounds revealed good oral pharmacokinetic properties in mice, rats, and dogs, and favorable safety in mice. Taken together, both compounds are promising drug candidates for the treatment of infections caused by coronaviruses.

The compromised antiviral efficacy of the anti-SARS-CoV-2 drugs authorized for clinical use, caused by the high-frequency mutations of SARS-CoV-2, underscores the urgent need for the development of broad-spectrum anticoronaviral drugs with multiple effects. Our work presents a typical paradigm for exploring host targets involved in viral infection and inflammatory response to discover broad-spectrum host-targeting antivirals with increased resistance to viral evolution and potential anti-inflammatory activity. These findings may prove beneficial for the prevention and treatment of future viral disease outbreaks.

## Materials and methods

### Animals

All procedures performed on animals were in accordance with regulations and established guidelines and were reviewed and approved by the Institutional Animal Care and Use Committee at Shanghai Institute of Materia Medica (SYXK2020-0042) and Wuhan Institute of Virology (SYXK2022-0131), Chinese Academy of Sciences, and the Animal Ethics Committee of Fudan University (20190221-070), following the National Institutes of Health Guidelines for the Care and Use of Experimental Animals (WIVA25202202).

### Cell lines

293T, Vero-E6, and Raw 264.7 cell lines were obtained from the American Type Culture Collection (ATCC). Huh-7, RD, and 293T stably expressing human ACE2 and TMPRSS2 (293T/ACE2-TMPRSS2) cell lines were purchased from the cell bank of CAS. 293T cells stably expressing human ACE2 (293 T/ACE2) were kindly provided by Dr. Lanying Du. All cells were maintained in Dulbecco’s Modified Eagle’s Medium (DMEM) containing 10% FBS. Cells were incubated at 37 °C under 5% (v/v) CO2 atmosphere.

### Viruses

The HCoV-OC43 strain (VR-1558) and HCoV-229E strain (VR-740) were purchased from ATCC and kept in our laboratory. SARS-CoV-2 wild-type strain was isolated from patients,^50^ while SARS-CoV-2 B.1.351 (Beta), SARS-CoV-2 B.1.617.2 (Delta), and SARS-CoV-2 XBB 1.16 variants came from the National Virus Resource Center. SARS-CoV-2 and its variants were conducted in the Biosafety Level 3 (BSL3) Laboratory at the Wuhan Institute of Virology, Chinese Academy of Sciences (CAS).

### Plasmids

Plasmids used in this study, including pcDNA3.1-SARS-CoV-2-S, pAAV-IRES-GFP-SARS-CoV-2-S, pcDNA3.1-SARS-CoV-S, pcDNA3.1-MERS-CoV-S, pcDNA3.1-HCoV-229E-S, pcDNA3.1-VSV-G, and pNL4-3.luc.RE were all kept in our laboratory.

### Cloning and expression of human CTSL/B

(CTSL_HUMAN aa18-333 and CTSB_HUMAN aa18-333) were ordered from GenScript and cloned into pET28 vector via the NdeI and BamH I sites. Expression in *Escherichia coli* (*E. coli*) BL21 (DE3) at 37 °C was induced by addition of IPTG (isopropyl β-*D*-1-thiogalactopyranoside). The method of cloning and producing authentic CTSL/B was referenced from the protocol published previously.^51–53^ Detailed experimental procedures are listed below.

### Isolation, solubilization, and folding of human CTSL/B inclusion bodies

Cell pellets were resuspended in 50 mM Tris/HCl, pH 8.0, containing 2 mM EDTA, 5% saccharose, and 2 mM Protease Inhibitor Cocktail. After cell disruption and centrifugation, IBs were washed with 50 mM Tris/HCl, pH 8.0, 2 mM EDTA, and 0.1% Triton X-100 and then centrifuged twice. The washing procedure was repeated twice with a second buffer of 50 mM Tris/HCl, pH 8.0, and 1 M urea. The final pellet was solubilized in 50 mM Tris/HCl (35 mL), pH 8.0, 5 mM EDTA, 8 M urea, 0.15 M NaCl, and 5 mM DTT and then centrifuged at 4 °C.

### Purification and activation of human CTSL

IB solution was diluted to a concentration of 2.5 mg/mL using a sample pump with a flow rate of 0.2 mL/min in 2 L refolding buffer (50 mM Tris/HCl buffer, pH 8.5, 0.5 M *L*-arginine, 0.01% BRIJ 35, 0.01 M NaCl, 200 μL Catalase (200,000 unit/g), 10 mM GSH, and 1 mM GSSG) at 20 °C. The mixture was stirred for 24 h at 20 °C, filtered, concentrated to 200 mL, and dialyzed at 4 °C overnight against 25 mM sodium phosphate buffer, pH 7.0, and 0.5 M NaCl. The protein solution was brought to a final concentration of 1.2 M (NH_4_)_2_SO_4_ and centrifuged. The supernatant was loaded on an HIC Phenyl Beads 6FF column (High Sub) equilibrated with 10 mM Tris/HCl, pH 8.0, 1 M NaCl, and 1.2 M (NH_4_)_2_SO_4_ and then eluted with a linear gradient to 50 mM Tris/HCl, pH 8.0, buffer. Fractions containing CTSL were pooled and incubated for 1 h at 37 °C in 100 ng/mL sodium dextran sulfate, 100 mM sodium acetate, pH 5.0, and 1 mM DTT to auto-activate the enzyme. The pooled fractions were concentrated and loaded on a Superdex 300 (XK) column equilibrated with 20 mM Bis-Tris/HCl, pH 7.0, 0.3 M NaCl, 5 mM DTT, and 2.5 mM EDTA.

### Purification and activation of human CTSB

IB solution was diluted to a concentration of 2.5 mg/mL by using a sample pump with a flow rate of 0.2 mL/min in 2 L 100 mM Tris/HCl buffer, pH 8.5, 0.5 M *L*-arginine, 0.01% BRIJ 35, and 5 mM *L*-cysteine at 20 °C. The mixture was stirred for 24 h at 20 °C, filtered, concentrated to 200 mL, and dialyzed at 4 °C overnight against 25 mM sodium phosphate buffer, pH 7.0, and 0.5 M NaCl. Fractions containing CTSL were pooled and incubated for 1 h at 37 °C in 100 ng/mL sodium dextran sulfate, 100 mM sodium acetate, pH 5.0, and 1 mM DTT to auto-activate the enzyme. The pooled fractions were concentrated and loaded on a HiLoad 16/600 Superdex 200 pg column equilibrated with 20 mM Bis-Tris/HCl methane, pH 7.0, 0.3 M NaCl, 5 mM DTT, and 2.5 mM EDTA.

### Cloning and expression of human CAPN1 protease core (aa29-360)

The full-length gene that encodes CAPN1_HUMAN was optimized and synthesized for *E. coli* expression (GenScript). The CAPN1 protease core gene sequence was amplified by PCR and inserted into a modified pET28a vector via In-Fusion Cloning to produce the expression vector pET28a-CAPN1_29–360_, which contained a hexa-histidine tag and a human rhinovirus 3C protease site at the N-terminal of CAPN1 protease core. The expression plasmid and pGro7 were transformed into *E. coli* BL21 (DE3) cells and then cultured in Luria broth medium containing 50 μg/mL Kanamycin, 25 μg/mL Chloramphenicol, and 1 mg/mL *L*-arabinose at 37 °C. When the cells were grown to an optical density at 600 nm of 0.6–0.8, 25 μM IPTG was added to the cell culture to induce expression at 16 °C. After 16 h, cells were collected by centrifugation at 3000 *g*.

### Purification of CAPN1 protease core (aa29-360)

Cell pellets were resuspended in lysis buffer (50 mM Tris/HCl, pH 8.0, 150 mM NaCl, and 5 mM β-mercaptoethanol), lysed by high-pressure homogenization at 4 °C, and then centrifuged at 40,000 *g* for 30 min. The supernatant was loaded onto a Ni-NTA affinity column (GenScript) and washed by lysis buffer containing 20 mM imidazole. His-tagged CAPN1_29-360_ was eluted by lysis buffer containing 300 mM imidazole. Human rhinovirus 3 C protease was added to remove the N-terminal His tag. The protein was further purified by ion-exchange chromatography and size-exclusion chromatography. The purified protein was stored in 10 mM Tris/HCl, pH 8.0, 100 mM NaCl, 2 mM CaCl_2_, and 5 mM β-mercaptoethanol at −80 °C until needed.

### Cloning expression and purification of SARS-CoV-2 M^pro^

Cloning expression and purification of SARS-CoV-2 M^pro^ were set up according to the protocols published previously.^41–43^ The cell pellets were resuspended in lysis buffer (20 mM Tris/HCl, pH 8.0, 150 mM NaCl, 5% (v/v) Glycerol), lysed by high-pressure homogenization at 4 °C, and then centrifuged at 40,000 *g* for 30 min. The supernatant was loaded onto the Ni-NTA affinity column (GenScript) and washed by lysis buffer containing 20 mM imidazole. His-tagged M^pro^ was eluted by lysis buffer containing 300 mM imidazole. The imidazole was then removed through desalting. Human rhinovirus 3C protease was added to remove the C-terminal His tag. SARS-CoV-2 M^pro^ was further purified by ion-exchange chromatography. The purified M^pro^ was stored in 10 mM Tris/HCl, pH 8.0, at −80 °C until needed.

### Enzymatic activity and inhibition assays of human CTSL/B

Enzymatic activity and inhibition assays were set up according to the protocols published previously.^51–53^ Recombinant CTSL/B (2 nM at a final concentration) was mixed with serial dilutions of each compound in 50 µL assay buffer (100 mM potassium phosphate, pH 6.5, 5 mM EDTA-Na, 0.001% Triton X-100, and 5 mM DDT) and incubated for 5 min. The reaction was initiated by adding 4 µL fluorogenic substrate (Z-VVR-AMC for CTSL and Z-Arg-Arg-AMC for CTSB; AMC = 7-amino-4-methyl-coumarin, and *Z* = (benzyloxy)carbonyl) with a final concentration of 20 µM. After that, the fluorescence signal at 360 nm (excitation)/450 nm (emission) was immediately measured every 30 s for 10 min with a Bio-Tek Synergy4 plate reader. The *V*_max_ of reactions added with compounds at various concentrations compared to the reaction added with DMSO were calculated and used to generate IC_50_ curves. For each compound, three independent experiments were performed. IC_50_ values against CTSL/B were computed based on inhibitory rates at 11 different concentrations.

### Enzymatic activity and inhibition assays of human CAPN1

Human CAPN1 was purchased from Merck (208713-500UG, Human erythrocytes). Compounds were incubated with 8.24 μg/mL enzyme, 0.05% casein-FITC, and 100 μM CaCl_2_ in Tris/HCl, pH 7.4, for 30 min at 37 °C. The reaction was then terminated by the addition of 20% trichloroacetic acid. Then 1.5 equivalent 0.5 M disodium hydrogen phosphate was added to neutralize. After that, the fluorescence signal at 485 nm (excitation)/535 nm (emission) was immediately measured. For each compound, IC_50_ values against CAPN1 were measured at eight different concentrations, and an experiment was performed.

### Enzymatic activity and inhibition assays of SARS-CoV-2 M^pro^

Enzymatic activity and inhibition assays were set up according to the protocols published previously.^41–43^ The activity of SARS-CoV-2 M^pro^ was measured by a FRET-based cleavage assay with the substrate Mca-AVLQ ↓ SGFR-K(Dnp)K (GL Biochem). The fluorescence signal was monitored at an emission wavelength of 405 nm with excitation at 340 nm using an EnVision multimode plate reader (Perkin Elmer). Purified M^pro^ (100 nM at a final concentration) was mixed with serial dilutions of each compound in 54 µL assay buffer (50 mM Tris/HCl, pH 7.4, and 1 mM EDTA). The reaction was initiated by adding 5 µL substrate with a final concentration of 20 µM. A detergent-based control was performed by adding 0.01% (v/v) Triton X-100 to exclude the possibility of inhibitor-induced aggregation. For each compound, three independent experiments were performed. IC_50_ values against SARS-CoV-2 M^pro^ were computed based on inhibitory rates at 13 different concentrations.

Enzymatic activity and inhibition assays of both compounds against the remaining proteases in Supplementary Table S2 were measured by a commercial company.

### Crystallization

Human CTSL (8 mg/mL) was incubated with compounds in a 1:5 molar ratio for 3 h at room temperature. The crystals of CTSL in complex with **14a** and **14b** were obtained after 2–5 days by hanging drop vapor diffusion. Droplets were set up at 16 °C by mixing 1 µL protein solution with 2 µL reservoir solution consisting of 100 mM sodium acetate, pH 4.1, and 15% (w/v) PEG 2000. The cryoprotectant solution was the reservoir, but with 20% glycerol added.

Human CAPN1 protease core (13 mg/mL) was incubated with 10 mM compounds for 1 h at room temperature, and the complex was crystallized using the hanging drop vapor diffusion method at 20 °C. The crystals of CAPN1 protease core in complex with **14a** grew when the complex was mixed with the reservoir solution containing 25% (w/v) PEG 4000, 0.1 M sodium/potassium phosphate (pH 6.5), and 0.2 M sodium chloride in a 1:1 volume ratio. The crystals of CAPN1 protease core in complex with **14b** grew when the complex was mixed with the reservoir solution containing 7% (w/v) PEG 4000 and 0.1 M sodium acetate (pH 4.9) in a 1:2 volume ratio. The cryoprotectant solution was the reservoir, but with 20% (v/v) glycerol added.

SARS-CoV-2 M^pro^ (6 mg/mL) was incubated with 1 mM compounds for 1 h at room temperature, and the complex was crystallized using the hanging drop vapor diffusion method at 20 °C. Diffracting crystals leading to the structure grew when the complex was mixed with the reservoir solution containing 7% (w/v) PEG 6000, 0.1 M MES (pH 5.4–5.8), and 6% (v/v) DMSO in a 1:1 volume ratio. The cryoprotectant solution was the reservoir, but with 20% (v/v) glycerol added.

### Data collection and structure determination

X-ray data were collected on beamline BL02U1 and BL19U1 at Shanghai Synchrotron Radiation Facility at 100 K using an EIGER2 S 9M image plate detector or Pilatus3 6 M image plate detector. Data integration and scaling were performed using the XDS program.^54^ The structure was determined by molecular replacement (MR) with PHASER.^55^ and Phenix 1.19.2.^56^ using human CTSL (PDB ID: 6F06),^57^ human CAPN1 protease core (PDB ID: 1ZCM),^58^ and SARS-CoV-2 M^pro^ (PDB ID: 6LU7),^42^ as a search template, respectively. The model from MR was subsequently subjected to iterative cycles of manual model adjustment with Coot 0.8.9.2,^59^ and refinement was completed with Phenix REFINE.^60^ The inhibitors were built according to the omit map. The phasing and refinement statistics are summarized in Supplementary Table S3.

### Pseudovirus inhibition assay

Pseudoviruses, including SARS-CoV-2 PsV, SARS-CoV PsV, MERS-CoV PsV, HCoV-229E PsV, and VSV PsV, were constructed as previously described.^44,45^ Briefly, 293 T/ACE2 cells were co-transfected with a plasmid encoding S protein of the above-described CoV, e.g., pcDNA3.1-SARS-CoV-2-S and pNL4–3.luc.RE, using VigoFect (Vigorous Biotechnology, Beijing, China). The supernatants containing the released pseudovirus particles were harvested at 72 h post-transfection, centrifuged at 3,000 g for 10 min, and stored at −80 °C until use. PsV inhibition assay was performed as previously reported.^44,45^ Target cells (293T/ACE2 cells for SARS-CoV-2 PsV and Huh-7 cells for other PsVs) were digested and seeded in wells of a 96-well plate 12 h before PsV inhibition assay. Diluted compounds were co-incubated with PsV supernatants for 30 min at 37 °C and added into target cells. Media were changed for fresh DMEM containing 5% FBS after 12 h, and luciferase activity was measured after 36 h.

### Authentic virus inhibition assay

Authentic virus inhibition assays were performed as previously reported.^44,45^ Authentic HCoV-OC43 and HCoV-229E inhibition assays were performed in a biosafety level 2 (BSL2) laboratory, and diluted compounds were co-incubated with authentic virus solutions for 30 min at 37 °C. Afterwards, these compounds-authentic virus mixtures were added into target cells cultured in wells of a 96-well plate, including Huh-7 cells for HCoV-229E and RD cells for HCoV-OC43. Three to five days later, Cell Counting Kit-8 (CCK8) solutions were added to measure virus-induced cytopathy, and EC_50_ values were calculated. Inhibition assays for authentic SARS-CoV-2 wild-type strain and its variants, Vero E6 cells or 293T/ACE2-TMPRSS2 cells were maintained in DMEM supplemented with 10% FBS at 37 °C and humidified 5% CO_2_. Before infection, target cells were seeded into 48-well plates in DMEM (10% FBS) and incubated at 37 °C and humidified 5% CO_2_. After 12 h, the medium was replaced with 200 μL of DMEM (2% FBS) per well containing the compound at 10 μM (for primary screen) or one concentration within six gradients (for EC_50_ determination) to incubate for 2 h, then SARS-CoV-2 or its variants were added at an MOI of 0.01 and then plates were incubated at 37 °C and humidified 5% CO_2_. At 24 h post-infection, the supernatants were collected and the viral RNA in supernatants was extracted. SARS-CoV-2 wild-type strain and its variants, B.1.351 (Beta) and 1.617.2 (Delta) were performed in a BSL3 laboratory. Real-time quantitative PCR (RT-qPCR) assay was used to determine the viral load of SARS-CoV-2 and its variants, and EC_50_ values were calculated.

### In vitro toxicity study

Cell viability was performed in a 96-well plate with triplicate for each concentration. All compounds were diluted two times with nine gradients starting at 200, 250. or 500 micromoles in maintenance medium. After 48 h incubation, the supernatant was removed, and 10 μL WST-8 (2-(2-methoxy-4-nitrophenyl)-3-(4-nitrophenyl)-5-(2,4-disulfophenyl)-2*H*-tetrazolium sodium salt) in maintenance medium was added to the cells. After incubation at 37 °C for 2 h, the plates were measured at 450 nm using a spectrophotometer (BioTek), and cell viability was calculated. All compounds were diluted with maintenance medium containing 2%FBS of DMEM.

### Time-of-addition assay

Time-of-addition assay was performed as previously reported.^46^ 50 μL SARS-CoV-2 PsV supernatants were added into Huh-7 (or 293T/ACE2) cells cultured in wells of a 96-well plate. Afterwards, compounds were added at 0, 1, 2, 3, 4, 5, 6, and 8 h post-SARS-CoV-2 PsV addition (with the final concentrations of 500 nM). Luciferase activity was measured after 36 h.

### SARS-CoV-2-S-mediated cell-cell fusion

Cell-cell fusion assay was performed as previously reported.^47^ The 293T/ACE2 cell line was transfected with plasmid pAAV-IRES-GFP-SARS-CoV-2-S for the co-expression of SARS-CoV-2 spike protein and GFP (effector cells). Huh-7 cells were used as target cells. Effector cells were first mixed with diluted compounds at 37 °C for 30 min, and this mixture was added into Huh-7 cells cultured in wells of a 96-well plate for fusion. Fused/unfused cells were then counted.

### Evaluation of in vivo antiviral efficacy of **14a** and **14b** in SARS-CoV-2-infected K18-hACE2 transgenic mouse model

K18-hACE2 male mice at 7–8 weeks of age were purchased from Jiangsu GemPharmatech Biotechnology Co., Ltd. (Jiangsu, China). Animal experiments conformed to the guidelines governing the use and care of laboratory animals and were approved by the Institutional Review Board of the Wuhan Institute of Virology, CAS. Viral infections were performed in a biosafety level 3 (BSL-3) facility. Animals were divided into five groups (*n* = 3 for each group), including the vehicle group, the two groups receiving **14a** (10 mg/kg and 20 mg/kg, **14a**-10 mg/kg and **14a**-20 mg/kg for short, respectively), and the two groups receiving **14b** (10 mg/kg and 20 mg/kg, **14b**-10 mg/kg and **14b**-20 mg/kg for short, respectively). Compounds **14a** and **14b** were formulated in a vehicle consisting of 5% dimethyl sulfoxide, 5% ethanol, 10% polyethylene glycol-15-hydroxystearate (Kolliphor HS15) and 80% saline (volume ratio). After 1 h of compounds or vehicle intranasally treating, the mice were intranasally challenged by SARS-CoV-2 XBB 1.16 (1 × 10^4^ PFU per mouse, designated as day 0). Then, mice were administrated once at 24 h post infection (hpi, day 1). On day 2, the mice were euthanized to take lung tissues. Lung tissues were collected for the detection of viral RNA.

### Protective effect assessment of **14b** against HCoV-OC43 in vivo

Protective effect of **14b** against HCoV-OC43 in vivo was assessed as previously reported.^45^ All newborn mice were challenged with HCoV-OC43 (100 TCID_50_) by intranasal administration. For mice in the **14b**-treated group, **14b** (5 mg/kg) in a proper solution (1% dimethyl sulfoxide, 9% polyethylene glycol 300, and 90% aqueous solution containing 28% (w/v) hydroxypropyl β-cyclodextrin, volume ratio) was given by intraperitoneal injection. Mice in the viral control group were treated with PBS. Body weight and survival rate of these mice were then observed up to the 15th day post-infection.

### Anti-inflammatory assay for **14a** and **14b** in vitro

Mouse macrophage Raw 264.7 cells were incubated with compounds **14a** or **14b** at different doses for 2 h. Then, the cells were stimulated with LPS (200 ng/mL) for 6 h, followed by RT-qPCR analysis.

RNA isolation, cDNA synthesis, and RT-qPCR. Total RNA was isolated from cells using RNA extraction reagent (Vazyme, R401-01). cDNA was synthesized using the HiScript IIQ RT SuperMix (Vazyme, R223-01), according to manufacturer’s instructions. RT-qPCR was performed using ChamQ SYBR qPCR Master Mix (Vazyme, Q331-02) in a CFX96TM Real-Time PCR Detection System (Bio-Rad, Shanghai, China). The profile of thermal cycling consisted of initial denaturation at 95 °C for 30 s, 40 cycles at 95 °C for 5 s and 60 °C for 30 s. All primer sequences used in this study are listed in Supplementary Table S5.

### Anti-inflammatory effects of **14a** and **14b** in vivo

C57BL/6 mice were obtained from Beijing HFK Bioscience Co., Ltd. (Beijing, China). Animals 6–8 weeks of age were used for the studies and were maintained with free access to pellet food and water in plastic cages at 21 ± 2 °C and kept on a 12 h light/dark cycle. Mice were randomly divided into four groups, each group containing eight mice. The mice were intraperitoneally administered with 100 mg/kg of **14a** or 100 mg/kg of **14b** or vehicle control 1 h prior to LPS challenge. The compounds were dissolved in solvent containing 5% dimethyl sulfoxide, 5% ethanol, 40% polyethylene glycol 400 and 50% saline (volume ratio). The mice were anesthetized by Zoletil 50. Then mice in the normal group were intranasally inoculated with 20 μL normal saline, and mice in the LPS challenge group were intranasally inoculated with 20 μL (5 μg/μL) LPS to induce pneumonia. At 12 h post-induction, the mice were again intraperitoneally administered with 100 mg/kg of **14a** or 100 mg/kg of **14b** or vehicle control. Twenty-four hours later, mice were euthanized, and the lung tissues were harvested for further study.

RNA isolation, cDNA synthesis, and RT-qPCR. Total RNA was isolated from lung tissues using RNA extraction reagent (Vazyme, R401-01). cDNA was synthesized using the HiScript II Q RT SuperMix (Vazyme, R223-01), according to the manufacturer’s instructions. RT-qPCR was performed using ChamQ SYBR qPCR Master Mix (Vazyme, Q331-02) in the CFX96TM Real Time PCR Detection System (Bio-Rad, Shanghai, China). The profile of thermal cycling consisted of initial denaturation at 95 °C for 30 s, 40 cycles at 95 °C for 5 s and 60 °C for 30 s. All primer sequences used in this study are listed in Supplementary Table S5.

Hematoxylin and eosin (H&E) staining. Lung tissue samples were fixed in 4% paraformaldehyde and embedded in paraffin (Thermo Scientific, 8331). Colon was sectioned (5 μm) for H&E staining, and the stained sections were analyzed by a pathologist using a light microscope (Olympus, Tokyo, Japan). Lung injury was evaluated based on histological features, including alveolar septal thickening and inflammatory cell infiltration.

### In vivo toxicity study in mice

C57BL/6 mice (aged 6–8 weeks, 16–25 g) consisting of half male and half female were used to assess toxicity of compounds **14a** and **14b** in vivo. Compounds, vehicle, dosage, and administration schemes are shown in Supplementary Table S6. In short, mice were treated with compounds **14a** and **14b** at the indicated doses by oral gavage and the mice were clinically observed for 12 days after administration, including body weights, food consumption, and general status. At the end of the studies, the mice were anesthetized by Zoletil 50, and the organ and tissue samples were observed by naked eyes.

### Quantification and statistical analysis

All experimental data from enzymatic inhibitory and antiviral assays were analyzed using GraphPad Prism 8.0 software.

### Supplementary information

Final_Manuscript_Supplementary_Materials

CAPN1_14a

CAPN1_14b

CTSL_14a

CTSL_14b

Mpro_14a

Mpro_14b

## Data Availability

All data are available in the manuscript or the supplementary material. The X-ray crystal structures of human CTSL in complex with **14a** and **14b**, human CAPN1 protease core in complex with **14a** and **14b**, and SARS-CoV-2 M^pro^ in complex with **14a** and **14b**, have been deposited in the Protein Data Bank, under accession codes 7W33, 7W34, 7W7O, 7X79, 8GXG and 8GXH, respectively.
