# Supplementary material for: Structure-based design of pan-coronavirus inhibitors targeting host cathepsin L and calpain-1
Source: Signal Transduct Target Ther. 2024 Mar 6;9:54. doi: 10.1038/s41392-024-01758-8 (PMC10914734; doi:10.1038/s41392-024-01758-8)
Supplement: Supplementary file 1 — Final_Manuscript_Supplementary_Materials [file 41392_2024_1758_MOESM1_ESM.docx]

Supplementary Materials for

**Structure-based design of pan-coronavirus inhibitors targeting host cathepsin L and calpain-1**

Xiong Xie^1,2^, Qiaoshuai Lan^3^, Jinyi Zhao^2,4^, Sulin Zhang^1,2^, Lu Liu^1,2,4^, Yumin Zhang^5^, Wei Xu^3^, Maolin Shao^2,4^, Jingjing Peng^1,2^, Shuai Xia^3^, Yan Zhu^4^, Keke Zhang^1,6^, Xianglei Zhang^4^, Ruxue Zhang^2,5^, Jian Li^1,6^, Wenhao Dai^1,2^, Zhen Ge^6^, Shulei Hu^1^, Changyue Yu^1,2^, Jiang Wang^1,2^, Dakota Ma^1^, Mingyue Zheng^1,2,6,7^, Haitao Yang^4^, Gengfu Xiao^2,5^, Zihe Rao^4^, Lu Lu^3^, Leike Zhang^2,5*^, Fang Bai^4*^, Yao Zhao^8*^, Shibo Jiang^3*^, Hong Liu^1,2,4,6,7*^

Correspondence to: hliu@simm.ac.cn, shibojiang@fudan.edu.cn, zhaoyao@shanghaitech.edu.cn, baifang@shanghaitech.edu.cn, zhangleike@wh.iov.cn

**This PDF file includes:**

Supplementary Text

Figures S1 to S8

Tables S1 to S7

Schemes S1 to S5

**Supplementary Text**

**Progress on host CTSL and CAPN1 inhibitors**


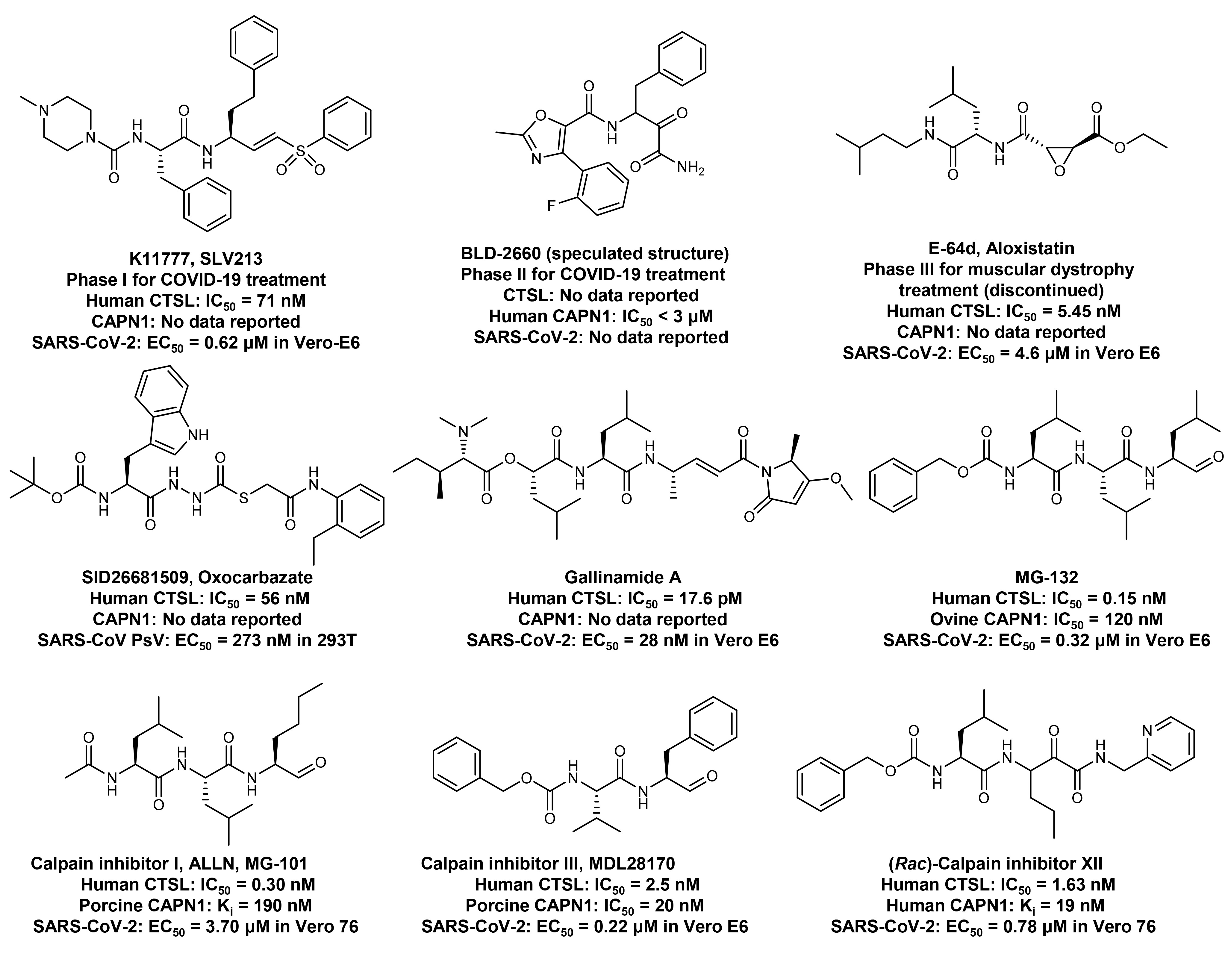


**Scheme S1 The representative human CTSL and CAPN1 peptidomimetic inhibitors with inhibitory activity against coronaviruses.**

**General procedures for preparation of compounds**

General procedures for preparation of compounds: Reagents or chemicals were obtained from commercial sources and used without further purification. All reactions were monitored by analytical thin layer chromatography (HSGF 254, 0.15-0.2 mm thickness). All products were purified by column chromatography with 300-400 mesh silica gel. Compounds were characterized by NMR and MS. ^1^H, ^13^C and ^19^F NMR were operated on a 400 MHz (AVANCE III 400), 500 MHz (AVANCE III 500) or 600 MHz (AVANCE III 600) instrument. High-resolution mass spectra (HRMS) were recorded on a UHPLC-QTOF spectrometer. The purity of target products was determined by HPLC.

**Synthesis of compounds 14a, 14b, 14l and 14m**

**Scheme S2** Reagents and conditions: **a**, 3-bromopropionitrile, LiHMDS, THF, -78 °C, 20 h; **b**, NaBH_4_, CoCl_2_·6H_2_O, MeOH, 0 °C - r.t., 12 h; **c**, 4 M HCl in 1,4-dioxane, r.t., 2 h; **d**, HATU, DIPEA, DCM, -20 °C, 12 h; **e**, 4 M HCl in 1,4-dioxane, r.t., 2 h; **f**, HATU, DIPEA, DCM, -20 °C, 12 h; **g**, NaBH_4_, THF/MeOH = 10:1 (v/v), 0 °C - r.t., 4 h; **h**, Dess-Martin periodinane, DCM, r.t., 2 h; **i**, Benzyl isocyanide, AcOH, DCM, r.t., overnight; **j**, LiOH·H_2_O, MeOH/H_2_O = 3:1 (v/v), r.t., 30 min; **k**, Dess-Martin periodinane, DCM, r.t., 2 h.

*Dimethyl (2S,4S)-2-((tert-butoxycarbonyl)amino)-4-(2-cyanoethyl)pentanedioate* ***(2)***

To a stirred solution of N-Boc-L-glutamic acid dimethyl ester **1** (14.0 g, 50.8 mmol) in anhydrous tetrahydrofuran (THF) (120 mL) under Argon was added dropwise a solution of lithium bis(trimethylsilyl)amide (LiHMDS) (107 mL, 1 M in THF) at -78 °C. The reaction mixture was stirred at -78 °C for 1 h. Then a solution of 3-bromopropionitrile (7.15 g, 53.4 mmol) in anhydrous THF (40 mL) was added dropwise, and the mixture was stirred at -78 °C for another 4 h. Subsequently, the reaction mixture was quenched by the addition of saturated NH_4_Cl solution and warmed to room temperature (r.t.). The reaction mixture was extracted with ethyl acetate (EA) (100 mL × 3), and the combined organic phase was washed with brine (200 mL × 3), dried over Na_2_SO_4_ and concentrated under vacuum. The residue was purified by flash column chromatography (petroleum ether (PE)/EA = 90:10 to 70:30, v/v) to give colorless oil product **2** (8.67 g, 52%). MS (ESI) *m/z* 229.1 [M – Boc + H]^+^. ^1^H NMR (500 MHz, CDCl_3_) *δ* 5.07 (d, *J* = 7.9 Hz, 1H), 4.46 – 4.32 (m, 1H), 3.75 (s, 3H), 3.72 (s, 3H), 2.63 (dt, *J* = 14.0, 7.0 Hz, 1H), 2.45 – 2.34 (m, 2H), 2.09 – 1.95 (m, 4H), 1.45 (s, 9H).

*Methyl (S)-2-((tert-butoxycarbonyl)amino)-3-((S)-2-oxopiperidin-3-yl)propanoate* ***(3)***

To a solution of intermediate **2** (8.00 g, 24.4 mmol) in dry methanol (MeOH) (100 mL) was added CoCl_2_·6H_2_O (3.48 g, 14.6 mmol) at 0 °C in one portion, and then NaBH_4_ (5.53 g, 146.2 mmol) was added in portions. After stirring for 1 h at 0 °C, the reaction mixture was warmed to room temperature and stirred for 12 h. Subsequently, the reaction was quenched by saturated NH_4_Cl solution, and the mixture was filtered through a Celite pad. The filtrate was then concentrated to remove volatile solvent. The residue was extracted with EA (80 mL × 3), and the combined organic layer was washed with brine (150 mL) and dried over Na_2_SO_4_, followed by removal of EA in vacuum. The crude product was further purified by flash column chromatography (PE/EA = 1:1 to 1:4, v/v) to give compound **3** (3.12 g, 43%) as a white solid. MS (ESI) *m/z* 201.1 [M – Boc + H]^+^. ^1^H NMR (500 MHz, CDCl_3_) *δ* 6.35 (s, 1H), 5.66 (d, *J* = 8.3 Hz, 1H), 4.36 – 4.29 (m, 1H), 3.73 (s, 3H), 3.35 – 3.27 (m, 2H), 2.42 – 2.33 (m, 1H), 2.29 (m, 1H), 2.19 – 2.11 (m, 1H), 1.95 – 1.81 (m, 2H), 1.80 – 1.70 (m, 1H), 1.61 – 1.52 (m, 1H), 1.44 (s, 9H).

*Methyl (S)-2-amino-3-((S)-2-oxopiperidin-3-yl)propanoate hydrochloride* ***(4)***

Compound **3** (1.00 g, 3.33 mmol) was dissolved in dichloromethane (DCM) (10 mL). Then a solution of hydrochloric acid (HCl) in 1,4-dioxane (4 M, 10 mL) was added, and the mixture was stirred at room temperature for 4 h. The volatile solvent was removed under vacuum to get crude product **4** as a white solid for direct use in the next step without further purification.

*Methyl (S)-2-((S)-2-((tert-butoxycarbonyl)amino)-3-cyclohexylpropanamido)-3-((S)-2-oxopiperidin-3-yl)propanoate* ***(6b)***

2-(7-Azabenzotriazol-1-yl)-N,N,N',N'-tetramethyluronium hexafluorophosphate (HATU) (1.87 g, 4.92 mmol) was added into a stirred solution of Boc-*L*-Cha-OH **5b** (0.89 g, 3.28 mmol) in DCM (50 mL), which was cooled to -20 °C, and the mixture was stirred at -20 °C for 30 min. Subsequently, the crude intermediate **4** and N,N-diisopropylethylamine (DIPEA) (1.27 g, 9.84 mmol) were added sequentially, and the reaction mixture was stirred at -20 °C for 12 h. After the starting materials were consumed, the mixture was washed sequentially with saturated NH_4_Cl solution (50 mL×3), saturated NaHCO_3_ solution (50 mL×3), and brine (50 mL×3) and then dried over Na_2_SO_4_. The mixture was evaporated to afford the crude product, which was further purified by flash column chromatography (DCM/MeOH = 50:1 to 30:1, v/v) to give compound **6b** (1.28 g, 86%) as a white solid. MS (ESI) *m/z* 454.0 [M + H]^+^. ^1^H NMR (400 MHz, DMSO-*d_6_*) *δ* 8.31 (d, *J* = 8.2 Hz, 1H), 7.43 (s, 1H), 6.84 (d, *J* = 7.9 Hz, 1H), 4.43 – 4.33 (m, 1H), 4.00 – 3.90 (m, 1H), 3.60 (s, 3H), 3.14 – 3.05 (m, 2H), 2.31 – 2.13 (m, 2H), 1.91 – 1.80 (m, 1H), 1.78 – 1.46 (m, 10H), 1.44 – 1.05 (m, 14H), 0.93 – 0.77 (m, 2H).

*Methyl (S)-2-((S)-2-amino-3-cyclohexylpropanamido)-3-((S)-2-oxopiperidin-3-yl)propanoate hydrochloride* ***(7b)***

To a solution of intermediate **6b** (1.00 g, 2.20 mmol) in DCM (10 mL) was added HCl (10 mL, 4M in 1,4-dioxane), and the mixture was stirred at ambient temperature for 4 h. Then the solvent was removed to get crude product **7b** as an off-white solid and used without further purification.

*Methyl (S)-2-((S)-2-(benzofuran-2-carboxamido)-3-cyclohexylpropanamido)-3-((S)-2-oxopiperidin-3-yl)propanoate* ***(9b)***

To a solution of benzofuran-2-carboxylic acid **8** (0.35 g, 2.16 mmol) in DCM (50 mL), which was cooled to -20 °C, HATU (1.23 g, 3.24 mmol) was added, and the resulting mixture was stirred at -20 °C for 30 min. Then crude intermediate **7b** and DIPEA (0.84 g, 6.48 mmol) were added, and the mixture was stirred at -20 °C for 12 h. The mixture was washed with saturated NH_4_Cl solution (50 mL×3), saturated NaHCO_3_ solution (50 mL×3), and brine (50 mL×3). The organic layer was dried over Na_2_SO_4_ and concentrated under reduced pressure. The residue was further purified by flash column chromatography (DCM/MeOH = 50:1 to 30:1, v/v) to obtain white solid **9b** (0.89 g, 83%). MS (ESI) *m/z* 497.9 [M + H]^+^. ^1^H NMR (400 MHz, DMSO-*d_6_*) *δ* 8.62 (dd, *J* = 8.2, 2.4 Hz, 2H), 7.78 (d, *J* = 7.8 Hz, 1H), 7.68 (d, *J* = 8.4, 1H), 7.63 (s, 1H), 7.51 – 7.42 (m, 2H), 7.37 – 7.29 (m, 1H), 4.61 – 4.51 (m, 1H), 4.44 – 4.34 (m, 1H), 3.61 (s, 3H), 3.13 – 3.02 (m, 2H), 2.31 – 2.18 (m, 2H), 1.88 – 1.78 (m, 1H), 1.77 – 1.46 (m, 10H), 1.42 – 1.27 (m, 2H), 1.26 – 1.04 (m, 3H), 0.98 – 0.82 (m, 2H).

*N-((S)-3-cyclohexyl-1-(((S)-1-hydroxy-3-((S)-2-oxopiperidin-3-yl)propan-2-yl)amino)-1-oxopropan-2-yl)benzofuran-2-carboxamide* ***(10b)***

A round-bottom flask was charged with compound **9b** (0.80 g, 1.61 mmol) and dry THF (30 mL), and then NaBH_4_ (0.36 g, 9.65 mmol) was added in one portion. Subsequently, MeOH (2 mL) was added dropwise, and the reaction mixture was stirred at room temperature for 1 h. After compound **9b** was consumed, excess saturated aqueous NH_4_Cl was added to quench any unreacted NaBH_4_. The mixture was extracted with EA (50 mL×3), and the organic phase was washed with saturated aqueous NH_4_Cl (50 mL×3) and brine (50 mL×1). The EA layer was dried over Na_2_SO_4_ and concentrated under vacuum. The residual solid was further purified by column chromatography (DCM/MeOH = 30:1 to 20:1, v/v) to get product **10b** (0.68 g, 90%) as a white solid. MS (ESI) *m/z* 470.0 [M + H]^+^. ^1^H NMR (600 MHz, DMSO-*d_6_*) *δ* 8.55 (d, *J* = 8.3 Hz, 1H), 7.82 (d, *J* = 8.9 Hz, 1H), 7.77 (dd, *J* = 7.7, 1.1 Hz, 1H), 7.68 (dd, *J* = 8.4, 1.0 Hz, 1H), 7.62 (d, *J* = 1.0 Hz, 1H), 7.48 – 7.44 (m, 1H), 7.36 – 7.31 (m, 2H), 4.67 (t, *J* = 5.7 Hz, 1H), 4.54 – 4.47 (m, 1H), 3.87 – 3.82 (m, 1H), 3.36 – 3.51 (m, 1H), 3.25 – 3.20 (m, 1H), 3.12 – 3.00 (m, 2H), 2.18 – 2.12 (m, 1H), 2.05 – 1.99 (m, 1H), 1.92 – 1.86 (m, 1H), 1.77 – 1.60 (m, 6H), 1.60 – 1.53 (m, 2H), 1.51 – 1.41 (m, 2H), 1.37 – 1.21 (m, 2H), 1.20 – 1.06 (m, 3H), 0.95 – 0.81 (m, 2H).

*N-((S)-3-cyclohexyl-1-oxo-1-(((S)-1-oxo-3-((S)-2-oxopiperidin-3-yl)propan-2-yl)amino)propan-2-yl)benzofuran-2-carboxamide* ***(11b)***

To a solution of compound **10b** (0.60 g, 1.28 mmol) in DCM (20 mL) was added Dess-Martin periodinane (DMP) (0.65 g, 1.53 mmol) slowly, and the reaction was stirred at ambient temperature. When the reaction was completed by TLC detection, the mixture was washed sequentially with saturated Na_2_S_2_O_3_ solution (50 mL×3), saturated NaHCO_3_ solution (50 mL×3) and brine (50 mL×1). The organic phase was dried over Na_2_SO_4_ and concentrated under vacuum. The residue was purified by column chromatography (DCM/MeOH = 20:1, v/v) to get product **11b** (0.47 g, 79%) as a white solid. MS (ESI) *m/z* 467.9 [M + H]^+^. ^1^H NMR (400 MHz, DMSO-*d_6_*) *δ* 9.41 (s, 1H), 8.72 (d, *J* = 8.0 Hz, 1H), 8.59 (d, *J* = 7.7 Hz, 1H), 7.78 (d, *J* = 7.8 Hz, 1H), 7.70 – 7.66 (m, 1H), 7.64 (d, *J* = 1.0 Hz, 1H), 7.50 – 7.43 (m, 2H), 7.38 – 7.30 (m, 1H), 4.63 – 4.53 (m, 1H), 4.27 – 4.20 (m, 1H), 3.14 – 3.02 (m, 2H), 2.26 – 2.16 (m, 1H), 2.15 – 2.06 (m, 1H), 1.92 – 1.83 (m, 1H), 1.79 – 1.44 (m, 10H), 1.43 – 1.27 (m, 2H), 1.26 – 1.04 (m, 3H), 0.99 – 0.81 (m, 2H).

*(3S)-3-((S)-2-(benzofuran-2-carboxamido)-3-cyclohexylpropanamido)-1-(benzylamino)-1-oxo-4-((S)-2-oxopiperidin-3-yl)butan-2-yl acetate* ***(12b)***

Acetic acid (77 mg, 1.28 mmol) and benzyl isocyanide (0.15 g, 1.28 mmol) were added sequentially to the solution of compound **11b** (0.40 g, 0.86 mmol) in DCM (10 mL), and the mixture was stirred at room temperature for 12 h. After that, the volatile solvent was removed in vacuum, and the residue was purified by column chromatography (DCM/MeOH = 20:1, v/v) to get product **12b** (0.48 g, 87%) as an off-white solid. Compound **12b** is a mixture of two diastereomers, and racemization occurred at the newly formed stereocenters. MS (ESI) *m/z* 644.8 [M + H]^+^.

*N-((2S)-1-(((2S)-4-(benzylamino)-3-hydroxy-4-oxo-1-((S)-2-oxopiperidin-3-yl)butan-2-yl)amino)-3-cyclohexyl-1-oxopropan-2-yl)benzofuran-2-carboxamide* ***(13b)***

The diastereomers mixture **12b** (0.20 mg, 0.31 mmol) was dissolved in MeOH (9 mL) and water (3 mL), and then LiOH·H_2_O (20.0 mg, 0.46 mmol) was added. The mixture was stirred at room temperature for 30 min. Subsequently, EA (50 mL) was added and washed with brine (25 mL×3). The organic layer was dried over Na_2_SO_4_ and concentrated. The residual off-white solid was purified by column chromatography (DCM/MeOH = 20:1, v/v) to yield a pair of diastereomers, **13b** (0.17 g, 91%), as a white solid. MS (ESI) *m/z* 602.9 [M + H]^+^.

*N-((S)-1-(((S)-4-(benzylamino)-3,4-dioxo-1-((S)-2-oxopiperidin-3-yl)butan-2-yl)amino)-3-cyclohexyl-1-oxopropan-2-yl)benzofuran-2-carboxamide (****14b****)*

To a solution of the racemic mixture of compound **13b** (0.15 g, 0.25 mmol) in DCM (15 mL) was added DMP (0.16 g, 0.37 mmol), and the mixture was stirred at ambient temperature for 2 h. The mixture was washed with saturated Na_2_S_2_O_3_ solution (20 mL×3), saturated NaHCO_3_ solution (20 mL×3) and brine (20 mL×1). The organic layer was dried over Na_2_SO_4_ and concentrated under vacuum. The residue was purified by column chromatography (DCM/Acetone = 5:1 to 3:1, v/v) to get product **14b** (0.12 g, 80%) as a white solid. MS (ESI) *m/z* 600.8 [M + H]^+^, HRMS (ESI) *m/z*: [M + H]^+^ calculated for C_34_H_41_N_4_O_6_, 601.3021, found 601.3008. HPLC purity: 95.54%. **14b** was determined by a Waters e2695/2998 with Welch Xitmate C18 (4.6 × 250 mm, 5 μm). **14b** was analyzed using a gradient elution composed of MeOH and 0.1% phosphoric acid (H_3_PO_4_) in water (1.0 mL/min, 40 °C, and 37 min) **(Supplementary Table S7)**, and peak areas were calculated at 210 nm. ^1^H NMR (500 MHz, DMSO-*d_6_*) *δ* 9.21 (t, *J* = 6.4 Hz, 1H), 8.62 (d, *J* = 7.4 Hz, 1H), 8.57 (d, *J* = 8.2 Hz, 1H), 7.78 (d, *J* = 7.8 Hz, 1H), 7.68 (d, *J* = 8.4 Hz, 1H), 7.63 (s, 1H), 7.50 – 7.42 (m, 2H), 7.37 – 7.27 (m, 3H), 7.26 – 7.19 (m, 3H), 5.15 – 5.08 (m, 1H), 4.65 – 4.57 (m, 1H), 4.37 – 4.25 (m, 2H), 3.15 – 3.03 (m, 2H), 2.34 – 2.25 (m, 1H), 2.19 – 2.10 (m, 1H), 1.96 – 1.85 (m, 1H), 1.79 – 1.49 (m, 10H), 1.44 – 1.31 (m, 2H), 1.21 – 1.06 (m, 3H), 0.97 – 0.82 (m, 2H). ^13^C NMR (125 MHz, DMSO-*d_6_*) *δ* 196.8, 172.6, 172.2, 160.9, 157.9, 154.2, 148.7, 138.5, 128.3, 128.2, 127.2, 127.1, 126.9, 123.7, 122.8, 111.8, 109.8, 51.5, 50.5, 42.0, 41.1, 39.0, 37.0, 33.6, 33.1, 31.8, 31.3, 26.1, 25.7, 25.6, 21.2.

*N-((S)-1-(((S)-4-(benzylamino)-3,4-dioxo-1-((S)-2-oxopiperidin-3-yl)butan-2-yl)amino)-3-(4-fluorophenyl)-1-oxopropan-2-yl)benzofuran-2-carboxamide (****14a****)*

The synthesis procedure is similar to that of **14b**. MS (ESI) *m/z* 613.2 [M + H]^+^, HRMS (ESI) *m/z*: [M + H]^+^ calculated for C_34_H_34_FN_4_O_6_, 613.2457, found 613.2447. HPLC purity: 98.84%. **14a** was determined by a Waters e2695/2998 with Welch Xitmate C18 (4.6 × 250 mm, 5 μm). **14a** was analyzed using a gradient elution composed of MeOH and 0.01 M sodium dihydrogen phosphate (NaH_2_PO_4_) in water (pH 6.0) (1.0 mL/min, 35 °C, and 30 min) **(****Supplementary Table S7)**, and peak areas were calculated at 274 nm. ^1^H NMR (500 MHz, DMSO-*d_6_*) *δ* 9.24 (t, *J* = 6.4 Hz, 1H), 8.77 (d, *J* = 7.5 Hz, 1H), 8.72 (d, *J* = 8.5 Hz, 1H), 7.76 (d, *J* = 7.8 Hz, 1H), 7.65 (d, *J* = 8.4 Hz, 1H), 7.56 (s, 1H), 7.48 – 7.43 (m, 2H), 7.41 – 7.35 (m, 2H), 7.35 – 7.24 (m, 5H), 7.24 – 7.19 (m, 1H), 7.11 – 7.04 (m, 2H), 5.20 – 5.13 (m, 1H), 4.83 – 4.76 (m, 1H), 4.39 – 4.27 (m, 2H), 3.23 – 2.96 (m, 4H), 2.33 – 2.25 (m, 1H), 2.22 – 2.14 (m, 1H), 1.96 – 1.89 (m, 1H), 1.77 – 1.69 (m, 2H), 1.61 – 1.51 (m, 1H), 1.46 – 1.37 (m, 1H). ^13^C NMR (125 MHz, DMSO-*d_6_*) *δ* 196.6, 172.4, 171.2, 160.9 (d, *J* = 240.3 Hz), 160.8, 157.9, 154.1, 148.5, 138.4, 134.0, 134.0, 130.9 (d, *J* = 8.1 Hz), 128.2, 127.2, 127.0, 126.8, 123.6, 122.7, 114.7 (d, *J* = 20.9 Hz), 111.8, 109.7, 54.0, 51.6, 42.0, 41.1, 37.0, 36.3, 31.4, 25.6, 21.1. ^19^F NMR (376 MHz, DMSO-*d_6_*) *δ* -116.63 – -116.87 (m).

*(1S,3aR,6aS)-2-(benzofuran-2-carbonyl)-N-((S)-4-(benzylamino)-3,4-dioxo-1-((S)-2-oxopiperidin-3-yl)butan-2-yl)octahydrocyclopenta[c]pyrrole-1-carboxamide (****14l****)*

The synthesis procedure is similar to that of **14b**. MS (ESI) *m/z* 585.6 [M + H]^+^, HRMS (ESI) *m/z*: [M + H]^+^ calculated for C_33_H_37_N_4_O_6_, 585.2708, found 585.2709. Rotamers apparent in ^1^H NMR and ^13^C NMR spectrum. ^1^H NMR (500 MHz, DMSO-*d*_6_) *δ* 9.27 – 9.17 (m, 1H), 8.80 and 8.66 (2 × d, *J* = 7.5 Hz, 1H), 7.77 and 75.7 (2 × d, *J* = 7.8 Hz, 1H), 7.73 – 7.66 (m, 1H), 7.54 and 7.46 (2 × s, 1H), 7.49 – 7.38 (m, 2H), 7.37 – 7.21 (m, 6H), 5.16 – 5.08 and 4.98 – 4.90 (2 × m, 1H), 4.94 and 4.45 (d, *J* = 3.7 Hz, 1H), 4.39 – 4.21 (m, 2H), 4.13 and 3.81 (2 × dd, *J* = 10.9, 8.0 Hz, 1H), 3.76 and 3.54 (2 × dd, *J* = 12.3, 4.4 Hz, 1H), 3.18 – 3.05 (m, 1H), 3.02 – 2.85 (m, 1H), 2.80 – 2.67 (m, 1H), 2.61 – 2.54 and 2.43 – 2.34 (2 × m, 1H), 2.20 – 2.06 (m, 1H), 2.03 – 1.32 (m, 11H), 1.22 – 1.12 and 1.00 – 0.90 (2 × m, 1H). ^13^C NMR (126 MHz, DMSO-*d*_6_) *δ* 196.9, 196.8, 172.7, 172.2, 171.6, 161.0, 160.8, 158.3, 157.6, 154.0, 149.0, 148.7, 138.5, 138.4, 128.2, 127.2, 126.9, 126.8, 126.5, 123.6, 122.6, 122.4, 111.8, 111.7, 111.6, 111.0, 66.6, 66.3, 54.3, 54.2, 51.6, 51.4, 50.4, 46.9, 42.7, 41.9, 41.1, 40.9, 36.9, 32.4, 31.8, 31.5, 31.4, 31.2, 25.5, 25.1, 24.8, 24.6, 21.1, 20.6.

*(1R,2S,5S)-3-(benzofuran-2-carbonyl)-N-((S)-4-(benzylamino)-3,4-dioxo-1-((S)-2-oxopiperidin-3-yl)butan-2-yl)-6,6-dimethyl-3-azabicyclo[3.1.0]hexane-2-carboxamide (****14m****)*

The synthesis procedure is similar to that of **14b**. MS (ESI) *m/z* 585.5 [M + H]^+^, HRMS (ESI) *m/z*: [M + H]^+^ calculated for C_33_H_37_N_4_O_6_, 585.2708, found 585.2708. Rotamers apparent in ^1^H NMR and ^13^C NMR spectrum.^1^H NMR (500 MHz, DMSO-*d*_6_) *δ* 9.28 – 9.21 (m, 1H), 8.93 and 8.78 (2 × d, *J* = 7.4 Hz, 1H), 7.77 and 7.55 (2 × d, *J* = 7.8 Hz, 1H), 7.70 (t, *J* = 9.3 Hz, 1H), 7.58 and 7.41 (2 × s, 1H), 7.50 – 7.19 (m, 8H), 5.18 – 5.12 and 4.97 – 4.90 (2 × m, 1H), 5.10 and 4.56 (2 × s, 1H), 4.39 – 4.21 (m, 2H), 4.17 and 3.76 (2 × dd, *J* = 11.1, 5.4 Hz, 1H), 3.95 and 3.65 (2 × d, *J* = 11.1 Hz, 1H), 3.17 – 3.05 (m, 1H), 3.00 – 2.85 (m, 1H), 2.42 – 2.33 and 1.98 – 1.91 (2 × m, 1H), 2.18 – 2.04 (m, 1H), 1.77 – 1.66 (m, 1H), 1.64 – 1.52 (m, 2H), 1.50 – 0.86 (m, 4H), 1.06 and 1.04 (2 × s, 3H), 1.00 and 0.92 (2 × s, 3H). ^13^C NMR (126 MHz, DMSO-*d*_6_) *δ* 196.9, 196.8, 172.6, 172.1, 171.6, 170.8, 160.9, 160.6, 157.7, 156.9, 154.1, 154.0, 148.8, 148.4, 138.4, 138.4, 128.2, 127.2, 126.9, 126.7 (126.71, 126.66), 123.6, 126.4, 123.7, 122.7, 122.4, 112.0, 111.8, 111.7, 111.2, 60.9, 60.5, 51.6, 51.4, 48.2, 48.2, 42.0, 41.9, 41.1, 40.9, 36.9, 36.7, 33.4, 31.4, 31.3, 30.0, 27.4, 26.0, 25.9, 25.5, 25.0, 24.0, 21.2, 20.6, 18.7, 18.6, 12.7, 12.6.

**Synthesis of compounds 14c and 14d**

**Scheme S3** Reagents and conditions: **a**, NaH, THF, 0 °C to r.t., 2 h.

*N-((S)-3-(4-fluorophenyl)-1-oxo-1-(((S,E)-1-((S)-2-oxopiperidin-3-yl)-4-(phenylsulfonyl)but-3-en-2-yl)amino)propan-2-yl)benzofuran-2-carboxamide (****14c****)*

To a stirred 0 ℃ solution of diethyl ((phenylsulfonyl)methyl)phosphonate **15** (91.4 mg, 0.31 mmol) in anhydrous THF (10 mL) under argon atmosphere was added in portions sodium hydride (60% dispersion in mineral oil, 12.5 mg, 0.31 mmol), and the resulting mixture was stirred at 0 ℃ for 30 min. Then a solution of intermediate **11a** (100 mg, 0.21 mmol) in 10 mL anhydrous THF was added, and the mixture was warmed to room temperature and reacted for 2 h. The reaction mixture was quenched by the addition of the saturated aqueous NH_4_Cl solution at 0 ℃. The mixture was added EA (50 mL), washed with saturated NaHCO_3_ solution (50 mL×3), and brine (50 mL×3). The organic layer was dried over Na_2_SO_4_ and concentrated under reduced pressure. The residue was purified by column chromatography (DCM/MeOH = 20:1, v/v) to get product **14c** (98.2 mg, 76%) as a white solid. MS (ESI) *m/z* 618.2 [M + H]^+^, HRMS (ESI) *m/z*: [M + H]^+^ calculated for C_33_H_33_FN_3_O_6_S, 618.2069, found 618.2069. ^1^H NMR (600 MHz, DMSO-*d*_6_) *δ* 8.81 (d, *J* = 8.0 Hz, 1H), 8.44 (d, *J* = 8.6 Hz, 1H), 7.85 – 7.81 (m, 2H), 7.78 (d, *J* = 7.7 Hz, 1H), 7.73 – 7.70 (m, 1H), 7.68 – 7.61 (m, 3H), 7.57 (s, 1H), 7.46 (s, 1H), 7.41 (s, 1H), 7.35 – 7.29 (m, 3H), 7.04 (t, *J* = 8.8 Hz, 2H), 6.88 (dd, *J* = 15.1, 4.6 Hz, 1H), 6.48 (d, *J* = 15.1 Hz, 1H), 4.68 – 4.60 (m, 2H), 3.12 – 2.98 (m, 4H), 2.25 – 2.17 (m, 1H), 2.11 – 2.03 (m, 1H), 1.83 – 1.78 (m, 1H), 1.68 – 1.63 (m, 1H), 1.60 – 1.52 (m, 1H), 1.51 – 1.43 (m, 1H), 1.33 – 1.25 (m, 1H). ^13^C NMR (150 MHz, DMSO-*d*_6_) *δ* 172.5, 170.6, 160.9 (d, *J* = 242.2 Hz), 158.0, 154.1, 148.5, 147.6, 140.2, 133.8, 133.6, 130.9 (d, *J* = 8.0 Hz), 129.5, 129.2, 127.0, 127.0, 126.9, 123.6, 122.8, 114.8 (d, *J* = 21.0 Hz), 111.8, 109.7, 54.7, 46.4, 41.1, 37.0, 35.9, 34.5, 25.3, 21.1. ^19^F NMR (471 MHz, DMSO-*d*_6_) *δ* -116.3 – -116.4 (m).

*N-((2S)-3-(cyclohexa-2,4-dien-1-yl)-1-oxo-1-(((S,E)-1-((S)-2-oxopiperidin-3-yl)-4-(phenylsulfonyl)but-3-en-2-yl)amino)propan-2-yl)benzofuran-2-carboxamide (****14d****)*

The synthesis procedure is similar to that of **14c**. MS (ESI) *m/z* 606.4 [M + H]^+^, HRMS (ESI) *m/z*: [M + H]^+^ calculated for C_33_H_40_N_3_O_6_S, 606.2632, found 606.2631. ^1^H NMR (500 MHz, DMSO-*d*_6_) *δ* 8.61 (d, *J* = 7.5 Hz, 1H), 8.40 (d, *J* = 8.7 Hz, 1H), 7.83 – 7.77 (m, 3H), 7.72 – 7.65 (m, 2H), 7.63 – 7.59 (m, 3H), 7.49 – 7.45 (m, 1H), 7.41 – 7.38 (m, 1H), 7.36 – 7.32 (m, 1H), 6.93 (dd, *J* = 15.1, 4.6 Hz, 1H), 6.61 (dd, *J* = 15.1, 1.6 Hz, 1H), 4.71 – 4.62 (m, 1H), 4.46 – 4.39 (m, 1H), 3.12 – 2.99 (m, 2H), 2.24 – 2.17 (m, 1H), 2.12 – 2.04 (m, 1H), 1.85 – 1.41 (m, 13H), 1.34 – 1.21 (m, 2H), 1.18 – 0.99 (m, 1H), 0.95 – 0.82 (m, 2H). ^13^C NMR (125 MHz, DMSO-*d*_6_) *δ* 172.5, 171.6, 158.1, 154.2, 148.6, 147.7, 140.2, 133.6, 129.5, 129.3, 127.0 (127.04, 126.98), 126.8, 123.6, 122.7, 111.8, 109.7, 51.3, 46.2, 41.1, 38.6, 37.1, 34.4, 33.6, 32.6, 32.1, 25.9, 25.6, 25.5, 25.3, 21.1.

**Synthesis of compounds 14e to 14h**

**Scheme S4** Reagents and conditions: **a**, AcOH, EtOH, reflux, 4 h.

*N-((S)-1-(((S,E)-1-(2-benzoylhydrazineylidene)-3-((S)-2-oxopiperidin-3-yl)propan-2-yl)amino)-3-(4-fluorophenyl)-1-oxopropan-2-yl)benzofuran-2-carboxamide (****14e****)*

To a solution of intermediate **11a** (100 mg, 0.21 mmol) and benzohydrazide (31.2 mg, 0.23 mmol) in dry ethanol (10 mL) was added catalytic amount of acetic acid, and the mixture was refluxed at 80 ℃ for 4 h. Then the mixture was evaporated to afford the crude product, which was further purified by column chromatograph (DCM/MeOH = 20:1, v/v) to get product **14e** (91.9 mg, 75%) as a white solid. MS (ESI) *m/z* 598.3 [M + H]^+^, HRMS (ESI) *m/z*: [M + H]^+^ calculated for C_33_H_33_FN_5_O_5_, 598.2460, found 598.2466. ^1^H NMR (500 MHz, DMSO-*d*_6_) *δ* 11.56 (s, 1H), 8.78 (d, *J* = 8.5 Hz, 1H), 8.53 (d, *J* = 8.8 Hz, 1H), 7.88 – 7.83 (m, 2H), 7.80 – 7.75 (m, 1H), 7.71 – 7.63 (m, 2H), 7.60 – 7.54 (m, 2H), 7.53 – 7.44 (m, 3H), 7.43 – 7.31 (m, 4H), 7.10 – 7.03 (m, 2H), 4.77 – 4.57 (m, 2H), 3.20 – 3.01 (m, 4H), 2.35 – 2.19 (m, 2H), 1.98 – 1.86 (m, 1H), 1.77 – 1.67 (m, 1H), 1.66 – 1.48 (m, 2H), 1.46 – 1.32 (m, 1H). ^13^C NMR (125 MHz, DMSO-*d*_6_) *δ* 172.6, 170.6, 162.9, 160.9 (d, *J* = 241.9 Hz), 158.0, 154.1, 151.1, 148.5, 134.1 (d, *J* = 2.5 Hz), 133.3, 131.6, 130.9 (d, *J* = 8.0 Hz), 128.3, 127.5, 127.0, 126.8, 123.6, 122.7, 114.7 (d, *J* = 21.0 Hz), 111.8, 109.7, 54.6, 47.8, 41.1, 37.0, 36.2, 33.3, 25.6, 21.3. ^19^F NMR (471 MHz, DMSO-*d*_6_) *δ* -116.7 – -116.8 (m).

*N-((S)-1-(((S,E)-1-(2-(cyclohexanecarbonyl)hydrazineylidene)-3-((S)-2-oxopiperidin-3-yl)propan-2-yl)amino)-3-(4-fluorophenyl)-1-oxopropan-2-yl)benzofuran-2-carboxamide (****14f****)*

The synthesis procedure is similar to that of **14e**. MS (ESI) *m/z* 604.5 [M + H]^+^, HRMS (ESI) *m/z*: [M + H]^+^ calculated for C_33_H_39_FN_5_O_5_, 604.2930, found 604.2931. *E*/*Z* isomers apparent in ^1^H NMR, ^13^C NMR and ^19^F NMR spectrum. ^1^H NMR (500 MHz, DMSO-*d*_6_) *δ* 10.96 and 10.76 (2 × s, 1H), 8.74 (t, *J* = 8.4 Hz, 1H), 8.45 (d, *J* = 8.6 Hz, 1H), 7.77 (d, *J* = 7.8 Hz, 1H), 7.66 (d, *J* = 8.3 Hz, 1H), 7.59 – 7.54 (m, 1H), 7.49 – 7.43 (m, 1H), 7.42 – 7.24 (m, 5H), 7.10 – 7.02 (m, 2H), 4.78 – 4.47 (2 × m, 2H), 3.18 – 2.99 (m, 4H), 2.28 – 2.04 (2 × m, 2H), 1.94 – 1.85 (m, 1H), 1.76 – 1.46 (m, 8H), 1.43 – 1.07 (m, 7H). ^13^C NMR (125 MHz, DMSO-*d*_6_) *δ* 176.6, 172.6 (172.63, 172.60), 171.4, 170.5 (170.53, 170.47), 161.8, 159.9, 157.9, 154.1, 149.0, 148.5, 145.7, 134.1, 131.0, 130. 9, 127.0, 126.8, 123.6, 122.7, 114.8, 114.6, 111.8, 109. 7, 54.6, 54.4, 47.6, 47.2, 42.7, 41.1, 37.1, 36.9, 36.5, 36.2, 33.5, 33.3, 28.9, 28.1, 25.8, 25.6, 25.4, 25.3, 25.1, 21.2. ^19^F NMR (471 MHz, DMSO-*d*_6_) *δ* -116.7 – -116.8 (m).

*N-((S)-1-(((S,E)-1-(2-(cyclopropanecarbonyl)hydrazineylidene)-3-((S)-2-oxopiperidin-3-yl)propan-2-yl)amino)-3-(4-fluorophenyl)-1-oxopropan-2-yl)benzofuran-2-carboxamide (****14g****)*

The synthesis procedure is similar to that of **14e**. MS (ESI) *m/z* 562.6 [M + H]^+^, HRMS (ESI) *m/z*: [M + H]^+^ calculated for C_30_H_33_FN_5_O_5_, 562.2460, found 562.2462. *E*/*Z* isomers apparent in ^1^H NMR, ^13^C NMR and ^19^F NMR spectrum. ^1^H NMR (500 MHz, DMSO-*d*_6_) *δ* 11.28 and 11.03 (2 × s, 1H), 8.79 – 8.73 (m, 1H), 8.47 and 8.42 (2 × d, *J* = 8.4 Hz, 1H), 7.77 (d, *J* = 7.8 Hz, 1H), 7.66 (d, *J* = 8.4 Hz, 1H), 7.57 and 7.56 (2 × s, 1H), 7.46 (t, *J* = 7.8 Hz, 1H), 7.42 – 7.26 (m, 5H), 7.10 – 7.03 (m, 2H), 4.72 – 4.64 (m, 1H), 4.63 – 4.49 (m, 1H), 3.15 – 2.99 (m, 4H), 2.48 – 2.40 and 2.09 – 1.97 (2 × m, 1H), 2.29 – 2.13 (m, 2H), 1.95 – 1.80 (2 × m, 1H), 1.76 – 1.43 (m, 3H), 1.42 – 1.30 (m, 1H), 0.80 – 0.63 (2 × m, 4H). ^13^C NMR (125 MHz, DMSO-*d*_6_) *δ* 174.2, 172.7, 172.6 (172.62, 172.60), 170.5, 169.0, 161.8, 159.9, 157.9 (157.92, 157.87), 154.1, 148.5, 146.0, 134.1, 134.0, 130.9 (130.94, 130.87), 130.8, 127.0, 126.9, 126.8, 123.6, 122.7, 114.8, 114.7, 111.8, 109.7, 54.6, 47.6, 47.3, 41.1, 37.0, 36.9, 36.3, 36.2, 33.4, 33.4, 25.6, 21.2, 12.6, 9.5, 7.5 (7.51, 7.47), 6.6. ^19^F NMR (471 MHz, DMSO-*d*_6_) *δ* -116.6 – -116.8 (m).

*N-((S)-3-(4-fluorophenyl)-1-oxo-1-(((S,E)-1-((S)-2-oxopiperidin-3-yl)-3-(2-(pyrimidin-2-yl)hydrazineylidene)propan-2-yl)amino)propan-2-yl)benzofuran-2-carboxamide (****14h****)*

The synthesis procedure is similar to that of **14e**. MS (ESI) *m/z* 572.3 [M + H]^+^, HRMS (ESI) *m/z*: [M + H]^+^ calculated for C_30_H_31_FN_7_O_4_, 572.2416, found 572.2416. ^1^H NMR (600 MHz, DMSO-*d*_6_) *δ* 10.87 (s, 1H), 8.76 (d, *J* = 8.3 Hz, 1H), 8.47 (d, *J* = 8.8 Hz, 1H), 8.37 (d, *J* = 4.8 Hz, 2H), 7.77 (d, *J* = 7.8 Hz, 1H), 7.66 (d, *J* = 8.4 Hz, 1H), 7.57 (s, 1H), 7.49 – 7.43 (m, 1H), 7.41 (s, 1H), 7.38 – 7.30 (m, 4H), 7.06 (t, *J* = 8.8 Hz, 2H), 6.76 (t, *J* = 4.8 Hz, 1H), 4.71 – 4.65 (m, 1H), 4.64 – 4.56 (m, 1H), 3.18 – 3.01 (m, 4H), 2.34 – 2.19 (m, 2H), 1.95 – 1.90 (m, 1H), 1.74 – 1.66 (m, 1H), 1.63 – 1.47 (m, 2H), 1.40 – 1.32 (m, 1H). ^13^C NMR (150 MHz, DMSO-*d*_6_) *δ* 172.7, 170.5, 160.9 (d, *J* = 241.9 Hz), 156.0, 158.1, 157.9, 154.1, 148.6, 144.6, 134.2, 130.9 (d, *J* = 8.1 Hz), 127.0, 126.8, 123.6, 122.7, 114.7 (d, *J* = 21.0 Hz), 112.6, 111.8, 109.7, 54.6, 47.6, 41.1, 37.0, 36.2, 33.6, 25.6, 21.3. ^19^F NMR (471 MHz, DMSO-*d*_6_) *δ* -116.7 – -116.8 (m).

**Synthesis of compounds 14i to 14k**

**Scheme S5.** Reagents and conditions: **a**, TEA, MgBr_2_, THF, r.t., 1 h; **b**, LiOH·H_2_O, MeOH/H_2_O, r.t., 2 h; **c**, HATU, DIPEA, DCM, -20 °C, 12 h.

*Ethyl (S,Z)-4-((S)-2-(benzofuran-2-carboxamido)-3-cyclohexylpropanamido)-2-fluoro-5-((S)-2-oxopiperidin-3-yl)pent-2-enoate (****14j****)*

To a solution of **11b** (3.00 g, 6.42 mmol) in anhydrous THF (50 mL) was added ethyl 2-(diethoxyphosphoryl)-2-fluoroacetate (2.33 g, 9.62 mmol), triethylamine (1.95 g, 19.25 mmol) and magnesium bromide (1.18 g, 6.42 mmol), and the resulting mixture was stirred at room temperature for 24 h. The mixture was added EA (100 mL), washed with saturated NH_4_Cl solution (50 mL×3), saturated NaHCO_3_ solution (50 mL×3), and brine (50 mL×3). The organic layer was dried over Na_2_SO_4_ and concentrated under reduced pressure. The residue was further purified by flash column chromatography (DCM/MeOH = 100:1 to 50:1, v/v) to obtain white solid **14j** (2.25 g, 63%). MS (ESI) *m/z* 556.4 [M + H]^+^, HRMS (ESI) *m/z*: [M + H]^+^ calculated for C_30_H_39_FN_3_O_6_, 556.2817, found 556.2812. ^1^H NMR (500 MHz, DMSO-*d*_6_) *δ* 8.62 (d, *J* = 8.0 Hz, 1H), 8.26 (d, *J* = 8.6 Hz, 1H), 7.78 (d, *J* = 7.7 Hz, 1H), 7.67 (d, *J* = 8.4 Hz, 1H), 7.63 (s, 1H), 7.50 – 7.43 (m, 1H), 7.39 (s, 1H), 7.33 (t, *J* = 7.5 Hz, 1H), 5.97 (dd, *J* = 20.9, 9.4 Hz, 1H), 5.35 – 5.25 (m, 1H), 4.48 – 4.40 (m, 1H), 4.26 (q, *J* = 7.1 Hz, 2H), 3.12 – 3.02 (m, 2H), 2.32 – 2.16 (m, 2H), 1.98 – 1.90 (m, 1H), 1.75 – 1.44 (m, 9H), 1.42 – 1.22 (m, 3H), 1.27 (t, *J* = 7.1 Hz, 3H), 1.20 – 1.04 (m, 3H), 0.96 – 0.82 (m, 2H). ^13^C NMR (125 MHz, DMSO-*d*_6_) *δ* 172.6, 171.4, 159.6 (d, *J* = 36.2 Hz), 158.0, 154.1, 148.7, 145.7 (d, *J* = 253.3 Hz), 127.1, 126.8, 124.2 (d, *J* = 16.6 Hz), 123.6, 122.7, 111.8, 109.7, 61.5, 51.0, 42.3 (d, *J* = 7.8 Hz), 41.1, 38.8, 37.0, 35.6, 33.6, 33.0, 31.8, 26.0, 25.7, 25.6, 25.3, 21.1, 13.8. ^19^F NMR (471 MHz, DMSO-*d*_6_) *δ* -123.0 (d, *J* = 21.0 Hz).

*(S,Z)-4-((S)-2-(benzofuran-2-carboxamido)-3-cyclohexylpropanamido)-2-fluoro-5-((S)-2-oxopiperidin-3-yl)pent-2-enoic acid (****14i****)*

Compounds **14j** (1.00 g, 1.80 mmol) was dissolved in MeOH (20 mL) and water (10 mL), and then LiOH·H_2_O (0.15 g, 3.60 mmol) was added. The mixture was stirred at room temperature for 2 h. Subsequently, 1 M HCl aqueous solution was added to adjust the pH to 1~3, and the resulting mixture was extracted with EA (30 mL×3). The combined organic phase was washed with brine (30 mL×2), dried over Na_2_SO_4_, and concentrated *in vacuum*. The residual solid was purified by column chromatography (DCM/MeOH = 30:1 to 15:1, v/v) to yield **14i** (0.34 g, 36%), as a white solid. MS (ESI) *m/z* 528.4 [M + H]^+^, HRMS (ESI) *m/z*: [M + H]^+^ calculated for C_28_H_35_FN_3_O_6_, 528.2504, found 528.2504. ^1^H NMR (500 MHz, DMSO-*d*_6_) *δ* 13.88 (s, 1H), 8.61 (d, *J* = 7.9 Hz, 1H), 8.24 (d, *J* = 8.7 Hz, 1H), 7.78 (d, *J* = 7.8 Hz, 1H), 7.68 (d, *J* = 8.4 Hz, 1H), 7.63 (s, 1H), 7.47 (t, *J* = 7.8 Hz, 1H), 7.37 (s, 1H), 7.34 (t, *J* = 7.5 Hz, 1H), 5.88 (dd, *J* = 20.8, 9.2 Hz, 1H), 5.35 – 5.25 (m, 1H), 4.48 – 4.41 (m, 1H), 3.12 – 3.02 (m, 2H), 2.30 – 2.14 (m, 2H), 1.95 – 1.88 (m, 1H), 1.75 – 1.43 (m, 9H), 1.40 – 1.22 (m, 3H), 1.20 – 1.05 (m, 3H), 0.96 – 0.82 (m, 2H). ^13^C NMR (125 MHz, DMSO-*d*_6_) *δ* 172.6, 171.4, 161.2 (d, *J* = 36.1 Hz), 158.0, 154.2, 148.7, 146.6 (d, *J* = 253.5 Hz), 127.1, 126.8, 123.6, 123.5 (d, *J* = 17.5 Hz), 122.7, 111.8, 109.7, 51.0, 42.5 (d, *J* = 7.9 Hz), 41.1, 38.8, 37.0, 35.9, 33.6, 33.0, 31.8, 26.0, 25.7, 25.6, 25.2, 21.0. ^19^F NMR (471 MHz, DMSO-*d*_6_) *δ* -121.0 (brs).

*N-((S)-3-cyclohexyl-1-(((S,Z)-5-(cyclopropylamino)-4-fluoro-5-oxo-1-((S)-2-oxopiperidin-3-yl)pent-3-en-2-yl)amino)-1-oxopropan-2-yl)benzofuran-2-carboxamide (****14k****)*

To a solution of **14i** (100 mg, 0.19 mmol) in DCM (10 mL), which was cooled to -20 °C, HATU (108 mg, 0.28 mmol) was added, and the resulting mixture was stirred at -20 °C for 30 min. Then cyclopropylamine (15.9 mg, 0.28 mmol) and DIPEA (71.9 mg, 0.56 mmol) were added, and the mixture was stirred at -20 °C for 12 h. The mixture was washed with saturated NH_4_Cl solution (30 mL×3), saturated NaHCO_3_ solution (30 mL×3), and brine (30 mL×3). The organic layer was dried over Na_2_SO_4_ and concentrated under reduced pressure. The residue was further purified by flash column chromatography (DCM/MeOH = 30:1 to 20:1, v/v) to obtain white solid **14k** (56.2 mg, 52%). MS (ESI) *m/z* 567.5 [M + H]^+^, HRMS (ESI) *m/z*: [M + H]^+^ calculated for C_31_H_40_FN_4_O_5_, 567.2977, found 567.2977. ^1^H NMR (500 MHz, DMSO-*d*_6_) *δ* 8.63 (d, *J* = 8.0 Hz, 1H), 8.57 (d, *J* = 4.6 Hz, 1H), 8.26 (d, *J* = 8.7 Hz, 1H), 7.78 (d, *J* = 7.8 Hz, 1H), 7.67 (d, *J* = 8.3 Hz, 1H), 7.63 (s, 1H), 7.47 (t, *J* = 7.8 Hz, 1H), 7.37 (s, 1H), 7.33 (t, *J* = 7.5 Hz, 1H), 5.62 (dd, *J* = 23.0, 9.2 Hz, 1H), 5.37 – 5.28 (m, 1H), 4.49 – 4.41 (m, 1H), 3.10 – 3.05 (m, 2H), 2.76 – 2.69 (m, 1H), 2.25 – 2.14 (m, 2H), 1.96 – 1.90 (m, 1H), 1.73 – 1.52 (m, 10H), 1.33 – 1.28 (m, 1H), 1.16 – 1.09 (m, 4H), 0.96 – 0.82 (m, 2H), 0.66 – 0.51 (m, 4H). ^13^C NMR (125 MHz, DMSO-*d*_6_) *δ* 172.8, 171.5, 160.5 (d, *J* = 32.3 Hz), 158.0, 154.2, 148.7 (148.68, 148.67 (d, *J* = 262.7 Hz)), 127.1, 126.8, 123.6, 122.7, 119.1 (d, *J* = 16.4 Hz), 111.8, 109.7, 51.0, 42.3 (d, *J* = 8.7 Hz), 41.2, 38.8, 37.1, 36.0, 33.6, 33.0, 31.8, 26.0, 25.7, 25.6, 25.4, 22.3, 21.1, 5.5, 5.4. ^19^F NMR (471 MHz, DMSO-*d*_6_) *δ* -121.1 (d, *J* = 23.1 Hz).

**Pharmacokinetics of compounds 14a and 14b**

*Administration and blood sample collection of compounds* ***14a*** *and* ***14b*** *in mice*

The intravenous (i.v.), intraperitoneal (i.p.) and oral (p.o.) pharmacokinetics of **14a** and **14b** were evaluated in mice. A group of 9 male ICR mice weighing 18 to 25 g was administered an intravenous bolus dose of 5 mg/kg **14a** in a vehicle consisting of 5% dimethyl sulfoxide, 7.5% Kolliphor HS 15 and 87.5% saline (volume ratio). Serial blood samples were collected at 0.083 (animals 1 to 3), 0.25 (animals 4 to 6), 0.75 (animals 7 to 9), 2 (animals 1 to 3), 4 (animals 4 to 6), 6 (animals 7 to 9), 8 (animals 1 to 3) and 24 h (animals 4 to 6) after dose administration. The procedure for intraperitoneal (5 mg/kg) and oral (10 mg/kg) studies of **14a** in mice was similar to that of the intravenous group, except for time point for collecting blood sample for oral studies at 0.25, 0.75, 1.00, 2.00, 4.00 and 6.00 h after dosing. Two groups of male CD-1 mice (3 animals per group) weighing 18 to 22 g were intravenously (10 mg/kg) and intraperitoneally (20 mg/kg) dosed **14b** in a vehicle consisting of 5% dimethyl sulfoxide, 5% ethanol, 40% polyethylene glycol 400 and 50% saline (volume ratio). Serial blood samples were collected at 0.05, 0.25, 0.75, 2.00, 4.00, 8.00 and 24 h after dose administration. For oral study of **14b** (20 mg/kg) in CD-1 mice, **14b** was formulated in 5% dimethyl sulfoxide and 95% hydroxypropyl methyl cellulose solution (0.5% hydroxypropyl methyl cellulose in deionized water in weight ratio) (volume ratio) and then dosed as an oral gavage. Blood samples were collected at 0.25, 0.50, 1.00, 2.00, 4.00, 8.00 and 24.0 h after dose administration. All blood samples were obtained by centrifugation and stored at -20 °C until analysis.

*Administration and blood sample collection of compounds* ***14a*** *and* ***14b*** *in rats*

Three groups of male SD rats weighing 180 to 240 g (3 animals per group, 2 animals for intraperitoneal group) were intravenously (5 mg/kg), intraperitoneally (10 mg/kg) and orally (10 mg/kg) dosed **14a** in a vehicle consisting of 5% dimethyl sulfoxide, 7.5% Kolliphor HS 15 and 87.5% saline (volume ratio). Serial blood samples were collected at 0.083, 0.25, 0.75, 2.0, 4.0, 6.0, 8.0 and 24.0 h for intravenous and intraperitoneal groups and 0.25, 0.5, 1.0, 2.0, 4.0, 6.0, 8.0 and 24.0 h for the oral group after dose administration. Two groups of male SD rats weighing 180 to 240 g (3 animals per group) were intravenously (5 mg/kg) and intraperitoneally (10 mg/kg) dosed **14b** in a vehicle consisting of 5% dimethyl sulfoxide, 5% ethanol, 40% polyethylene glycol 400 and 50% saline (volume ratio). Serial blood samples were collected at 0.083, 0.25, 0.50, 1.00, 2.00, 4.00, 8.00 and 24.0 h after dose administration. A group of 3 male SD rats weighing 180 to 240 g was administered an oral dose of 10 mg/kg **14b** in a vehicle consisting of 5% dimethyl sulfoxide and 95% hydroxypropyl methyl cellulose solution (0.5% hydroxypropyl methyl cellulose in deionized water in weight ratio) (volume ratio). Serial blood samples were collected at 0.25, 0.50, 1.00, 2.00, 4.00, 8.00 and 24.0 h after dose administration. All blood samples were obtained by centrifugation and stored at -20 °C until analysis.

*Administration and blood sample collection of compounds* ***14a*** *and* ***14b*** *in dogs*

Three groups of male Beagle dogs weighing 9 to 12 kg (2 animals per group) were intravenously (1 mg/kg), subcutaneously (1 mg/kg) and orally (5 mg/kg) dosed **14a** in a vehicle consisting of 5% dimethyl sulfoxide, 7.5% Kolliphor HS 15 and 87.5% saline (volume ratio). Serial blood samples were collected at 0.083, 0.25, 0.75, 2.0, 4.0, 6.0, 8.0 and 24.0 h for the intravenous and intraperitoneal groups and 0.25, 0.5, 1.0, 2.0, 4.0, 6.0, 8.0 and 24.0 h for the oral group after dose administration. Two groups of male Beagle dogs weighing 9 to 12 kg (3 animals per group) were intravenously (1 mg/kg) and subcutaneously (1 mg/kg) dosed **14b** in a vehicle consisting of 5% dimethyl sulfoxide, 5% ethanol, 40% polyethylene glycol 400 and 50% saline (volume ratio). Serial blood samples were collected at 0.083, 0.25, 0.50, 1.00, 2.00, 4.00, 8.00 and 24.0 h after dose administration. A group of 3 male Beagle dogs weighing 9 to 12 kg was administered an oral dose of 5 mg/kg **14b** in a vehicle consisting of 5% dimethyl sulfoxide and 95% hydroxypropyl methyl cellulose solution (0.5% hydroxypropyl methyl cellulose in deionized water in weight ratio) (volume ratio). Serial blood samples were collected at 0.25, 0.50, 1.00, 2.00, 4.00, 8.00 and 24.0 h after dose administration. All blood samples were obtained by centrifugation and stored at -20 °C until analysis.

*Analysis of pharmacokinetics parameters*

Plasma compound concentrations were obtained through HPLC-MS/MS, and the pharmacokinetics parameters were analyzed by Phoenix WinNonlin 7.0. The following parameters were determined: C_max_ (maximal concentration of drug in plasma), AUC_last_ or AUC_INF_obs_ (area under the concentration curve from the time of dosing to the last measurable concentration or from the time of dosing to infinity, respectively). T_max_ (time of maximum observed concentration), T_1/2_ (half-life of elimination), MRT_INF_obs_ (mean residence time extrapolated to infinity for a substance administered by indicated dosing using observed last concentration), CL (total body clearance), V_ss_ (volume of distribution at steady state) and F (absolute bioavailability, F = (AUC_x_ × D_i.v._)/( AUCi.v. × D_x_) × 100%, where x is i.p., s.c. or p.o., and AUC is AUC_INF_obs_).The pharmacokinetic parameters were analyzed by Phoenix WinNonlin 7.0.


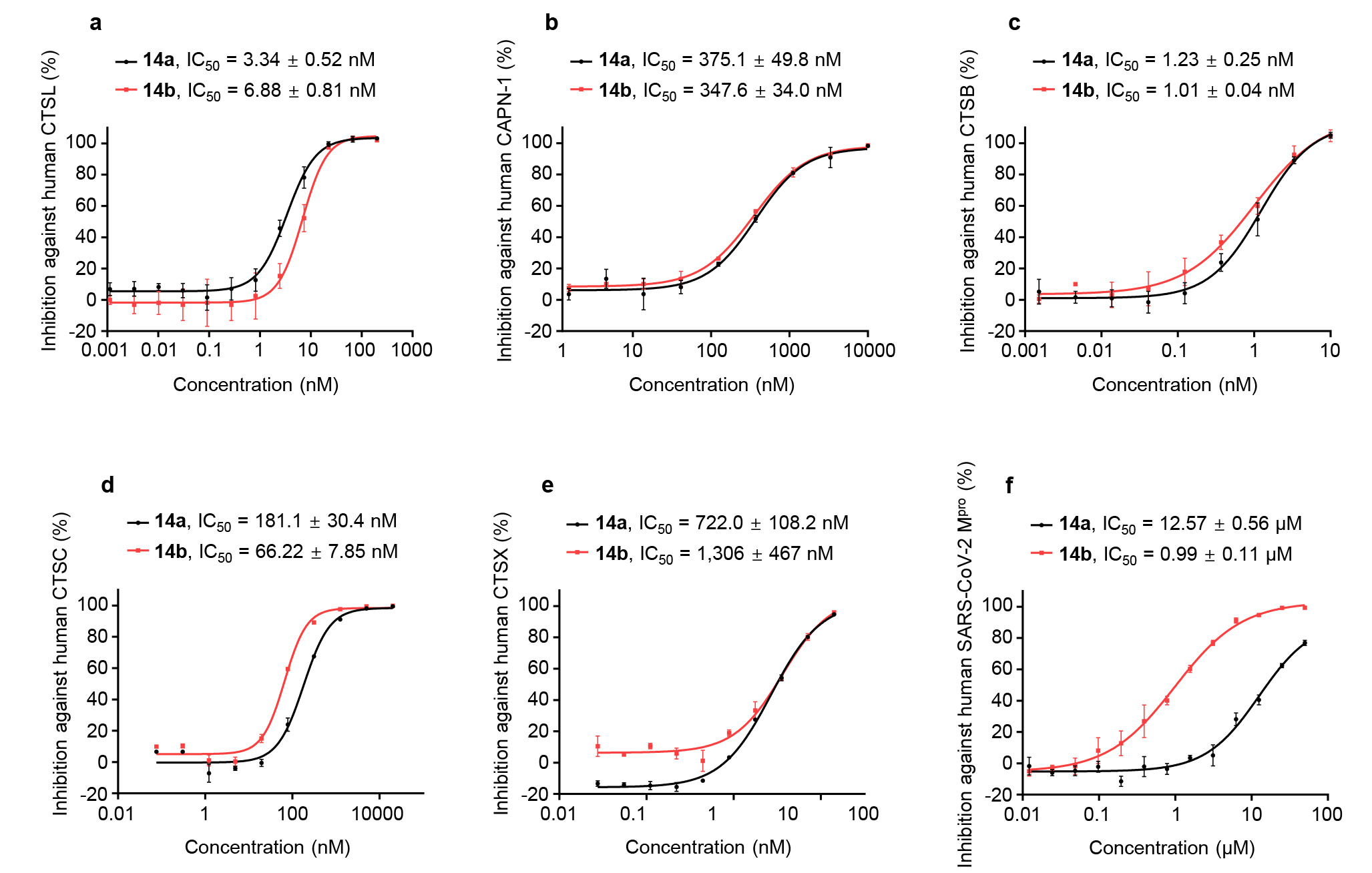


**Fig. S1** **Enzymatic inhibitory activity against human CTSL, CAPN1, CTSB, CTSC, CTSX as well as SARS-CoV-2 M^pro^ of 14a and 14b. a** Inhibition of **14a** and **14b** against human CTSL. **b** Inhibition of **14a** and **14b** against human CAPN1. **c** Inhibition of **14a** and **14b** against human CTSB. **d** Inhibition of **14a** and **14b** against human CTSC. **e** Inhibition of **14a** and **14b** against human CTSX. **f** Inhibition of **14a** and **14b** against SARS-CoV-2 M^pro^. Each sample was tested in triplicate, and the experiment was repeated twice. Data from a representative experiment are shown as mean ± SD.


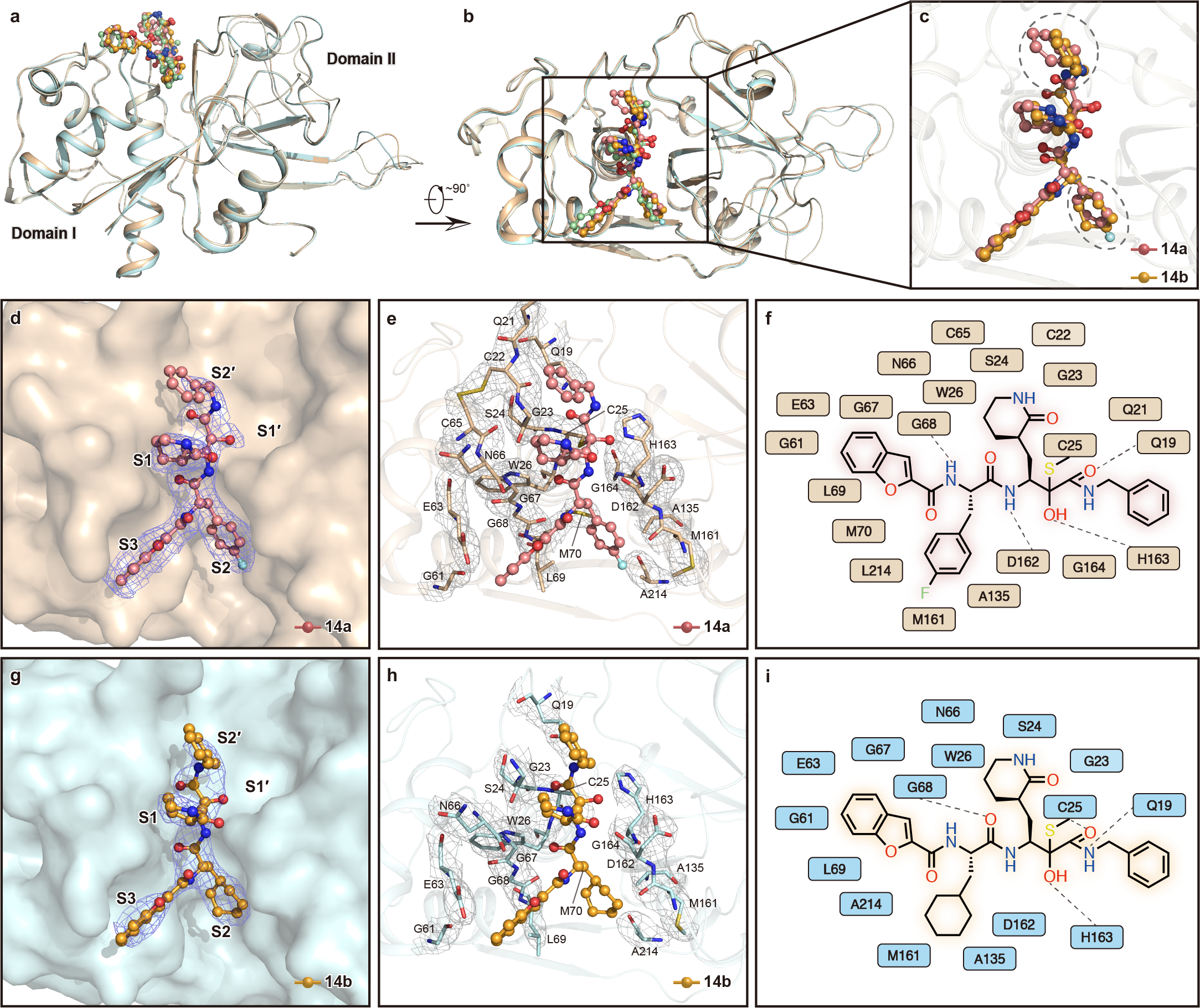


**Fig. S2 Crystal structures of human CTSL in complex with 14a and 14b, respectively.** **a, b** Cartoon representation of the crystal structure of human CTSL in complex with **14a** and **14b**. Both compounds are shown as ball-and-stick models with the carbon atoms in salmon **(14a)**, bright orange **(14b)**, oxygen atoms in bright red, nitrogen atoms in blue, and fluorine atom in cyan (the same as below, unless otherwise indicated). **c** A comparison of the binding modes of **14a** and **14b** in human CTSL substrate-binding pocket. The major difference between both compounds is marked with a dashed rounded circle. **d** Close-up view of **14a** binding pocket. Five subsites, S2′, S1′, S1, S2, and S3, are labeled. The polder electron density map of **14a** is colored in blue mesh and contoured at 2.5σ. **e** The 2*F_o_-F_c_* electron density map for residues involved in **14a** binding is shown as light gray mesh and contoured at 1σ. **f** Schematic diagram of human CTSL-**14a** interactions shown in **(e)**. **g** Close-up view of **14b** binding pocket. Five subsites, S2′, S1′, S1, S2, and S4, are labeled. The polder electron density map of **14b** is colored in blue mesh and contoured at 2.5σ. **h** The 2*F_o_-F_c_* electron density map for residues involved in **14b** binding is shown as light gray mesh and contoured at 1σ. **i** Schematic diagram of human CTSL-**14b** interactions shown in **(h)**.


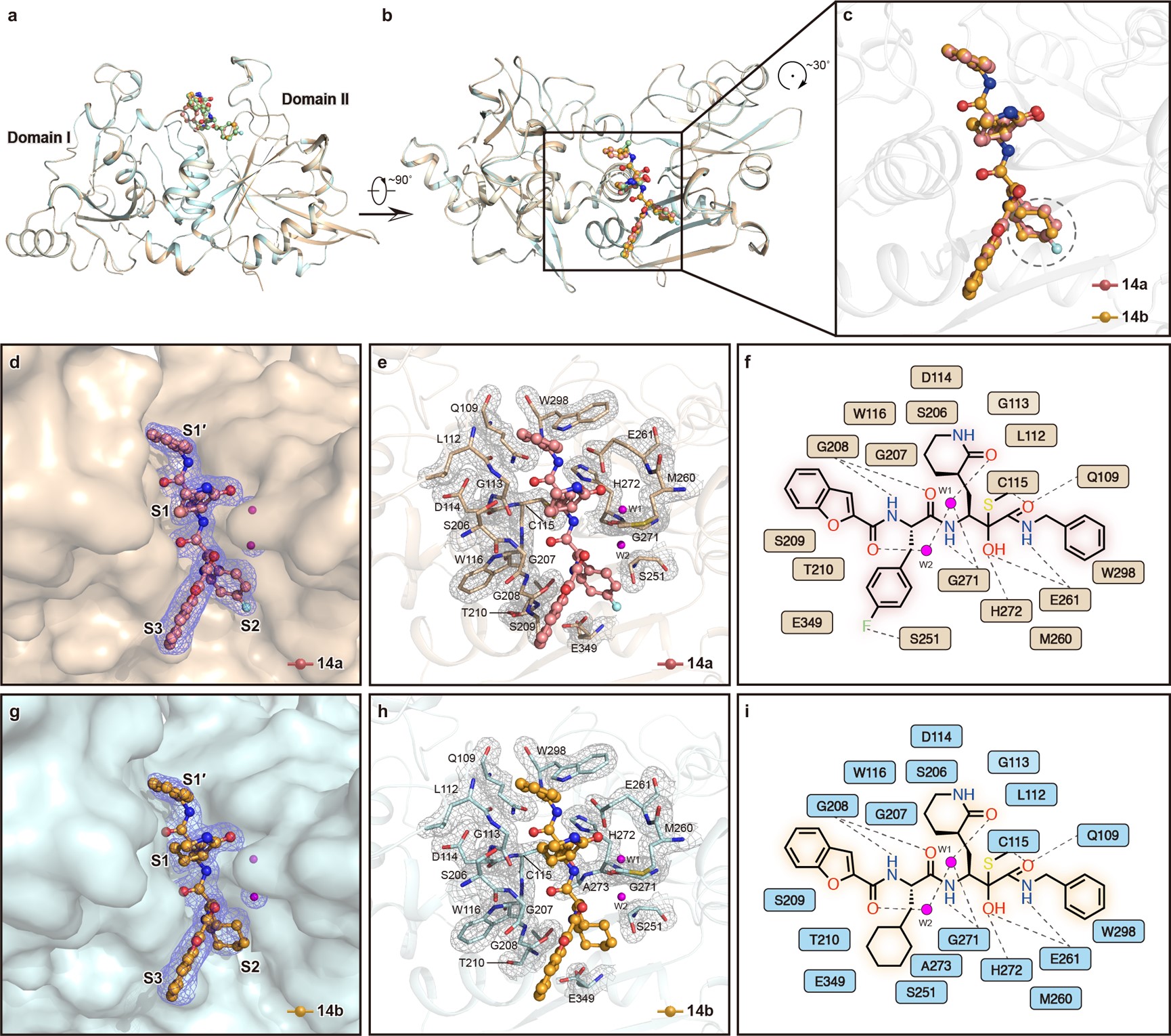


**Fig. S3 Crystal structures of human CAPN1 protease core in complex with 14a and 14b, respectively. a, b** Cartoon representation of the crystal structure of human CAPN1 protease core in complex with **14a** and **14b**. Both compounds are shown as ball-and-stick models with the carbon atoms in salmon **(14a)**, bright orange **(14b)**, oxygen atoms in bright red, nitrogen atoms in blue, and fluorine atom in cyan (the same as below, unless otherwise indicated). **c** A comparison of the binding modes of **14a** and **14b** in human CAPN1 protease core substrate-binding pocket. The major difference between both compounds is marked with a dashed rounded circle. **d** Close-up view of **14a** binding pocket. Four subsites, S1′, S1, S2, and S3, are labeled. The polder electron density map of **14a** is colored in blue mesh and contoured at 3σ. **e** The 2*F_o_-F_c_* electron density map for residues involved in **14a** binding is shown as light gray mesh and contoured at 1σ. **f** Schematic diagram of human CAPN1 protease core-**14a** interactions shown in **(e)**. **g** Close-up view of **14b** binding pocket. Four subsites, S1′, S1, S2, and S3, are labeled. The polder electron density map of **14b** is colored in blue mesh and contoured at 3σ. **h** The 2*F_o_-F_c_* electron density map for residues involved in **14b** binding is shown as light gray mesh and contoured at 1σ. **i** Schematic diagram of human CAPN1 protease core-**14b** interactions shown in **(h)**.


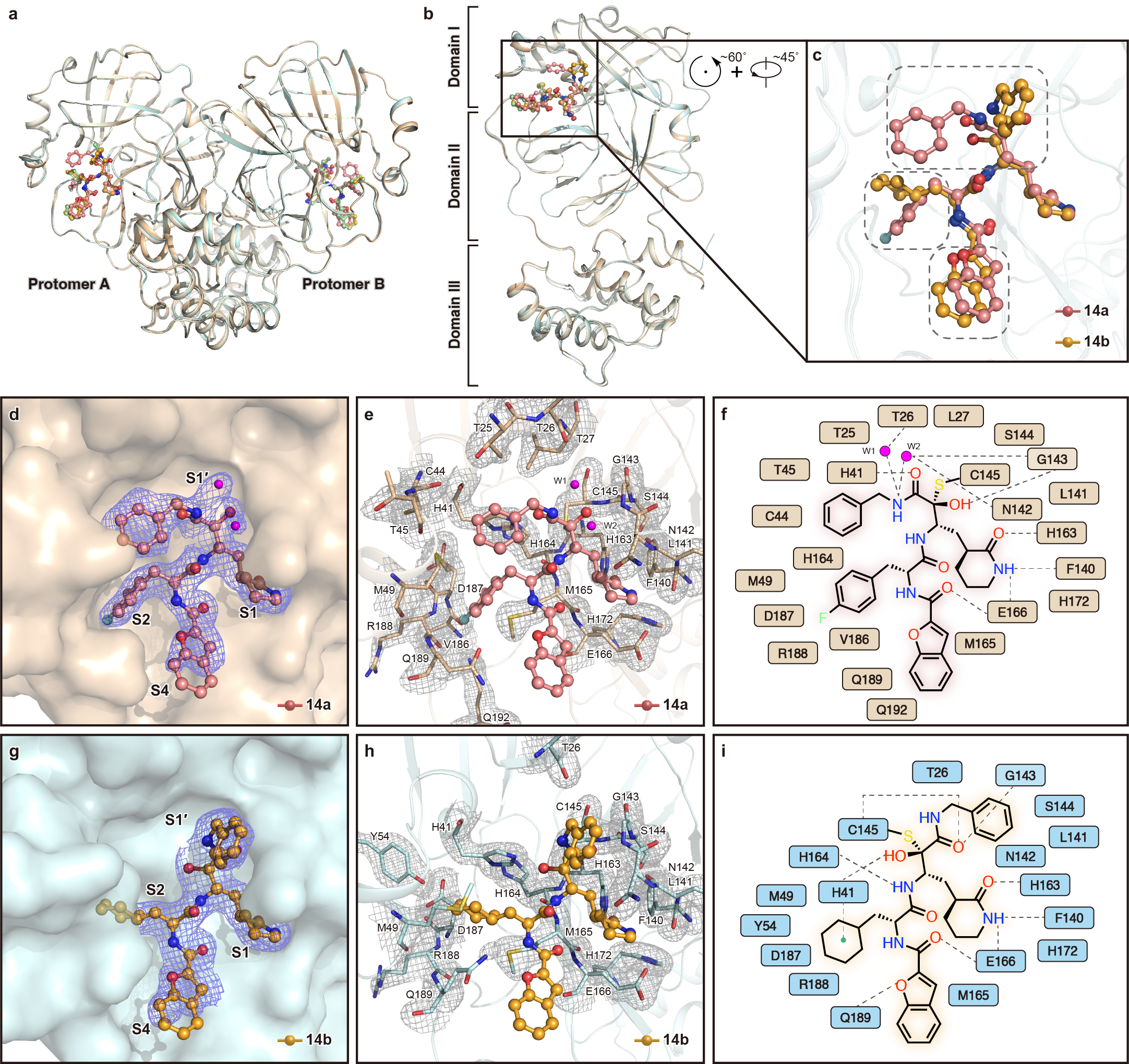


**Fig. S4 Crystal structures of SARS-CoV-2 M^pro^ in complex with 14a and 14b, respectively.** **a, b** Cartoon representation of the crystal structure of SARS-CoV-2 M^pro^ in complex with **14a** and **14b**. Both compounds are shown as ball-and-stick models with the carbon atoms in salmon **(14a)**, bright orange **(14b)**, oxygen atoms in bright red, nitrogen atoms in blue, and fluorine atom in cyan (the same as below, unless otherwise indicated). **c** A comparison of the binding modes of **14a** and **14b** in SARS-CoV-2 M^pro^ substrate-binding pocket. The major difference between both compounds is marked with a dashed rounded rectangle. **d** Close-up view of **14a** binding pocket. Four subsites, S1′, S1, S2, and S4, are labeled. The polder electron density map of **14a** is colored in blue mesh and contoured at 3σ. **e** The 2*F_o_-F_c_* electron density map for residues involved in **14a** binding is shown as light gray mesh and contoured at 1σ. **f** The schematic diagram of SARS- CoV-2 M^pro^-**14a** interactions shown in **(e)**. **g** Close-up view of **14b** binding pocket. Four subsites, S1′, S1, S2, and S4, are labeled. The polder electron density map of **14b** is colored in blue mesh and contoured at 3σ. **h** The 2*F_o_-F_c_* electron density map for residues involved in **14b** binding is shown as light gray mesh and contoured at 1σ. **i** Schematic diagram of SARS-CoV-2 M^pro^-**14b** interactions shown in **(h)**.


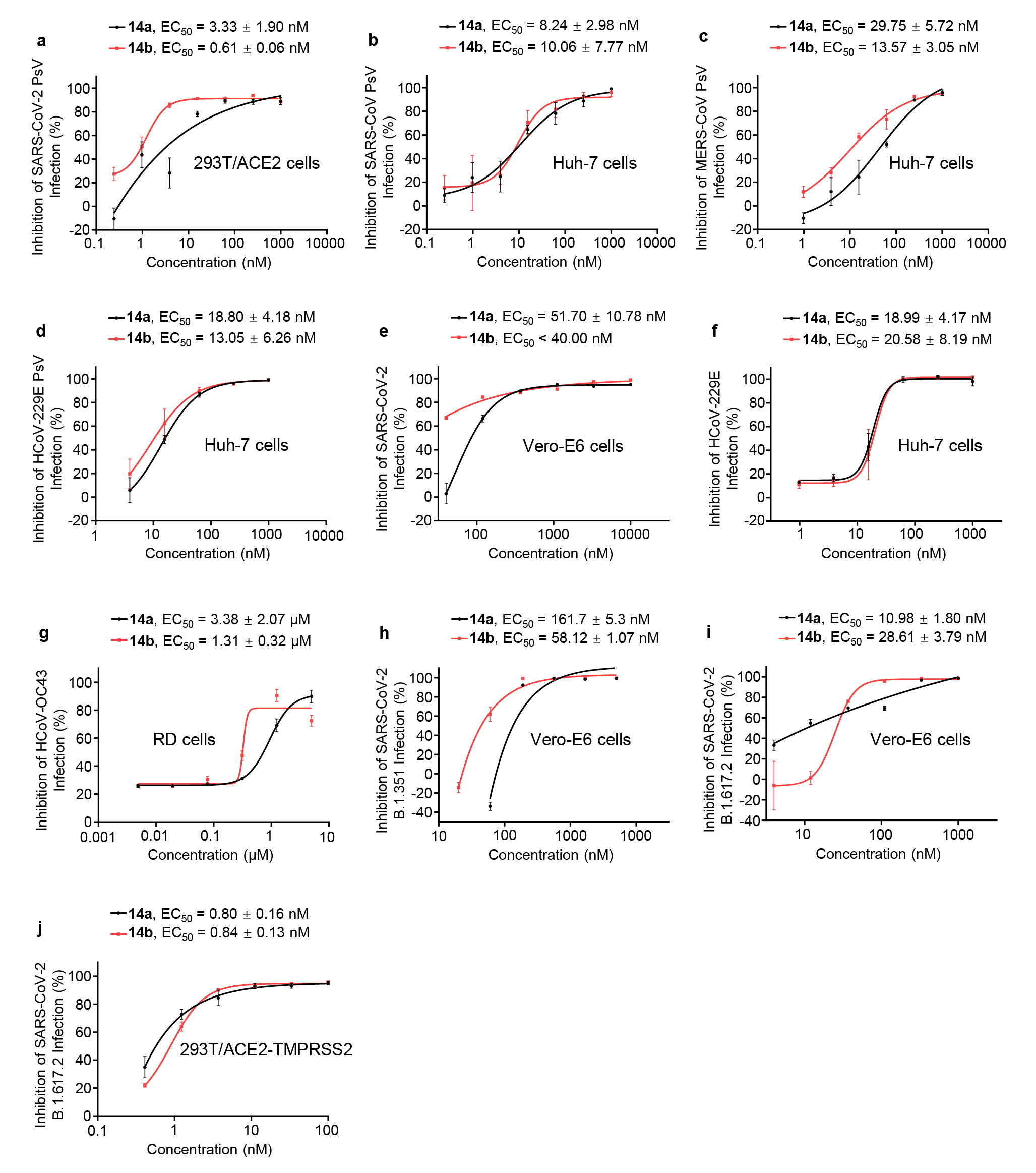


**Fig. S5 Broad human coronavirus inhibitory activity of 14a and 14b.** Inhibition of **14a** and **14b** against SARS-CoV-2 PsV **(a)** infection in 293T/ACE2 cells, SARS-CoV PsV **(b)**, MERS-CoV PsV **(c)**, HCoV-229E PsV **(d)** infection in Huh-7 cells, authentic SARS-CoV-2 infection in Vero-E6 cells **(e)**, authentic HCoV-229E infection in Huh-7 cells **(f)**, authentic HCoV-OC43 infection in RD cells **(g)**, authentic SARS-CoV-2 B.1.351 (Beta) variant infection in Vero-E6 cells **(h)**, authentic SARS-CoV-2 B.1.617.2 (Delta) variant infection in Vero-E6 cells **(i)** and ACE2-TMPRSS2-expressing 293T cells **(j)**. Each sample was tested in triplicate, and the experiment was repeated twice. Data from a representative experiment are shown as mean ± SD.

**
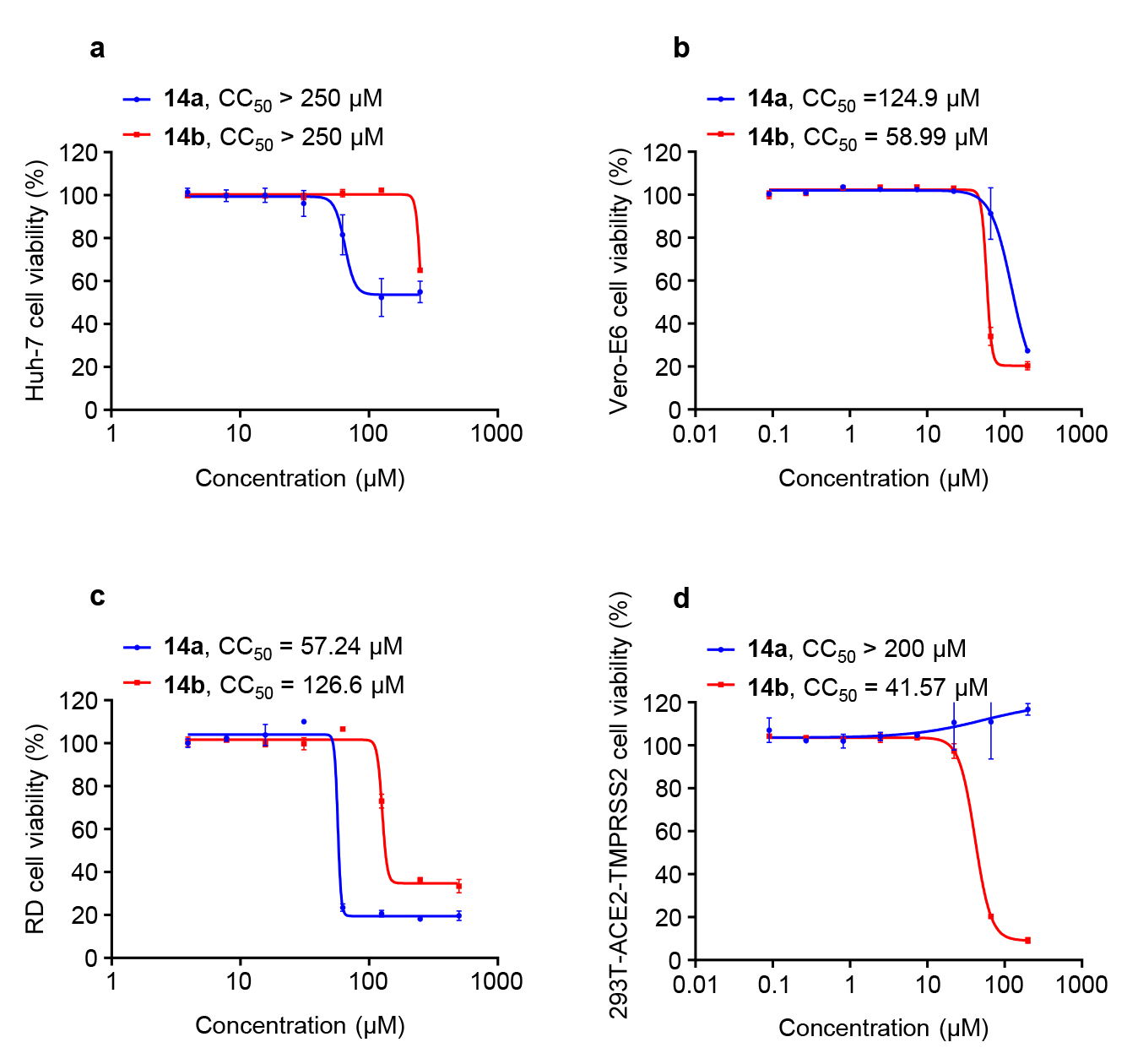
**

**Fig. S6 Cytotoxicity of compounds 14a and 14b.** The cytotoxicity of compounds **14a** and **14b** on cell lines of Huh-7 (**a**), Vero-E6 (**b**), RD (**c**) and 293T-ACE2-TMPRSS2 (**d**). Each sample was tested in duplicate and the data are shown as mean.

**
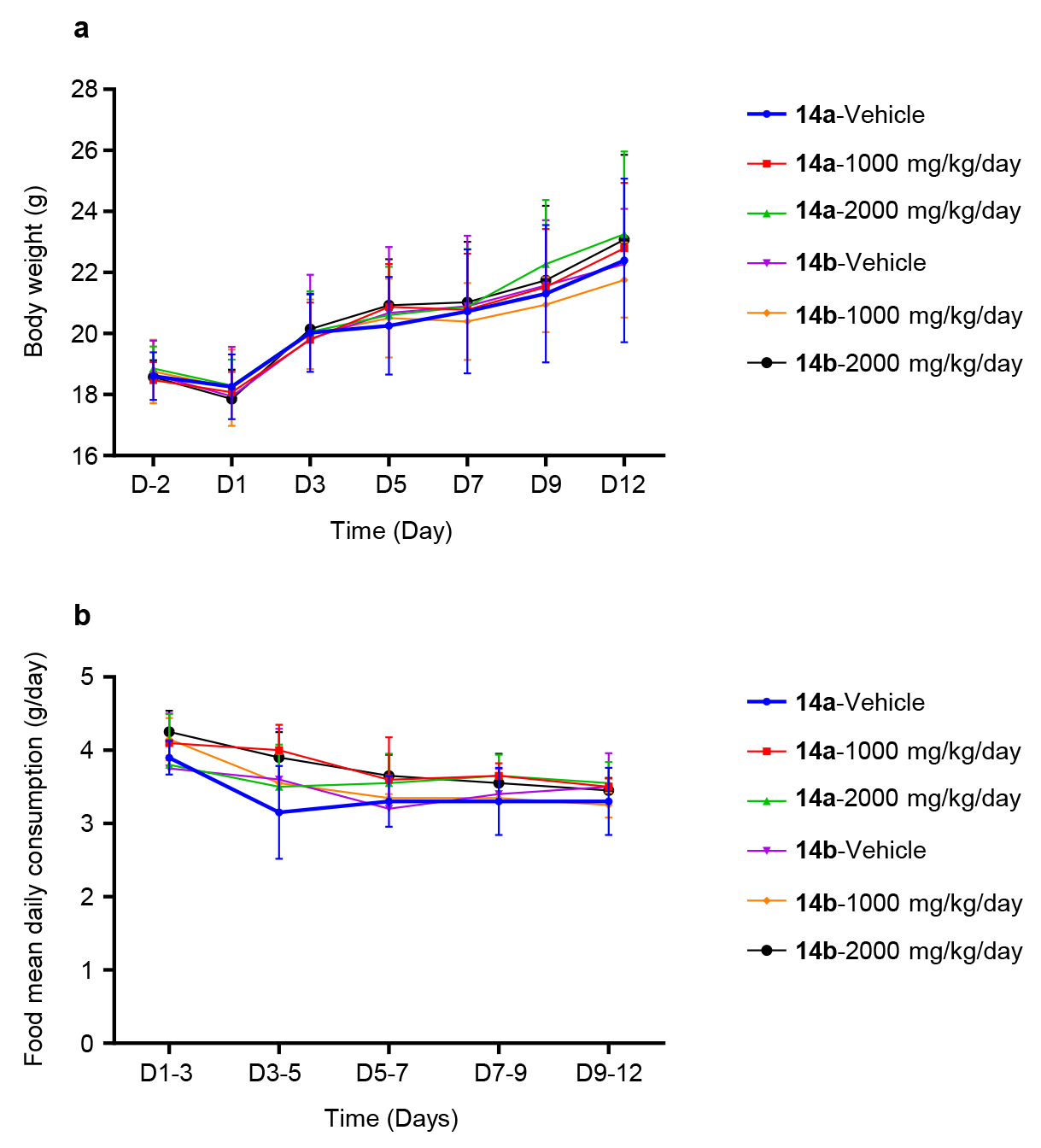
**

**Fig. S7 *In vivo* toxicity studies of compounds 14a and 14b. a** Body weight changes of mice. **b** Food mead daily consumption of mice.

**Fig. S8 Spectral data for all target compounds.**

**^1^H, ^13^C and ^19^F NMR, HRMS and HPLC spectra of 14a.**

**
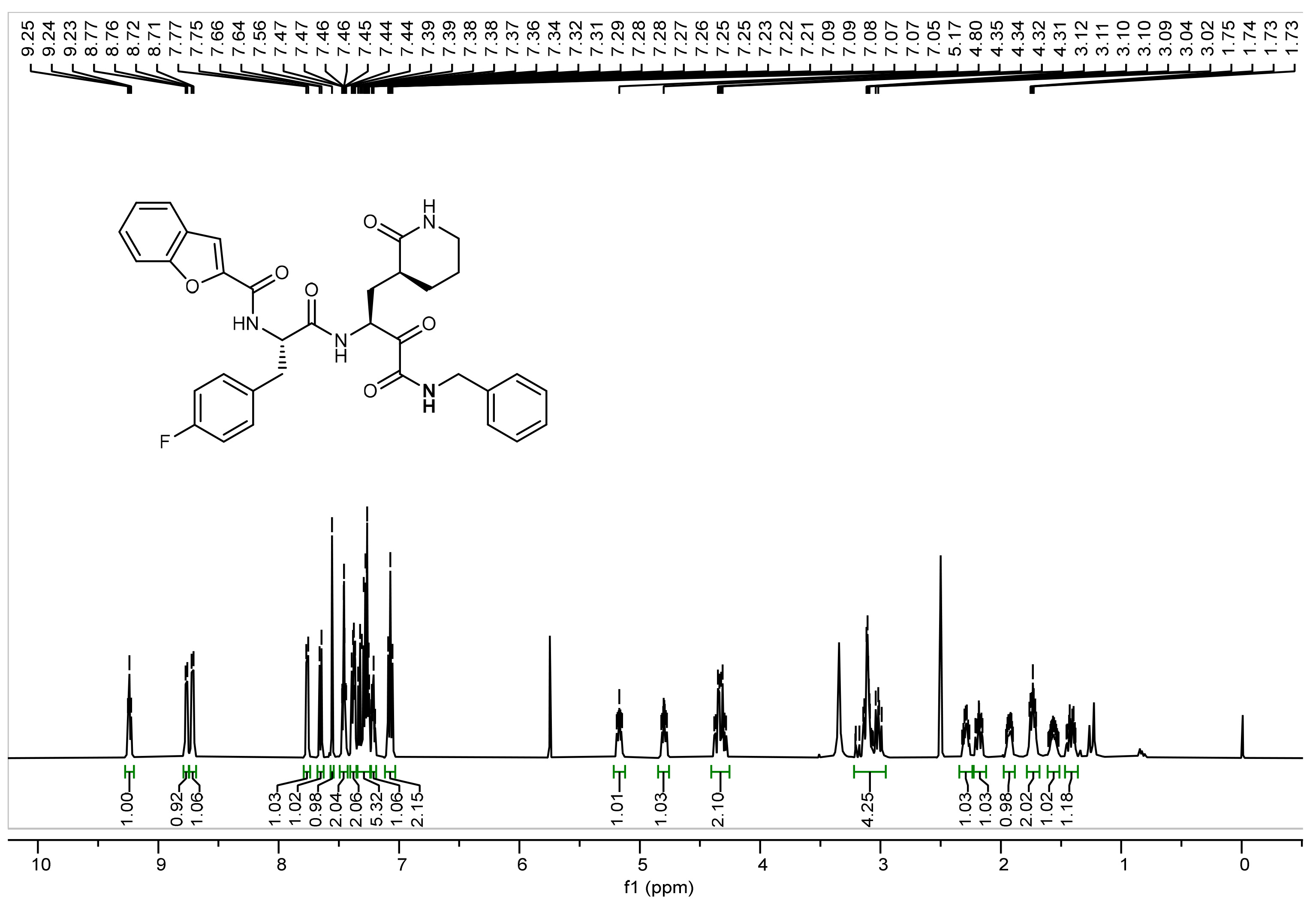

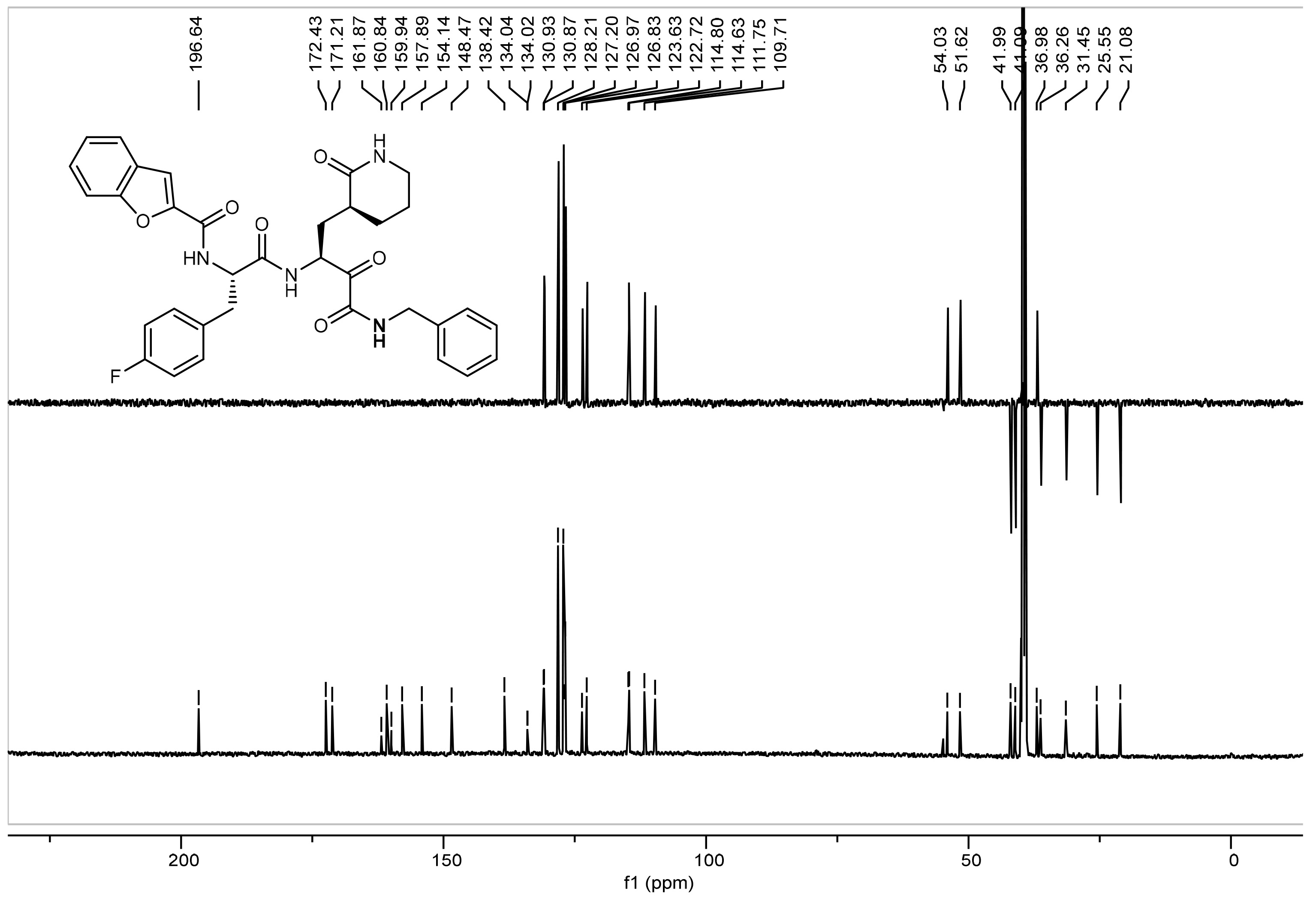
**

**
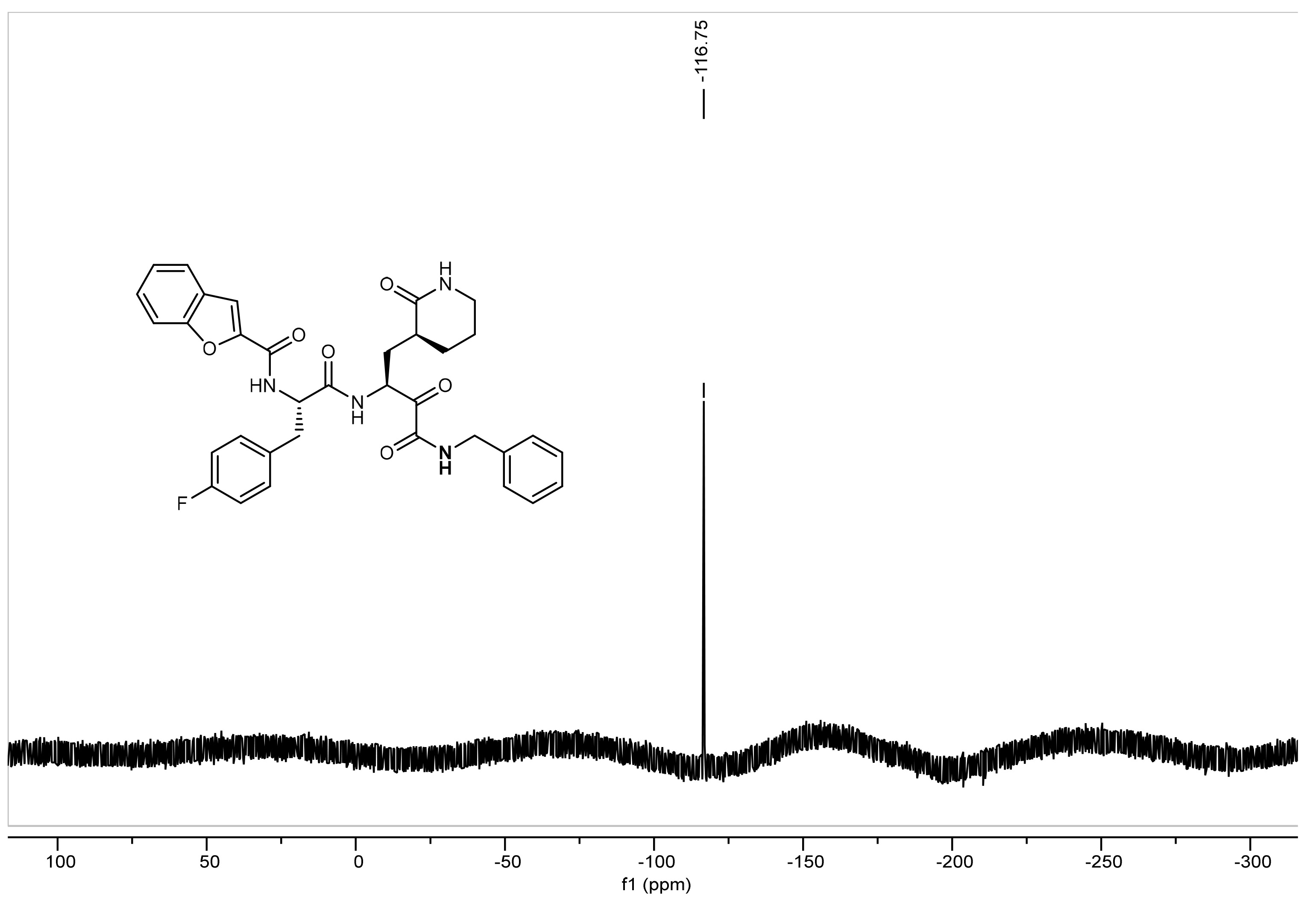
**


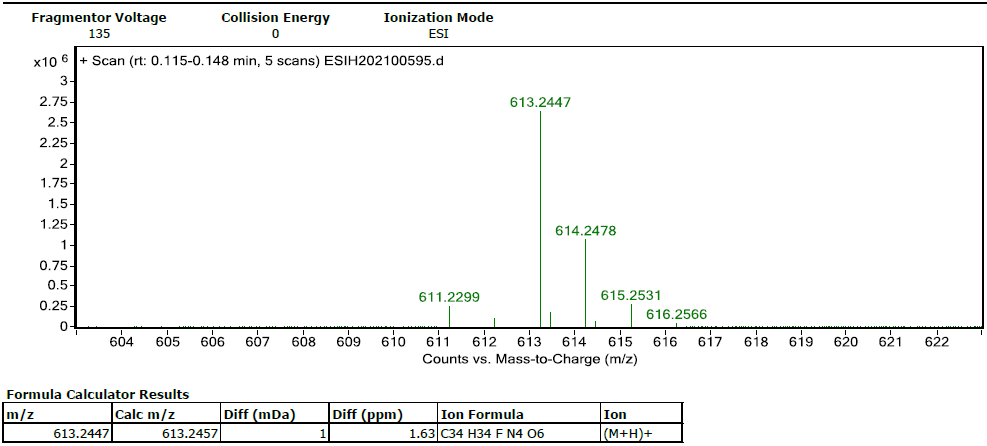


**
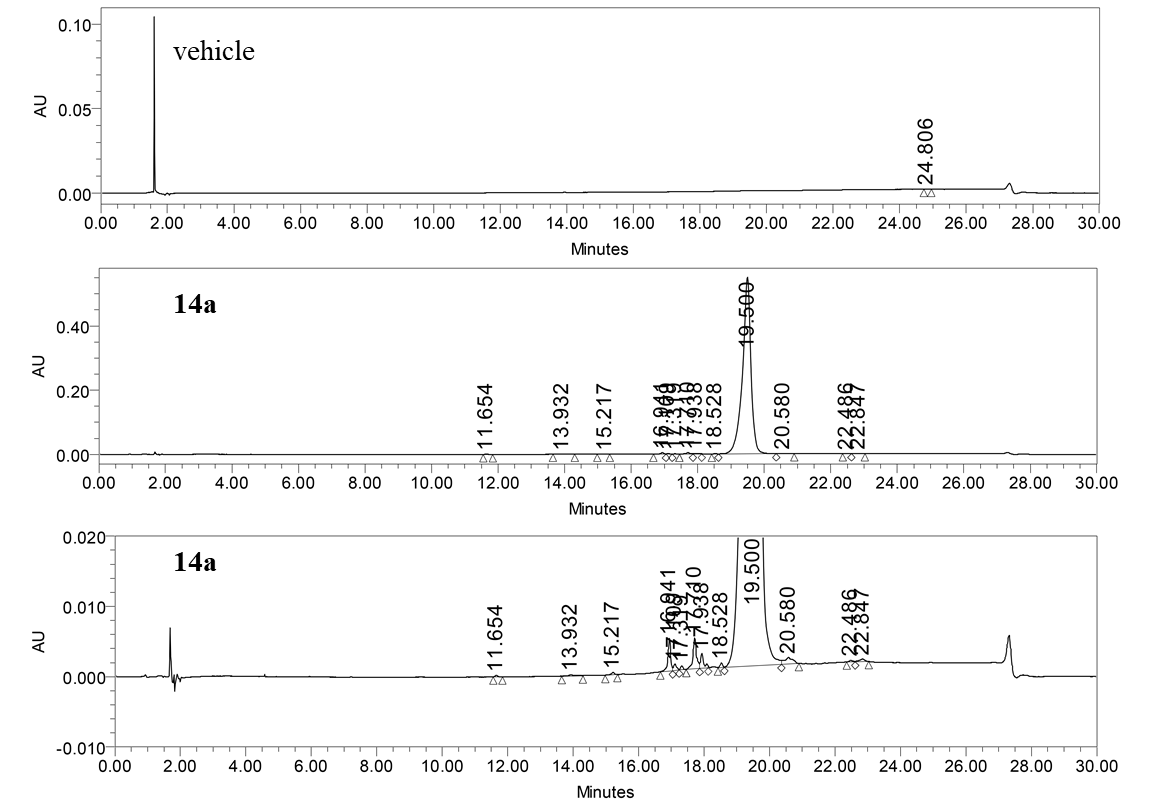
**

**
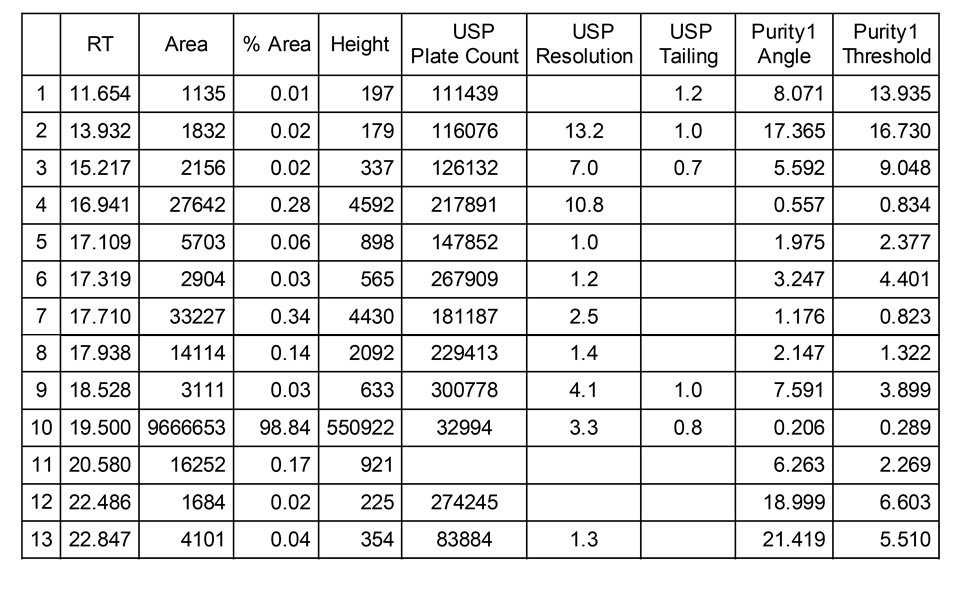
**

**^1^H and ^13^C NMR, HRMS and HPLC spectra of 14b.**

**
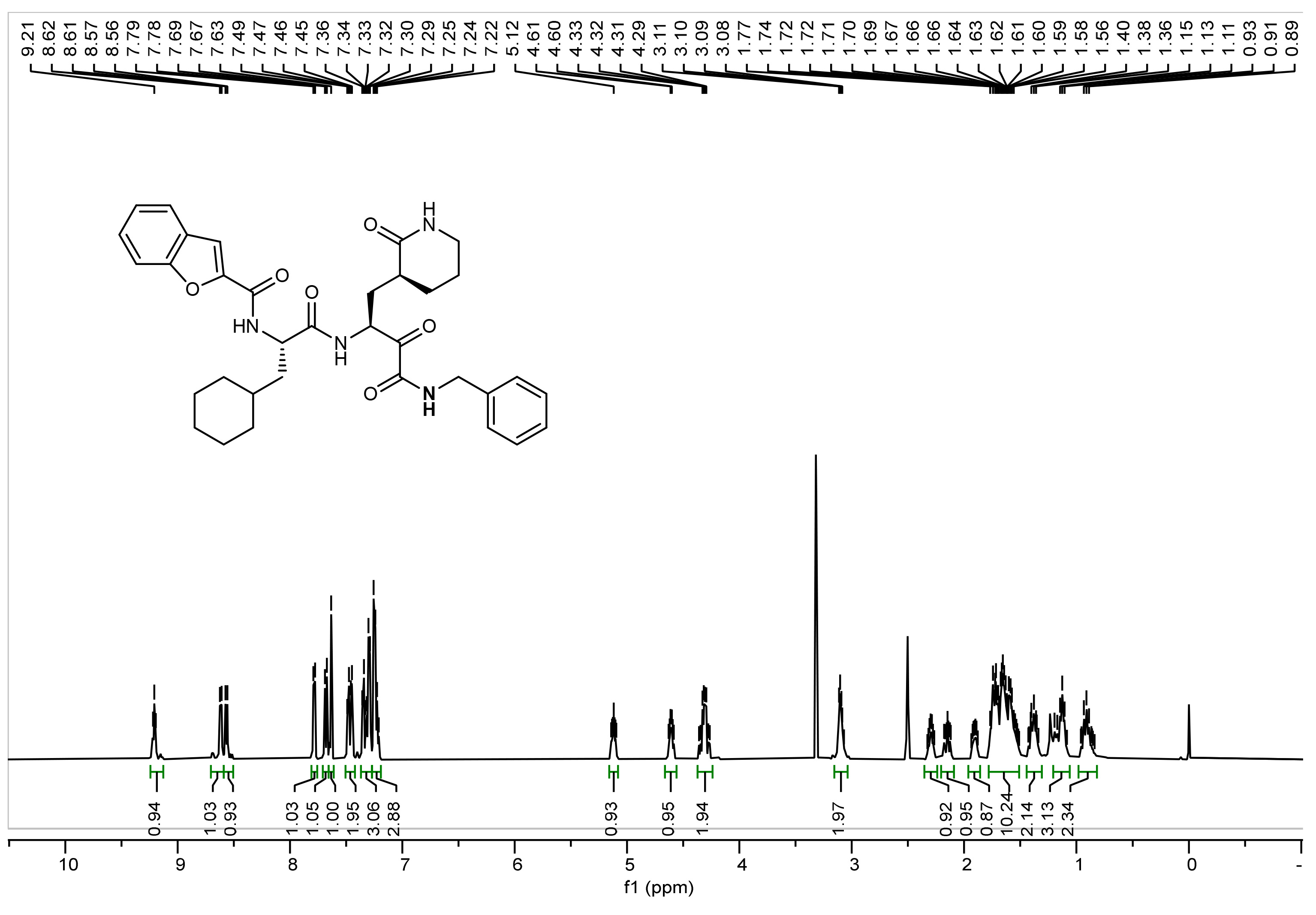
**

**
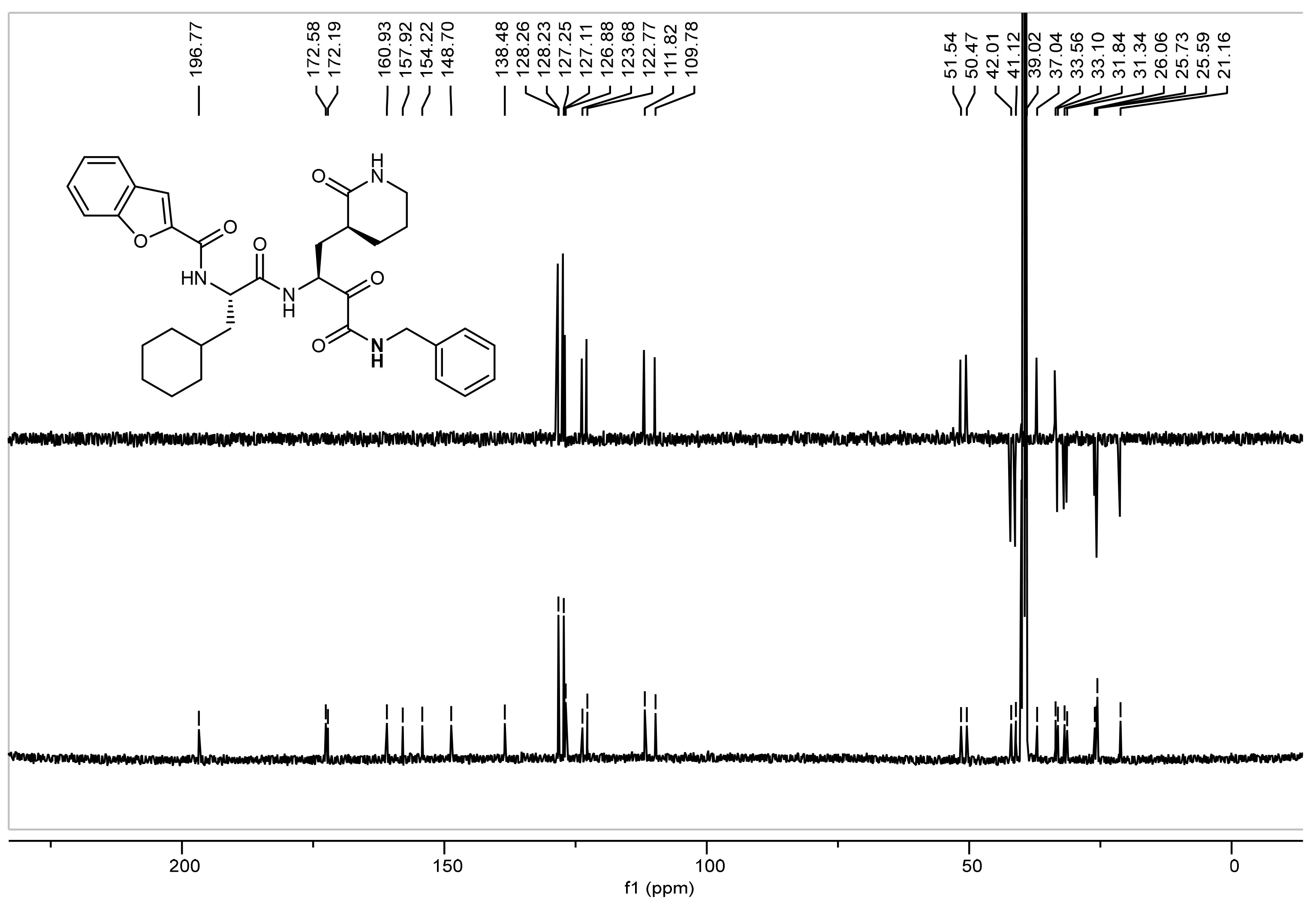
**


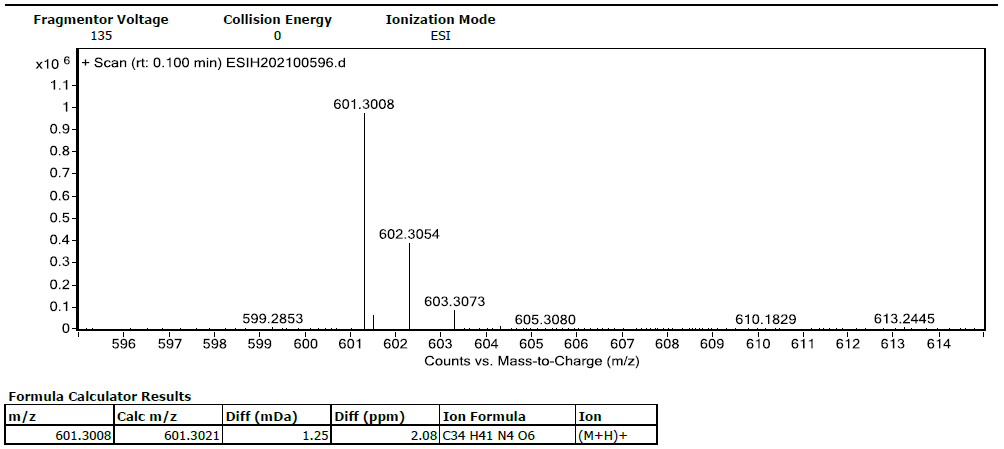


**
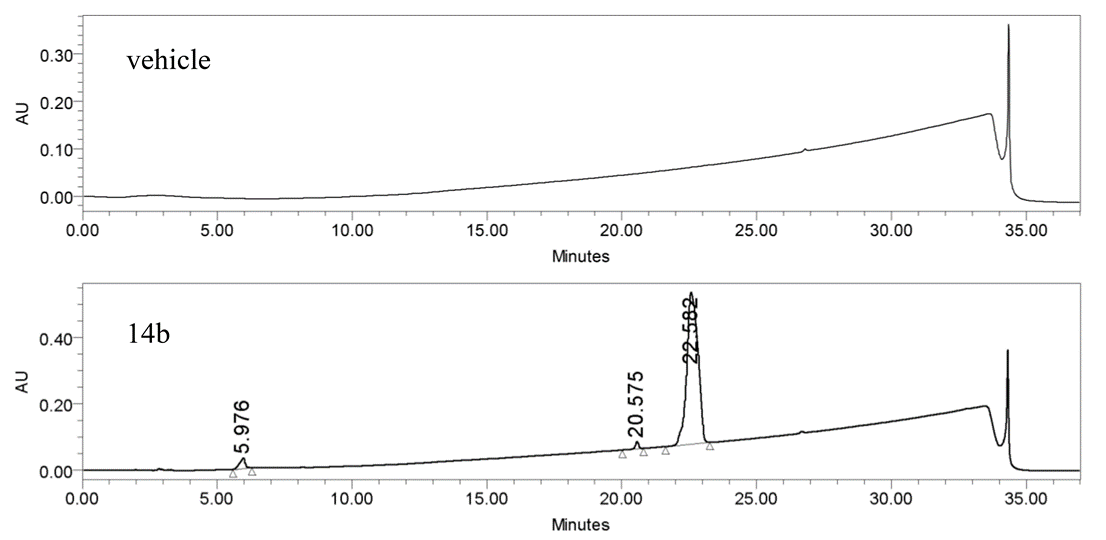
**

**
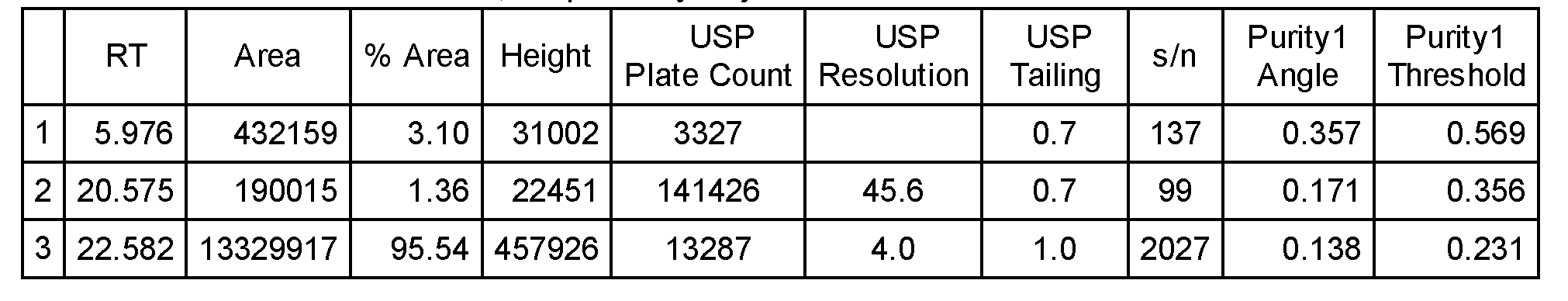
**

**^1^H, ^13^C and ^19^F NMR, HRMS spectra of 14c.**


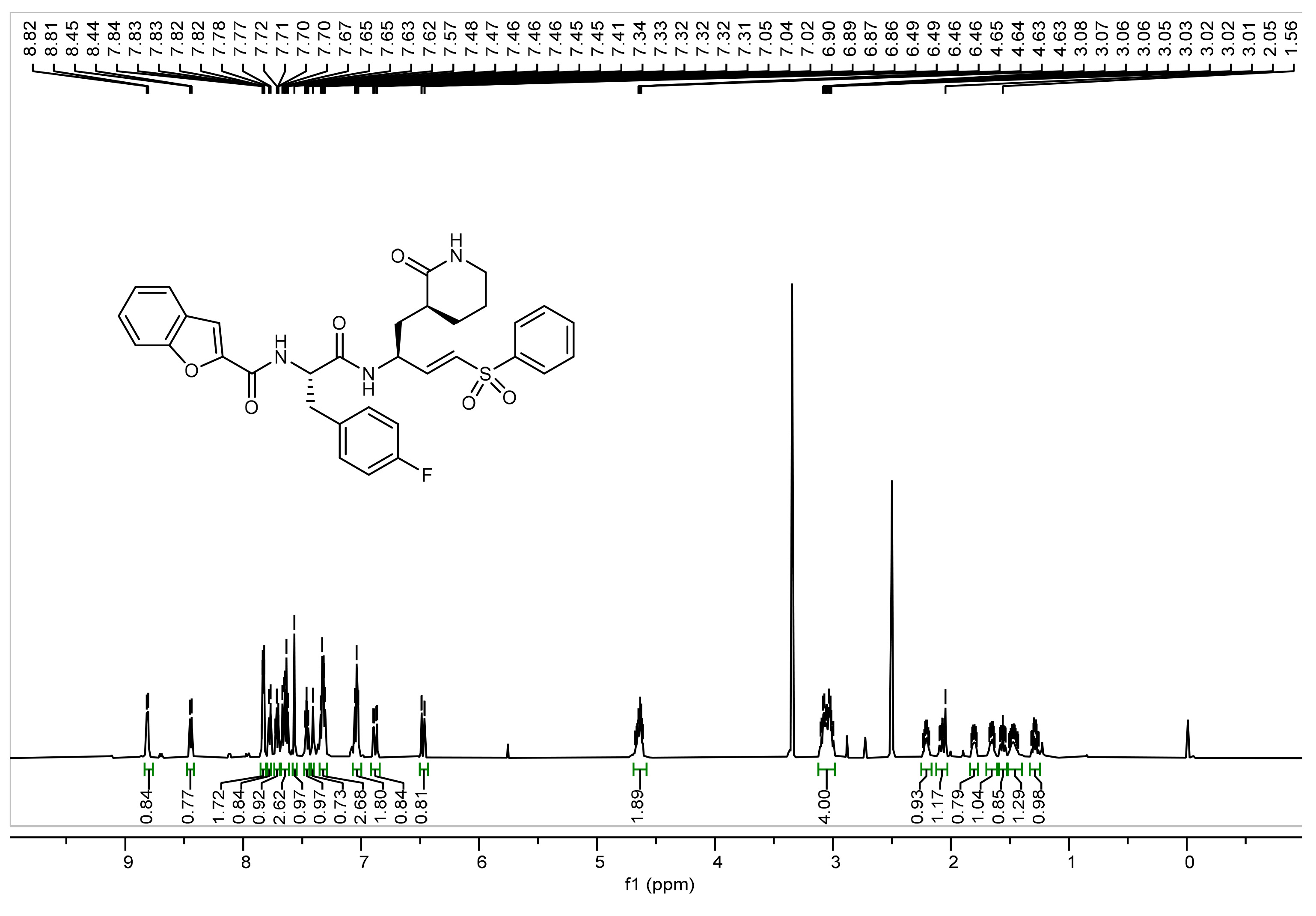


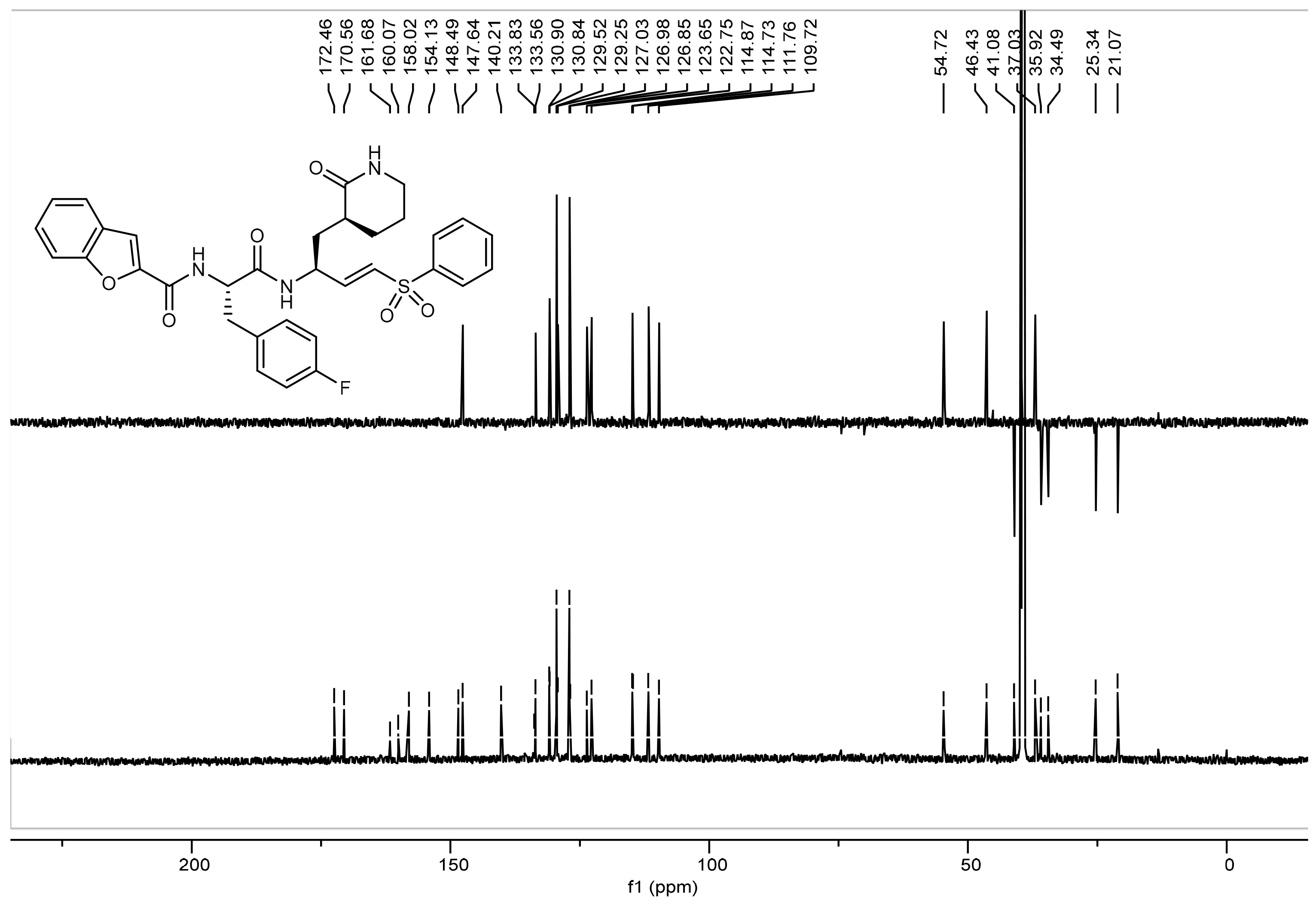


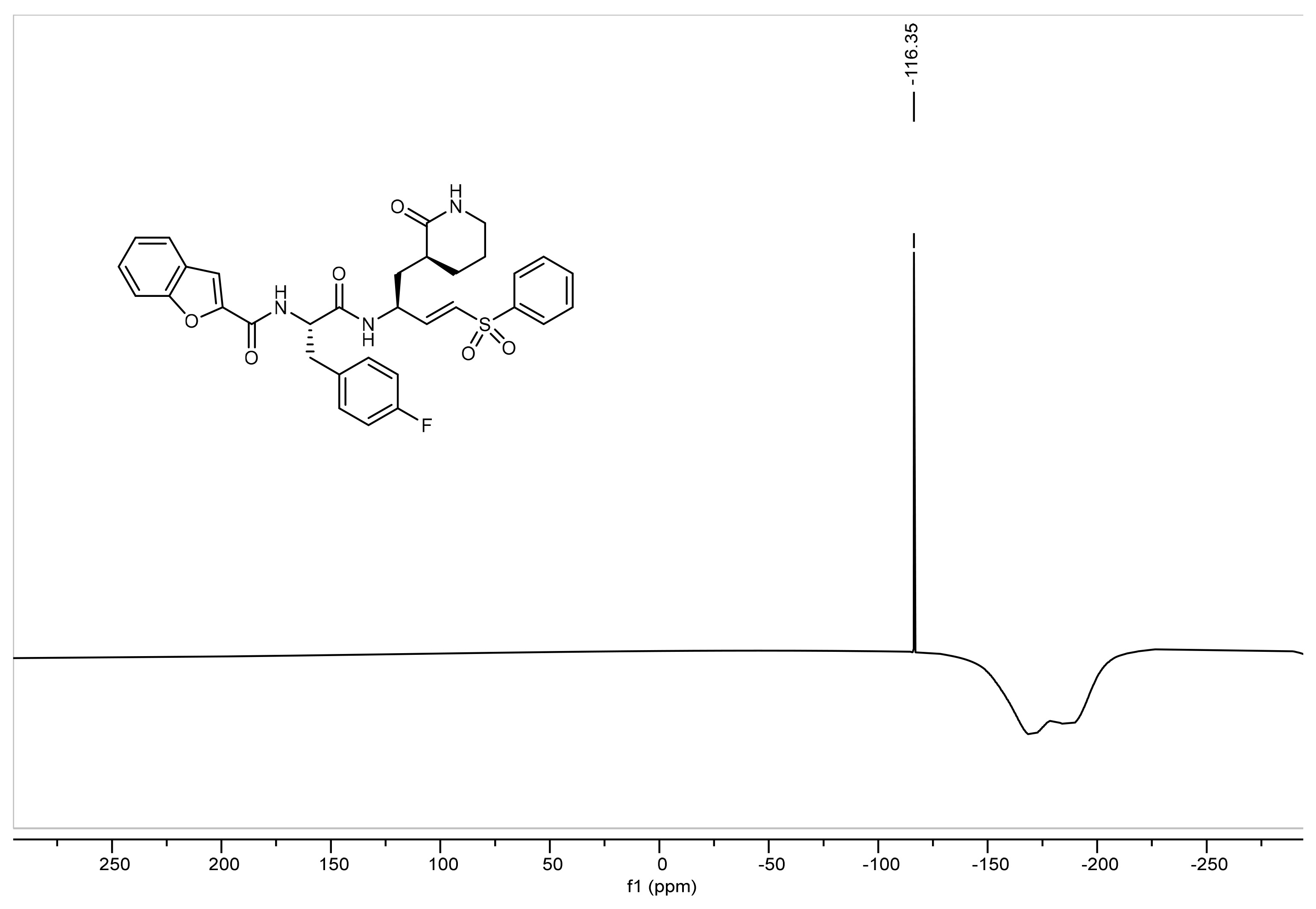


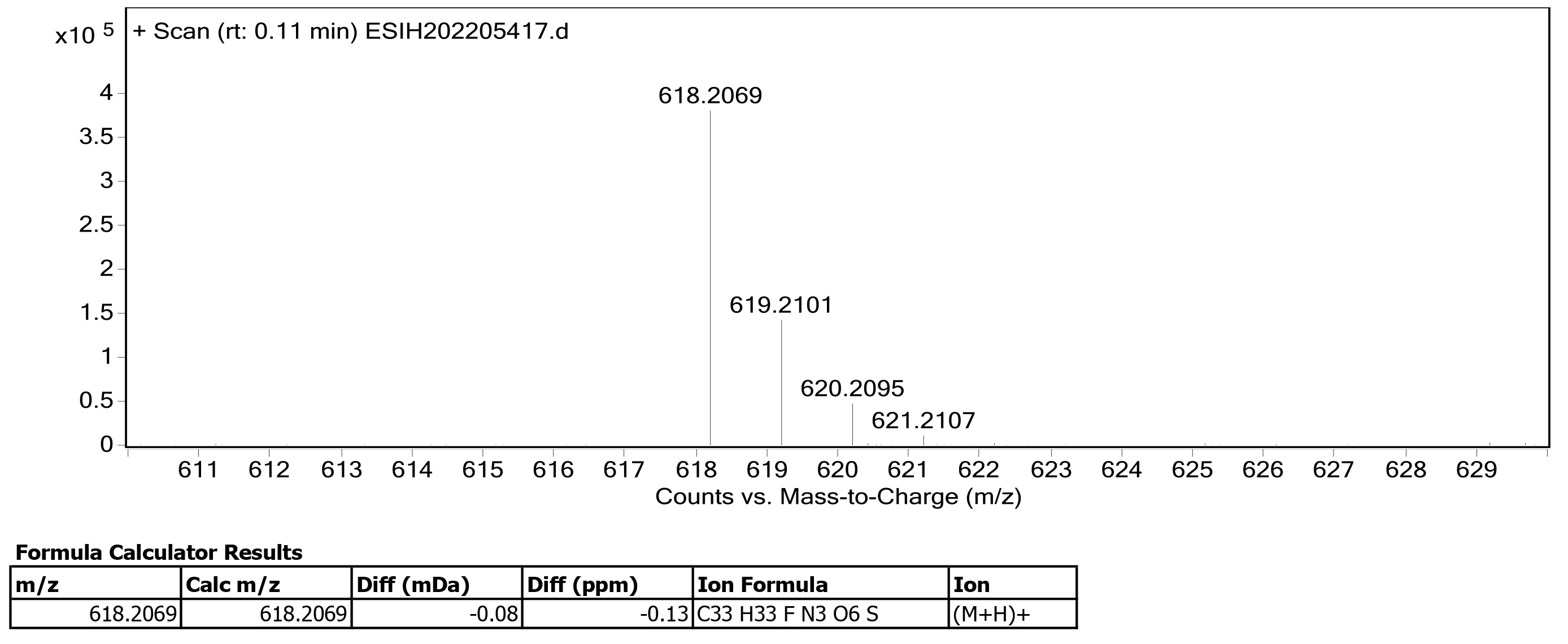


**^1^H and ^13^C NMR, HRMS spectra of 14d.**


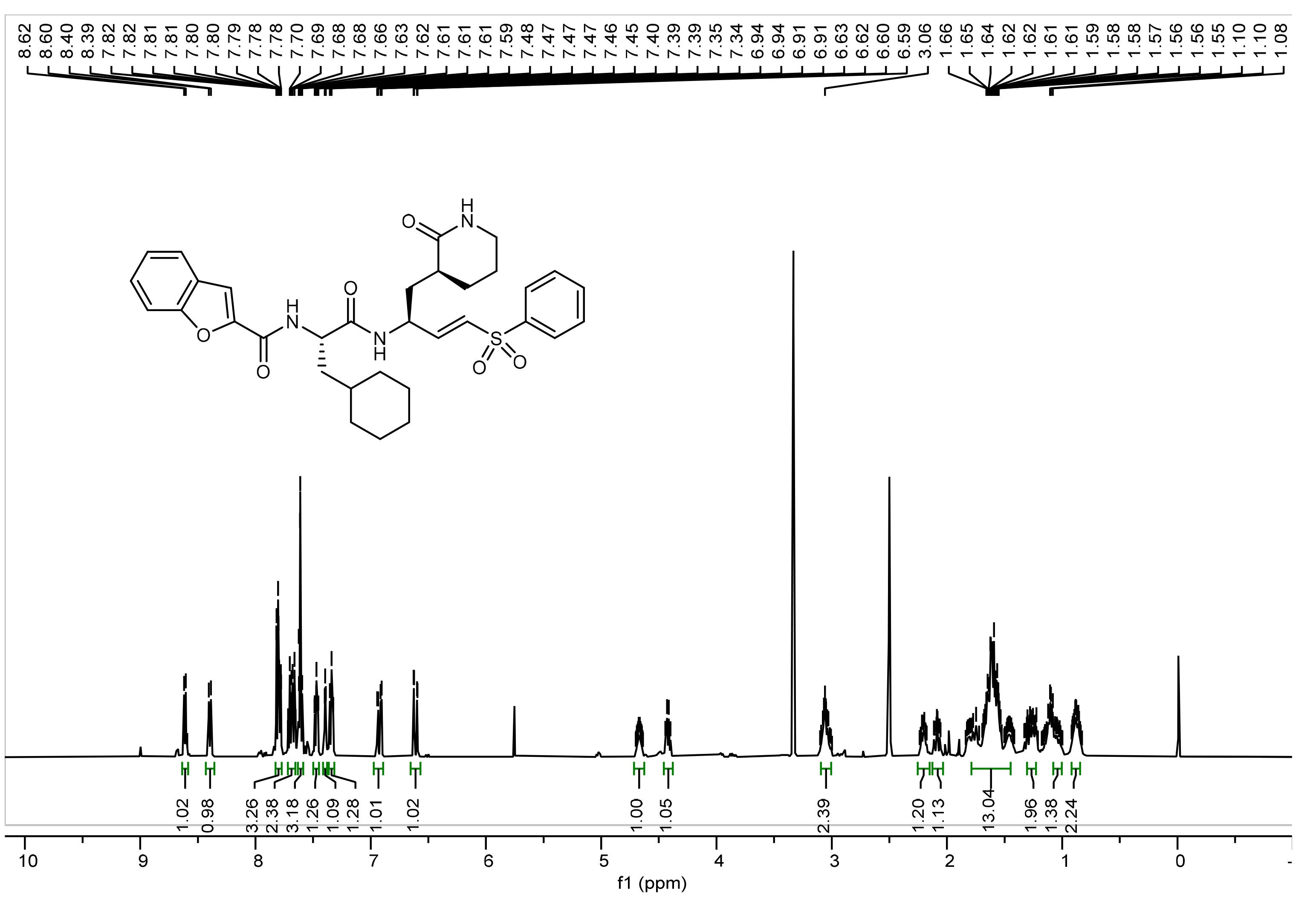


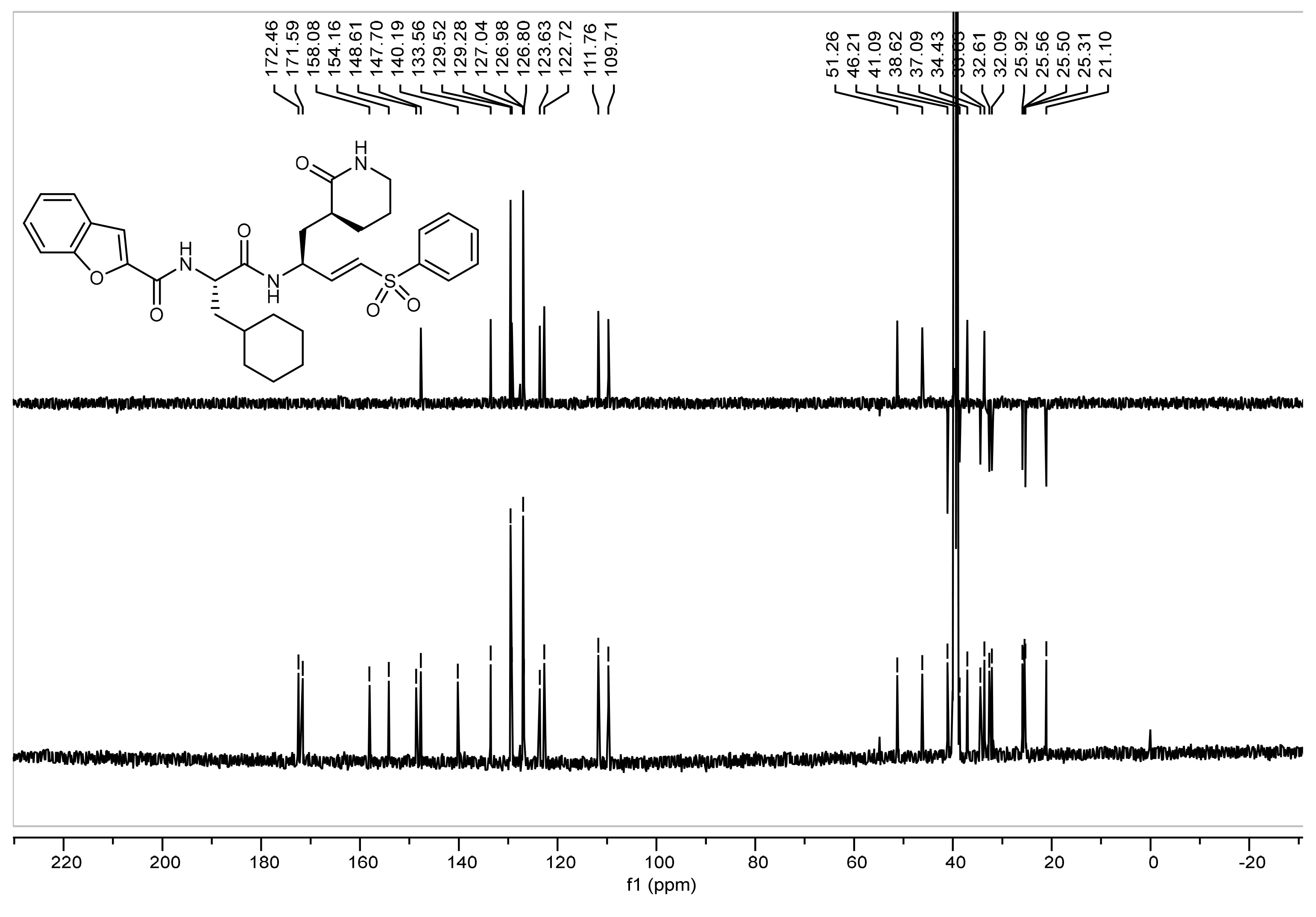


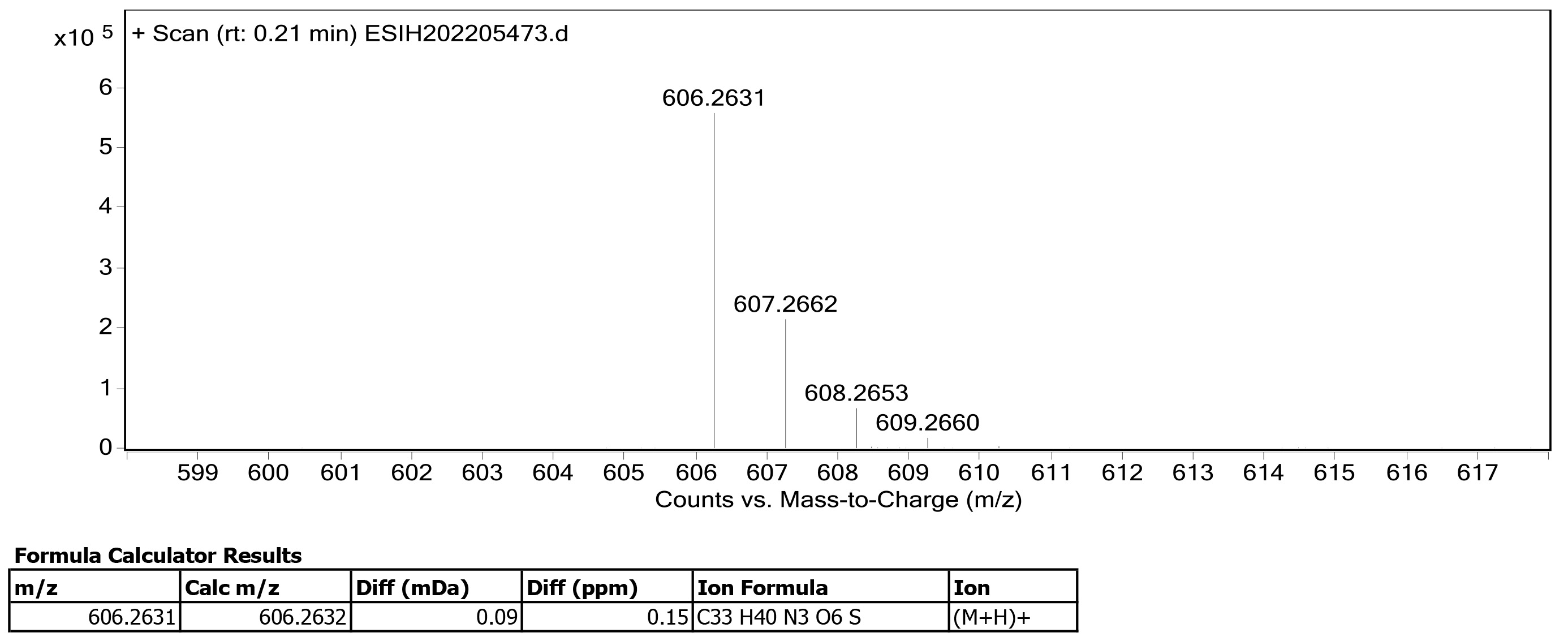


**^1^H, ^13^C and ^19^F NMR, HRMS spectra of 14e.**


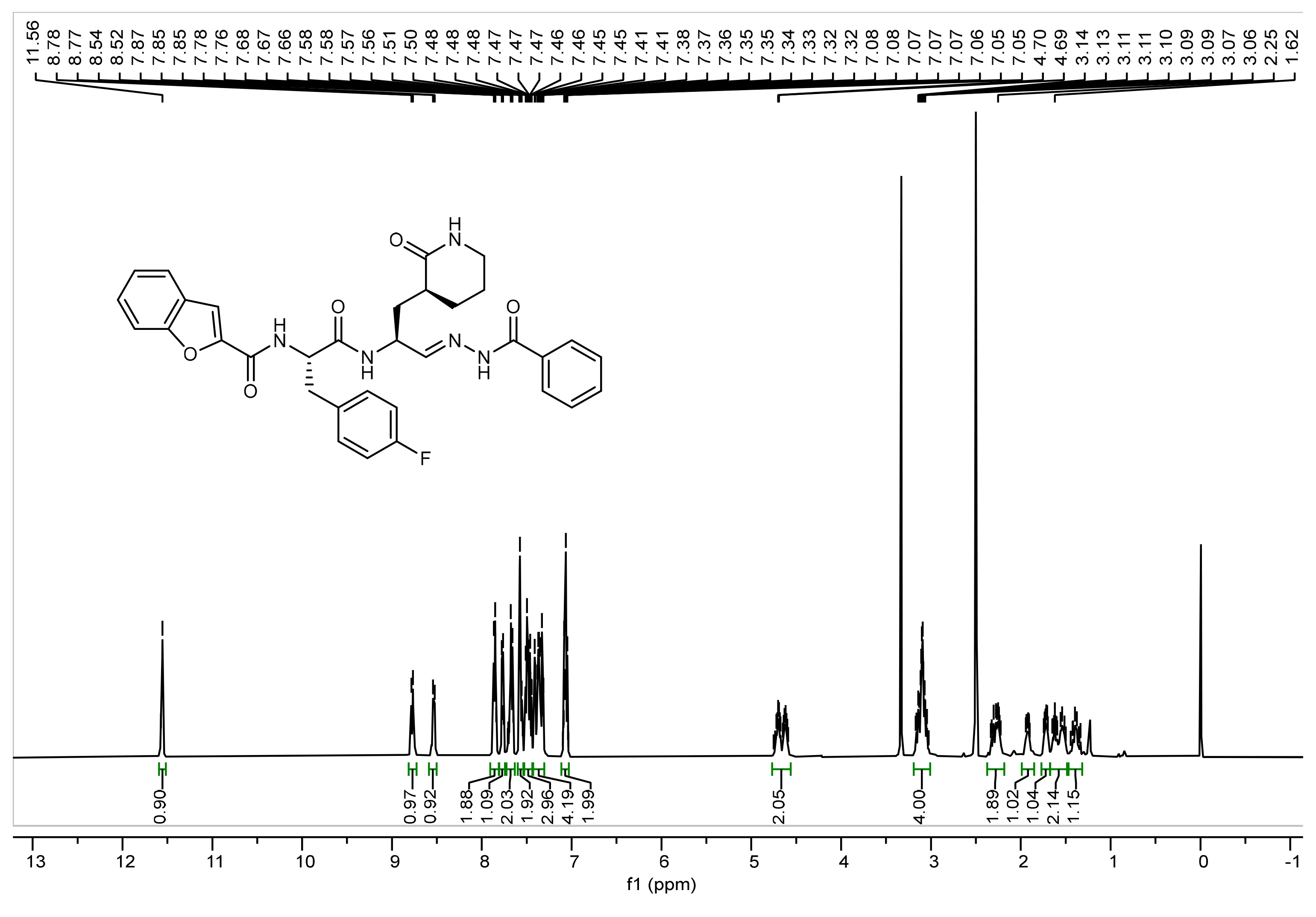


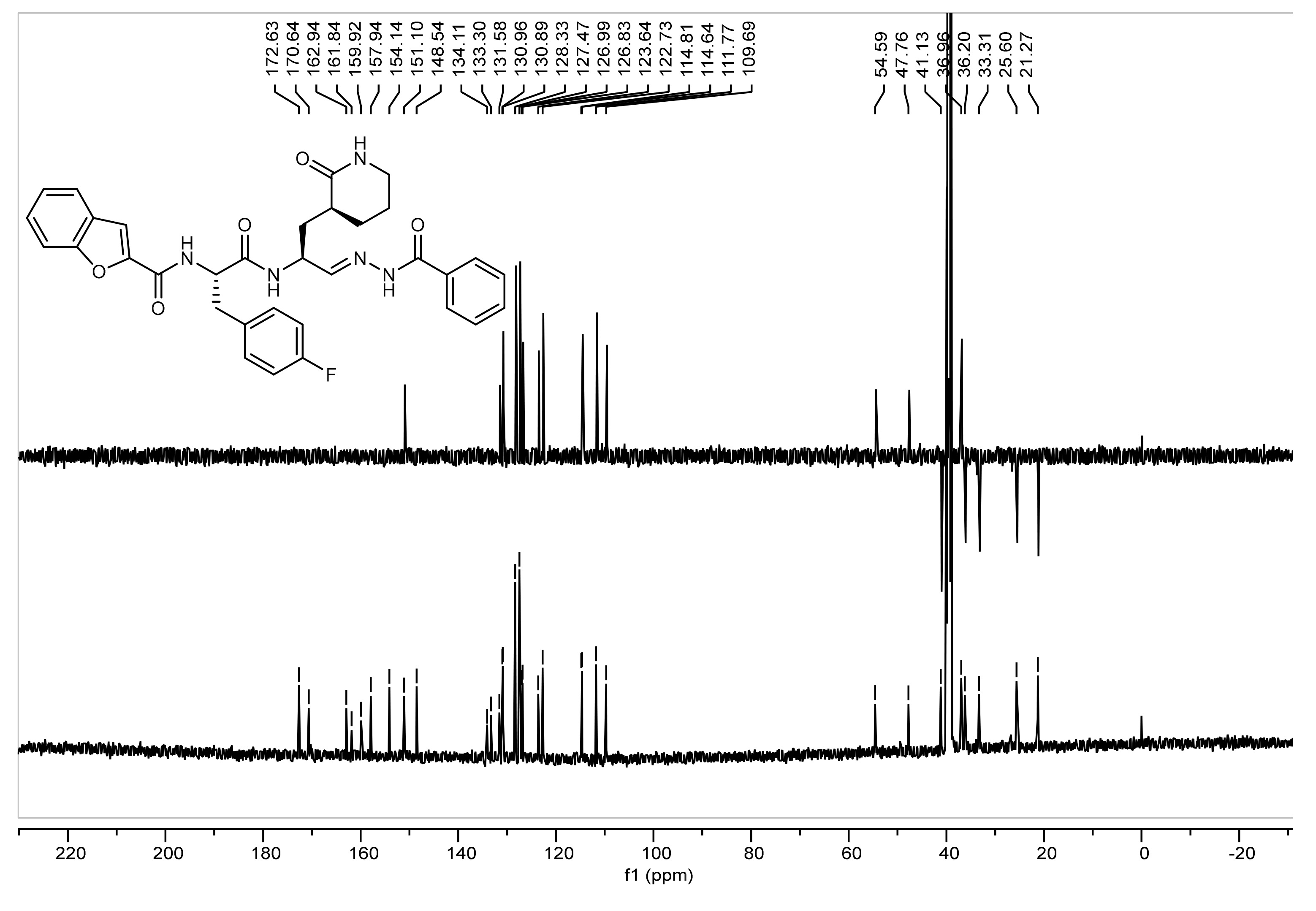


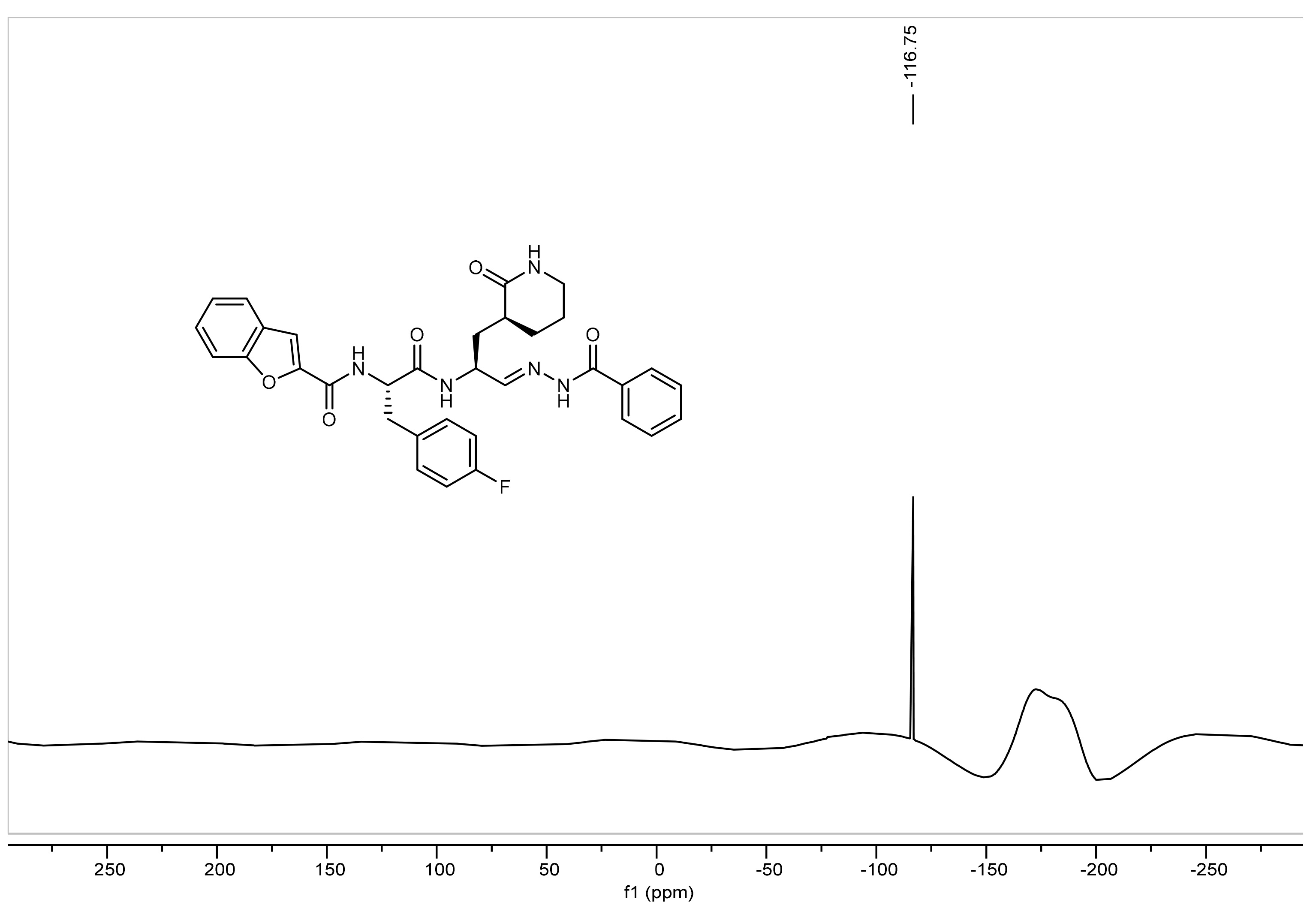


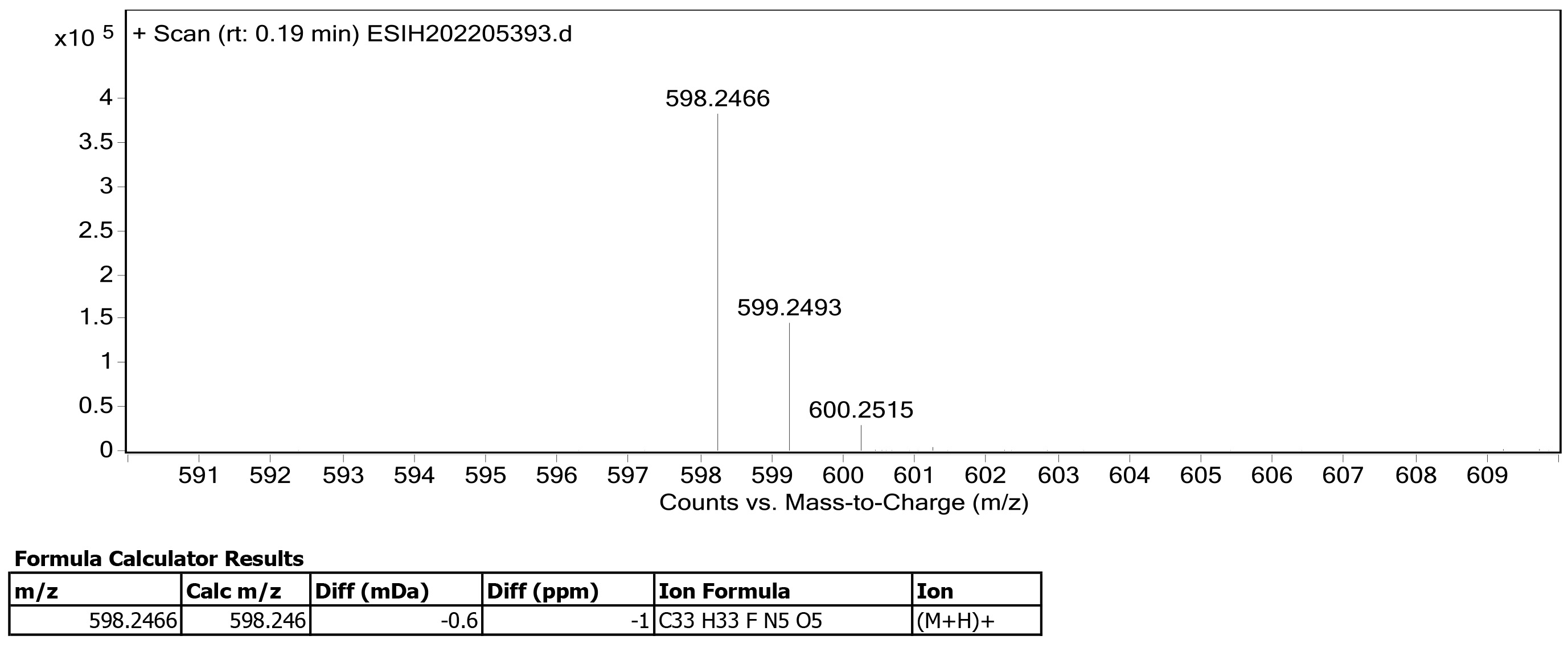


**^1^H, ^13^C and ^19^F NMR, HRMS spectra of 14f.**


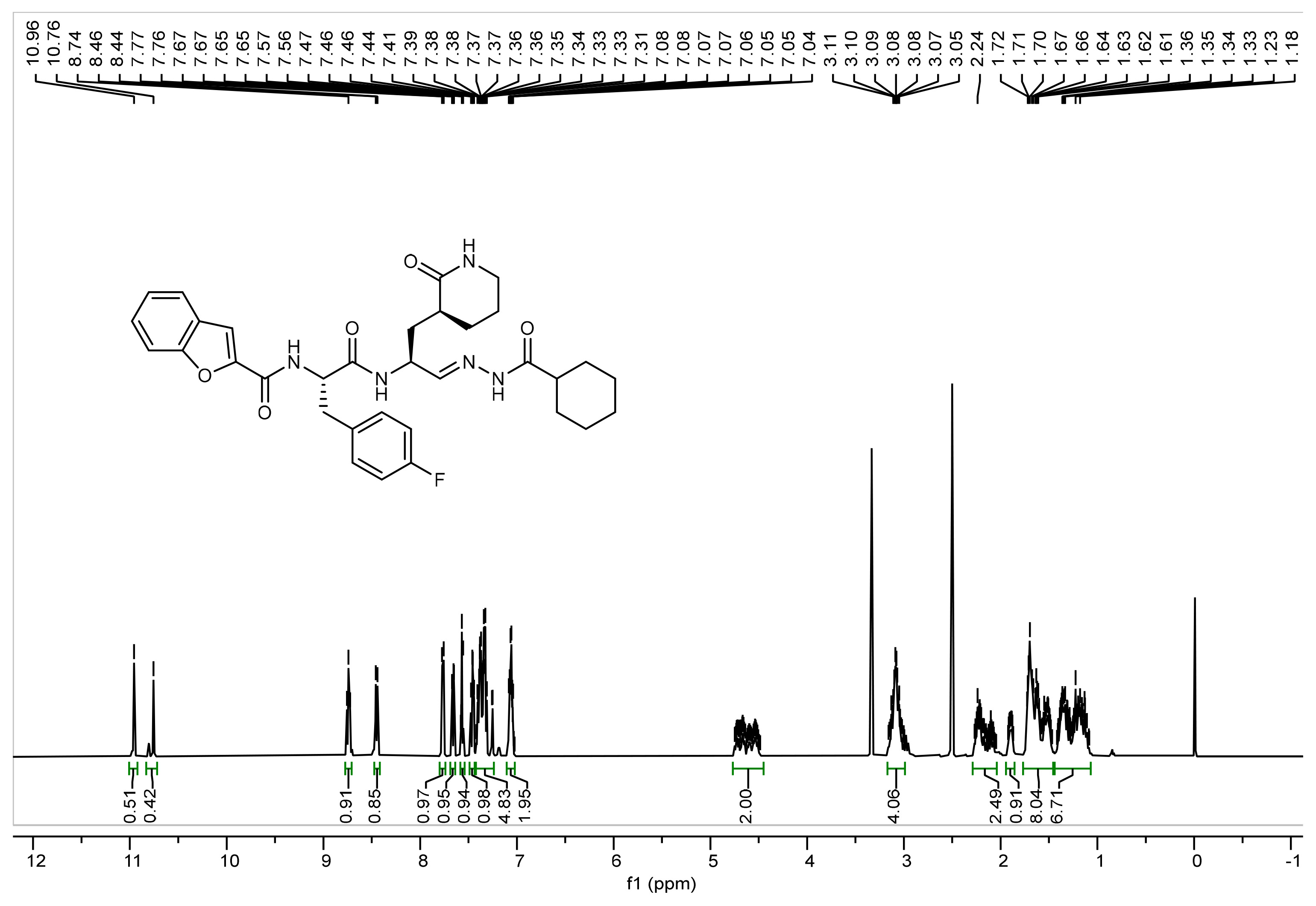


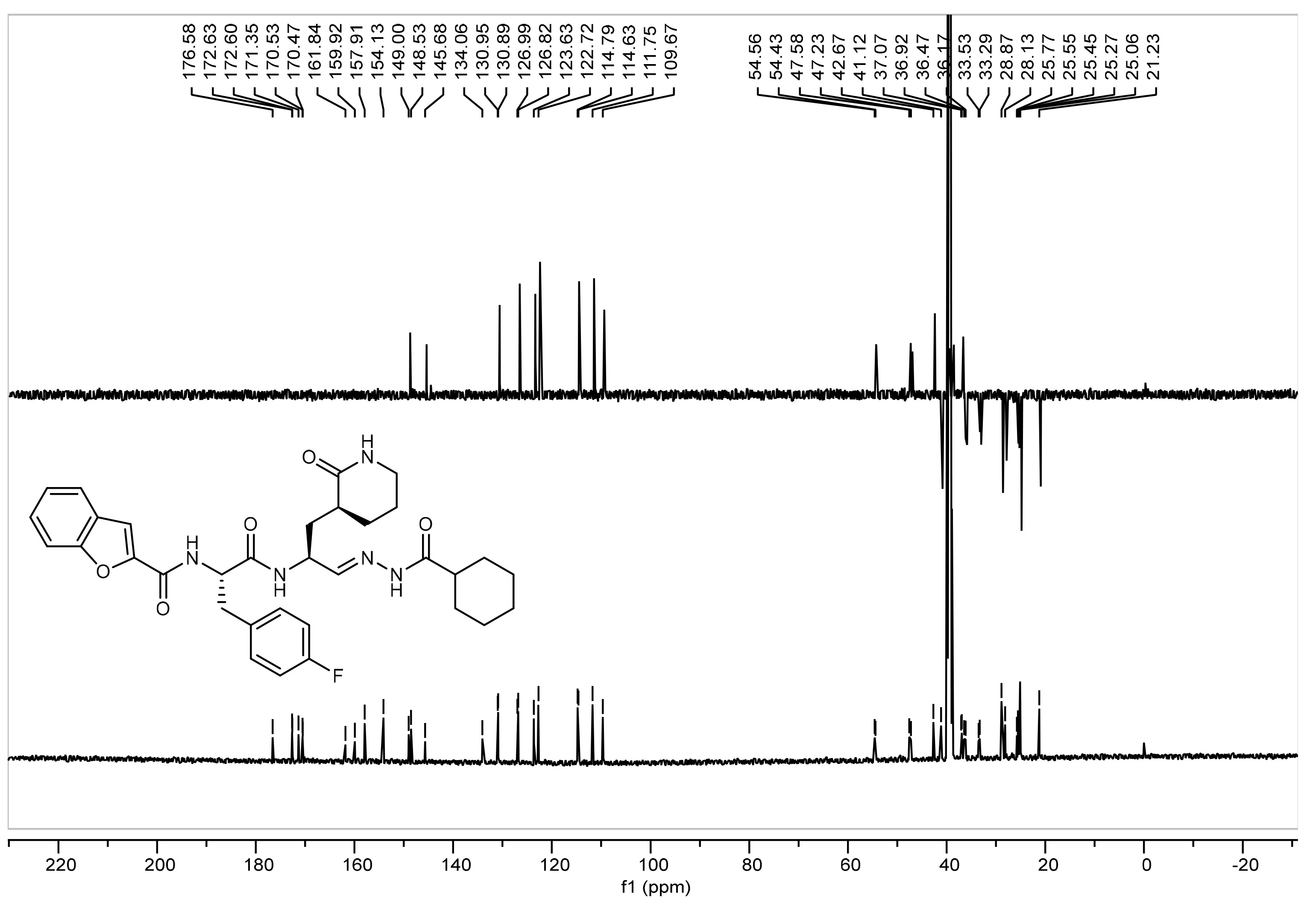


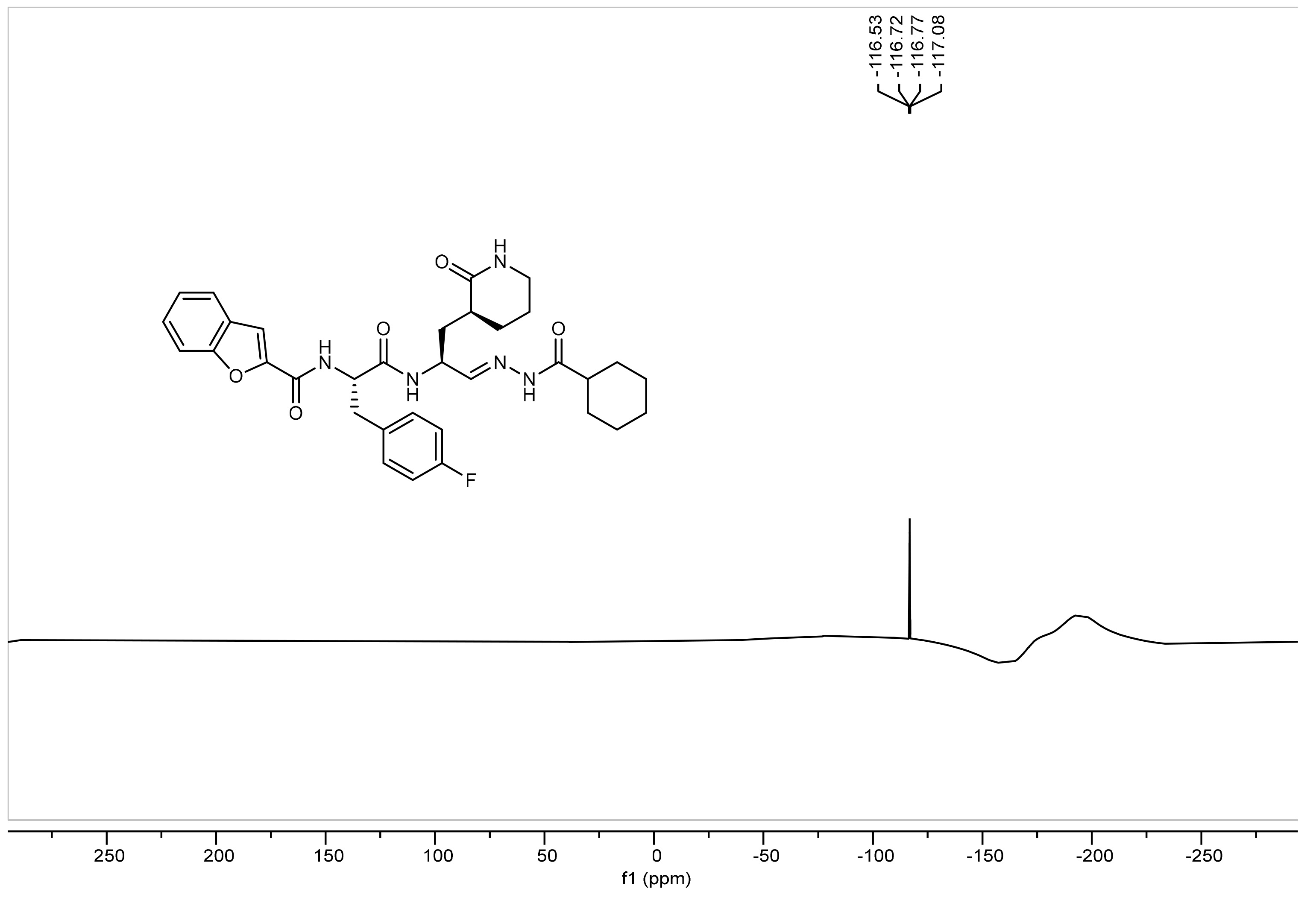


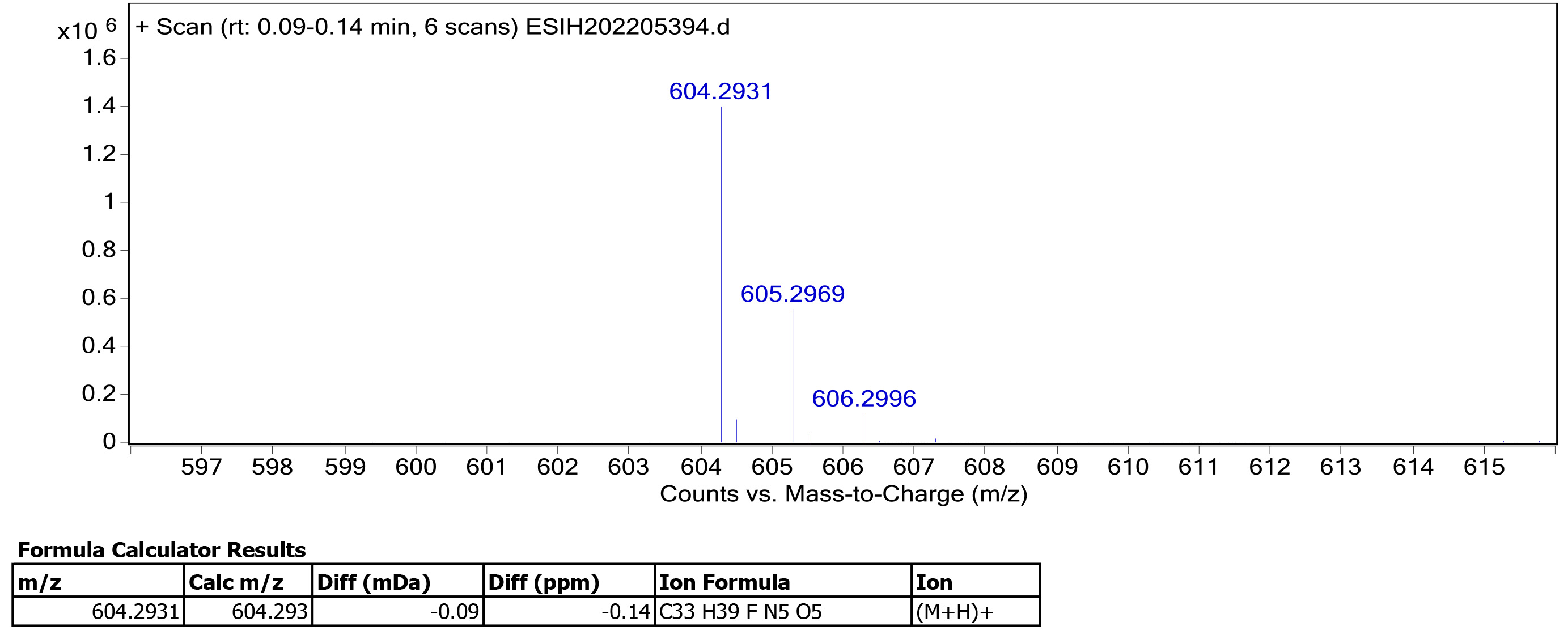


**^1^H, ^13^C and ^19^F NMR, HRMS spectra of 14g.**


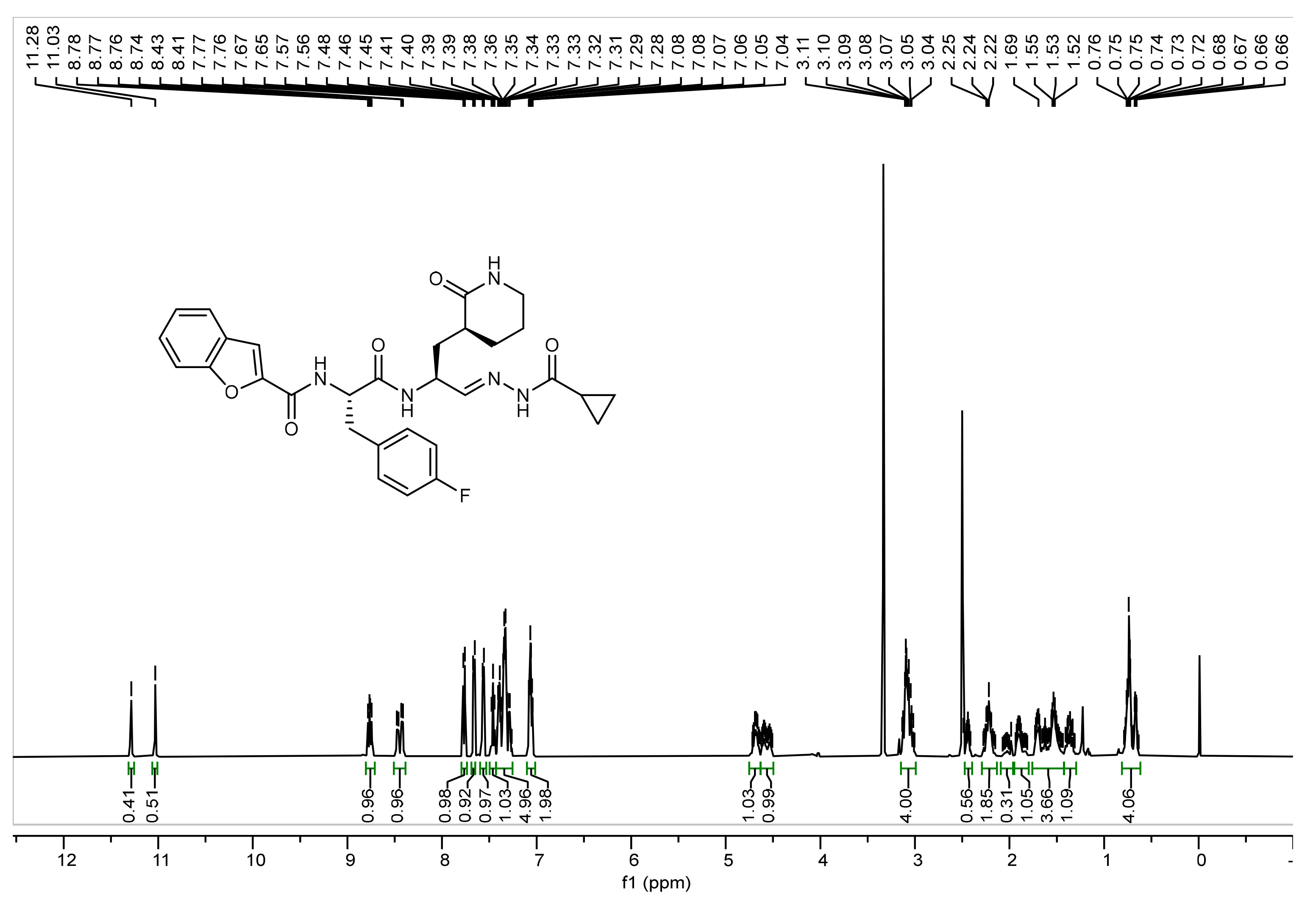


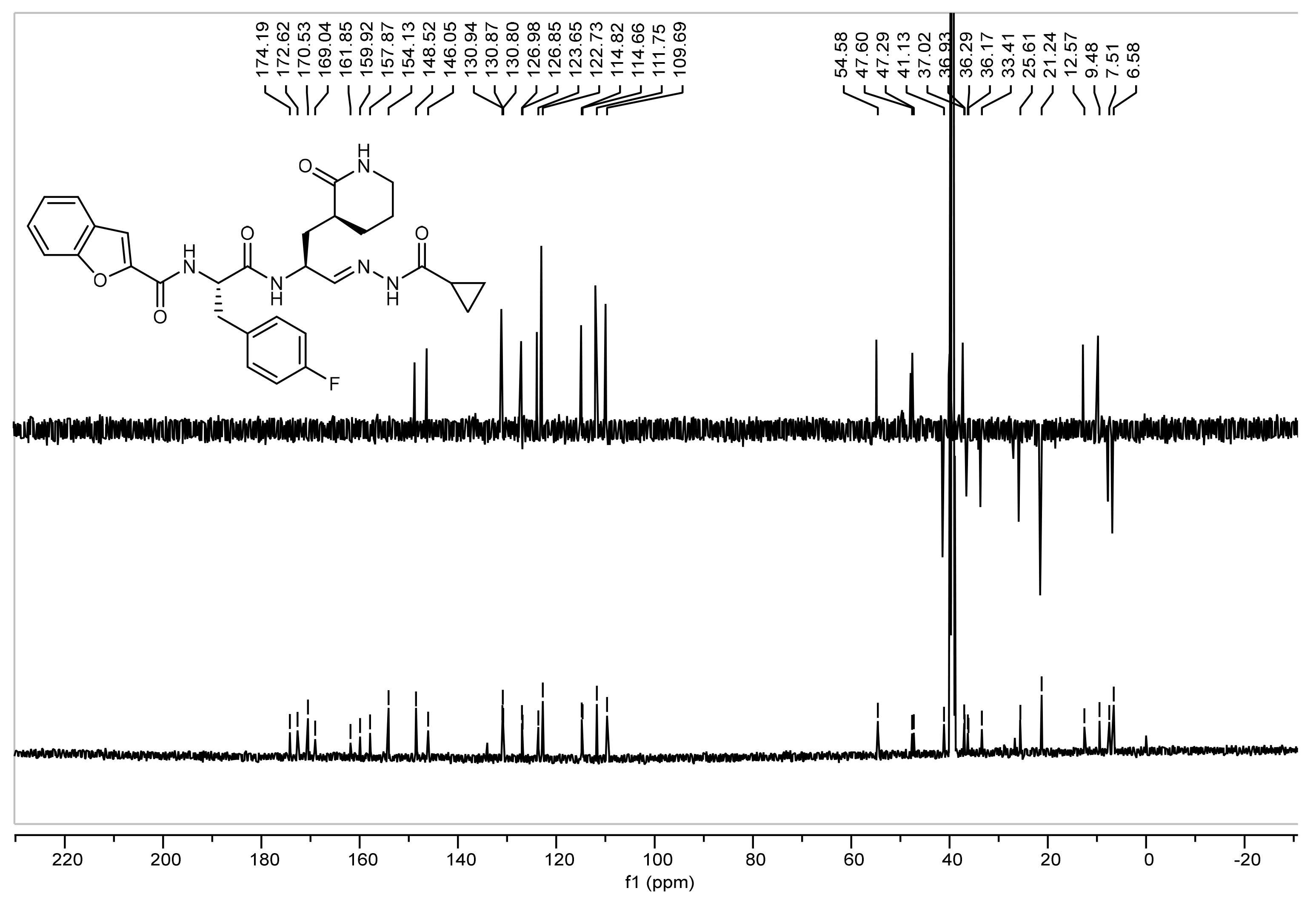


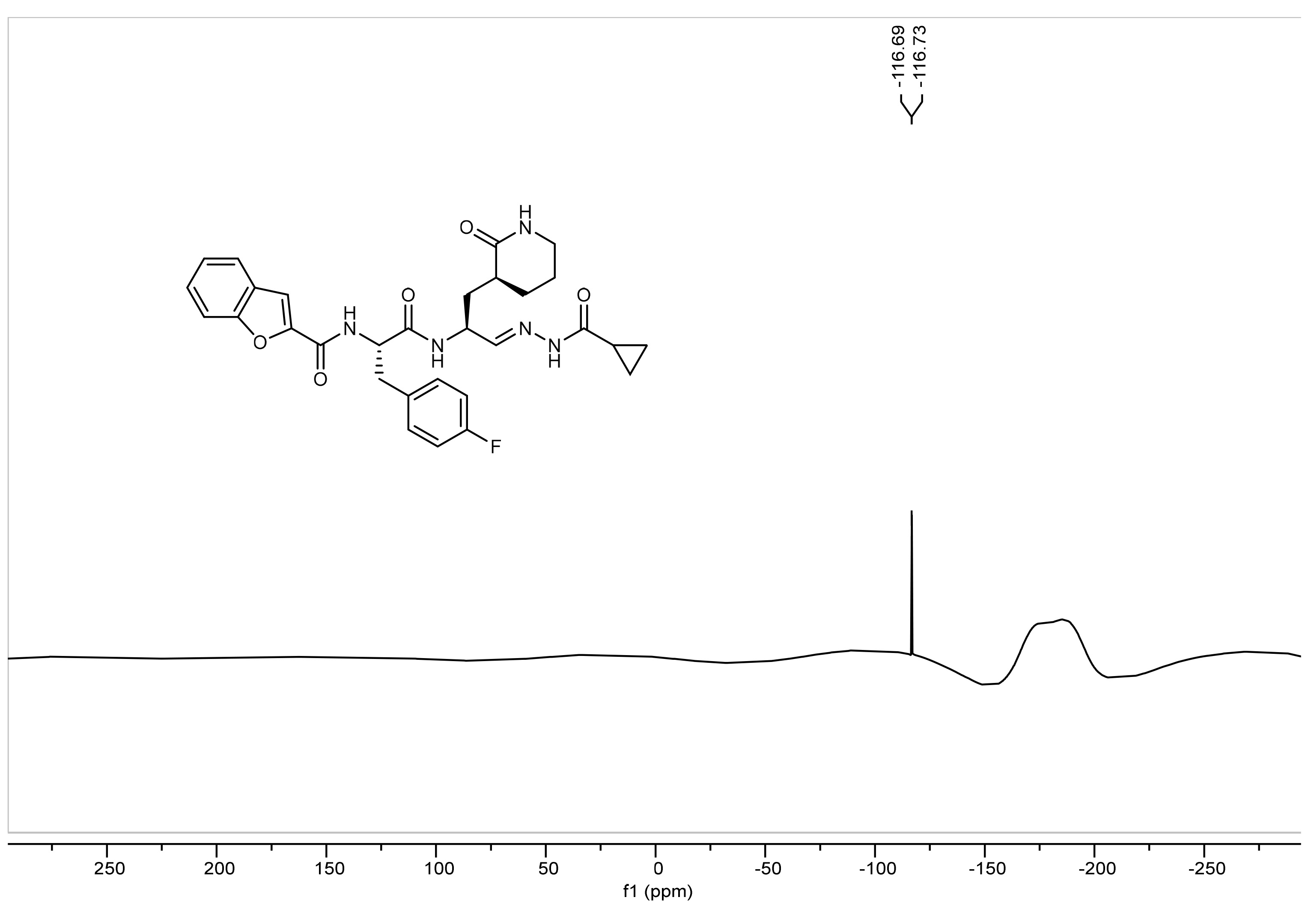


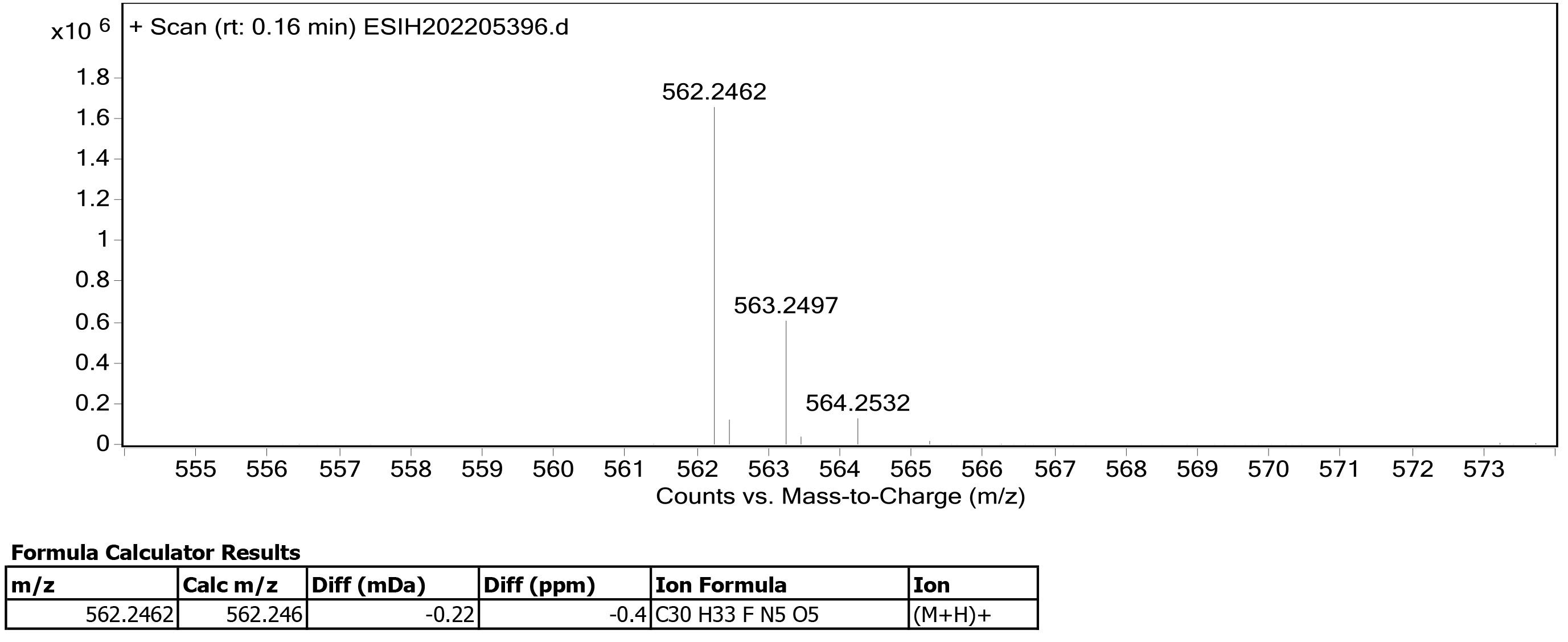


**^1^H, ^13^C and ^19^F NMR, HRMS spectra of 14h.**

**
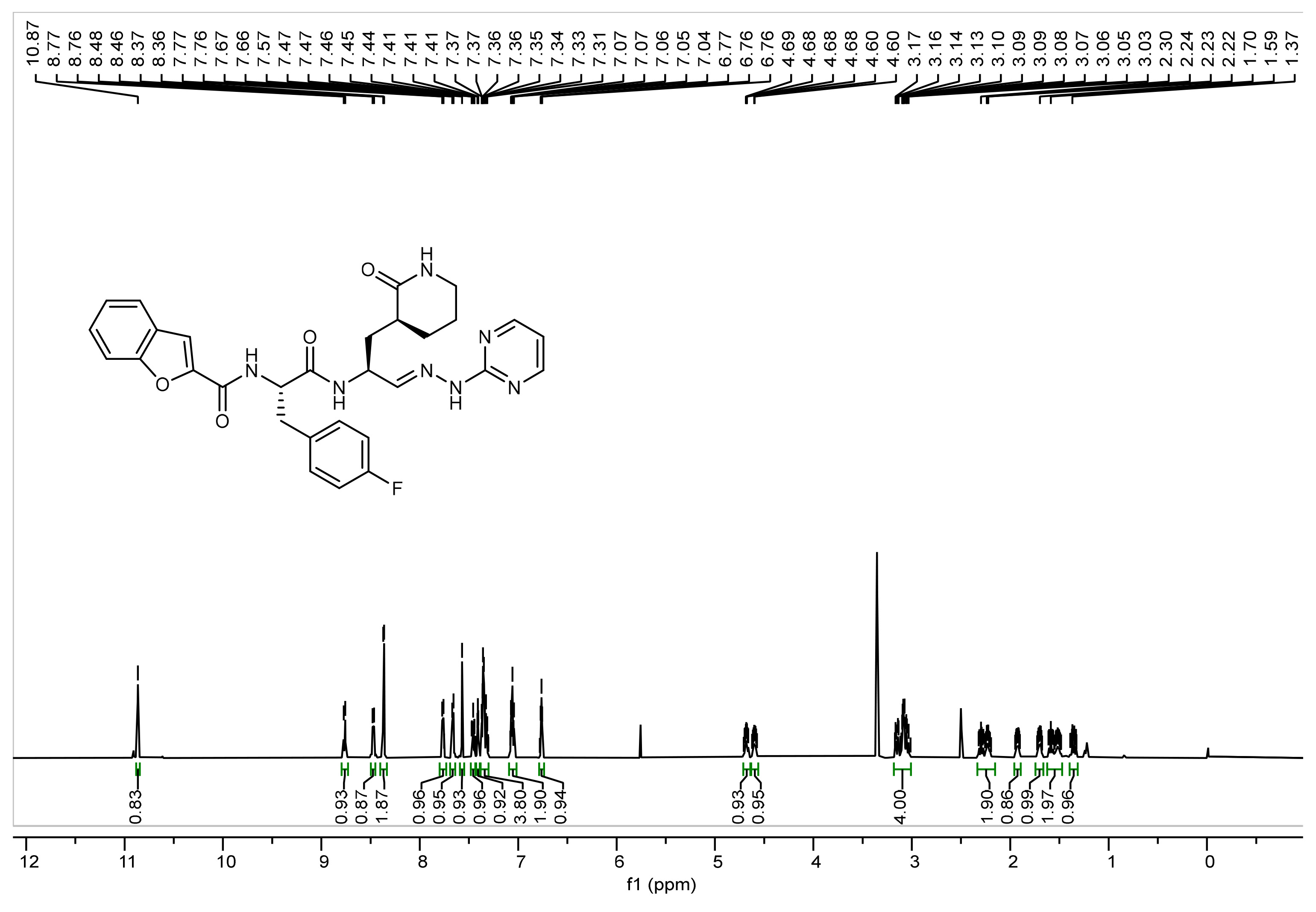
**

**
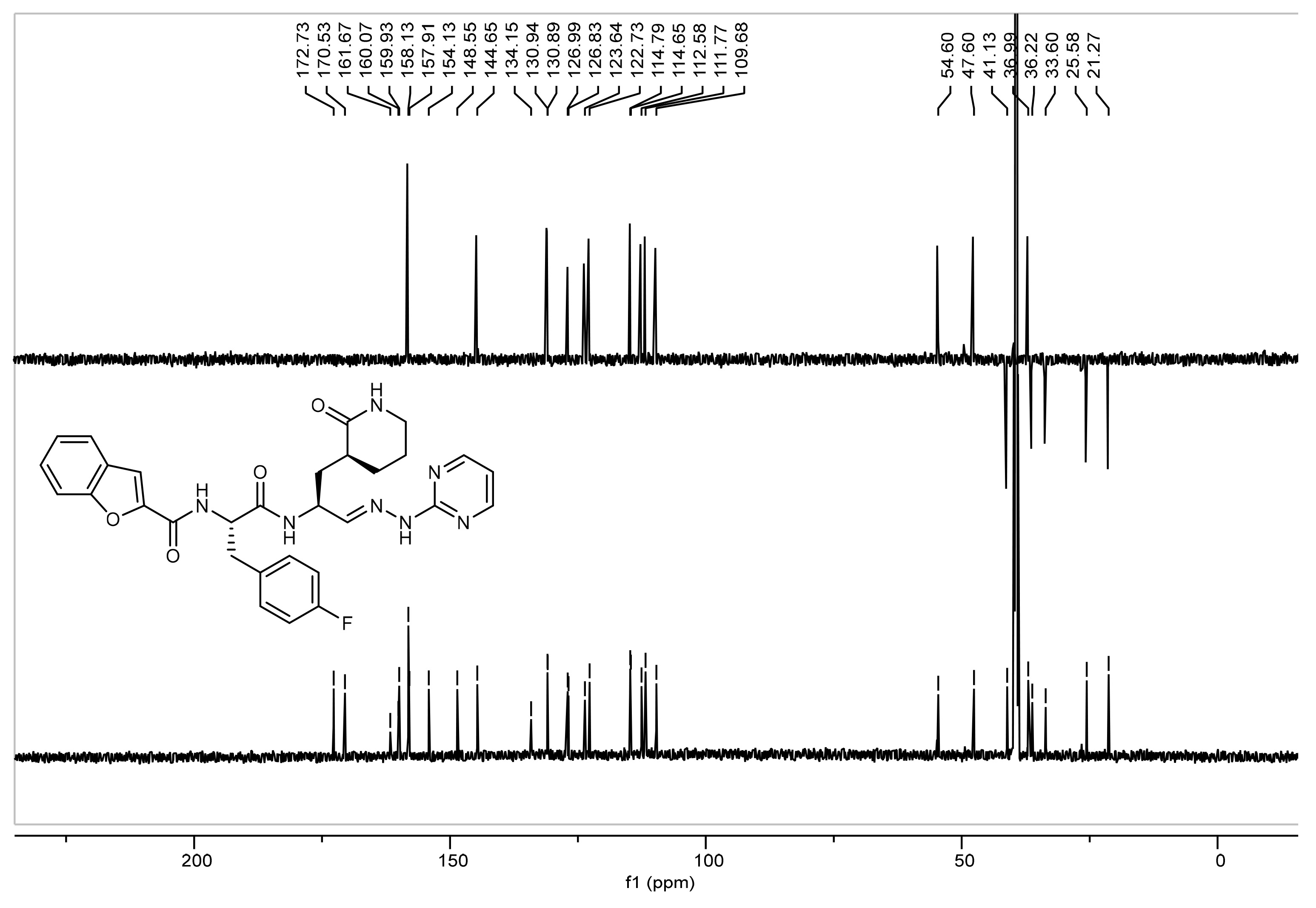
**

**
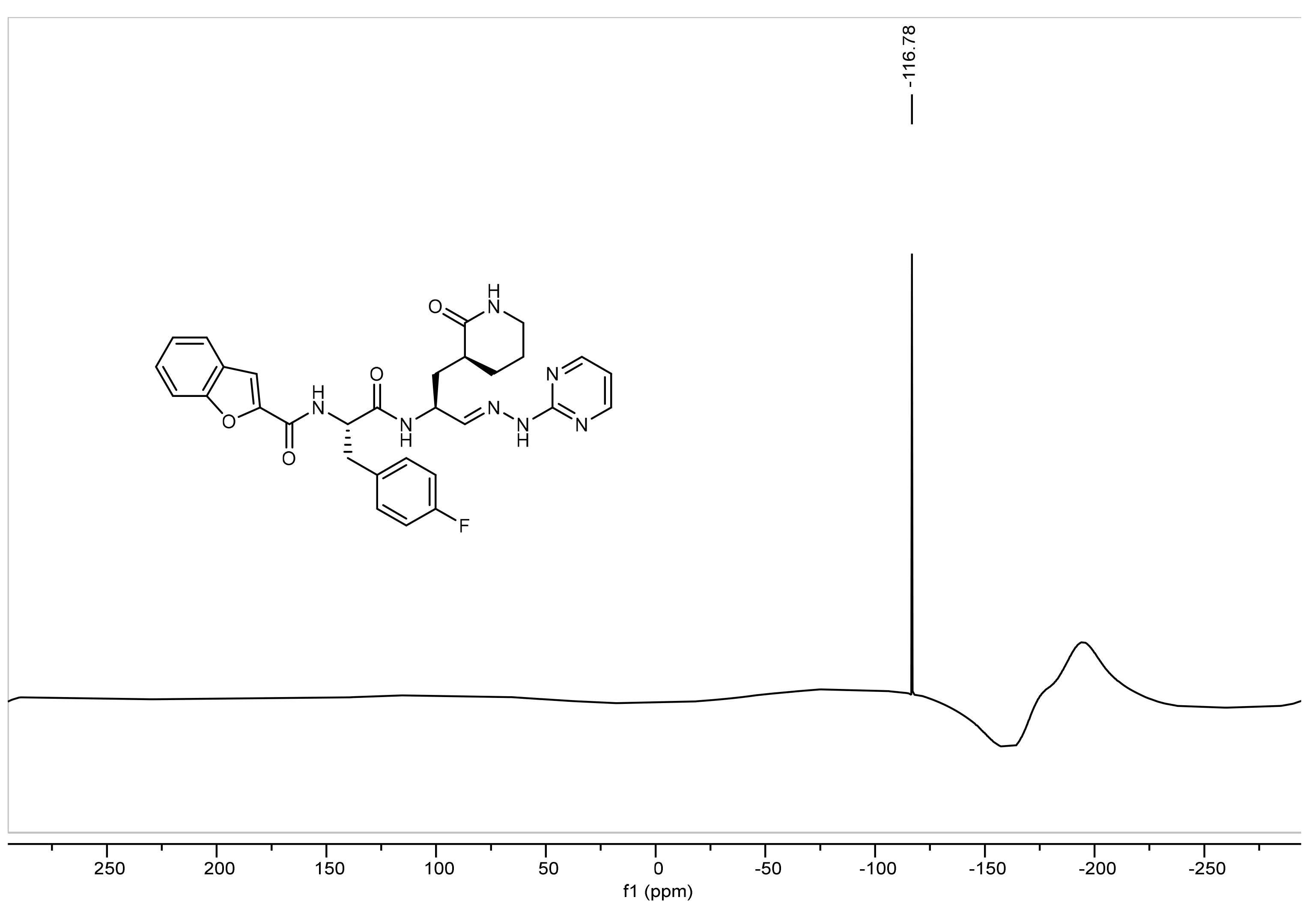
**

**
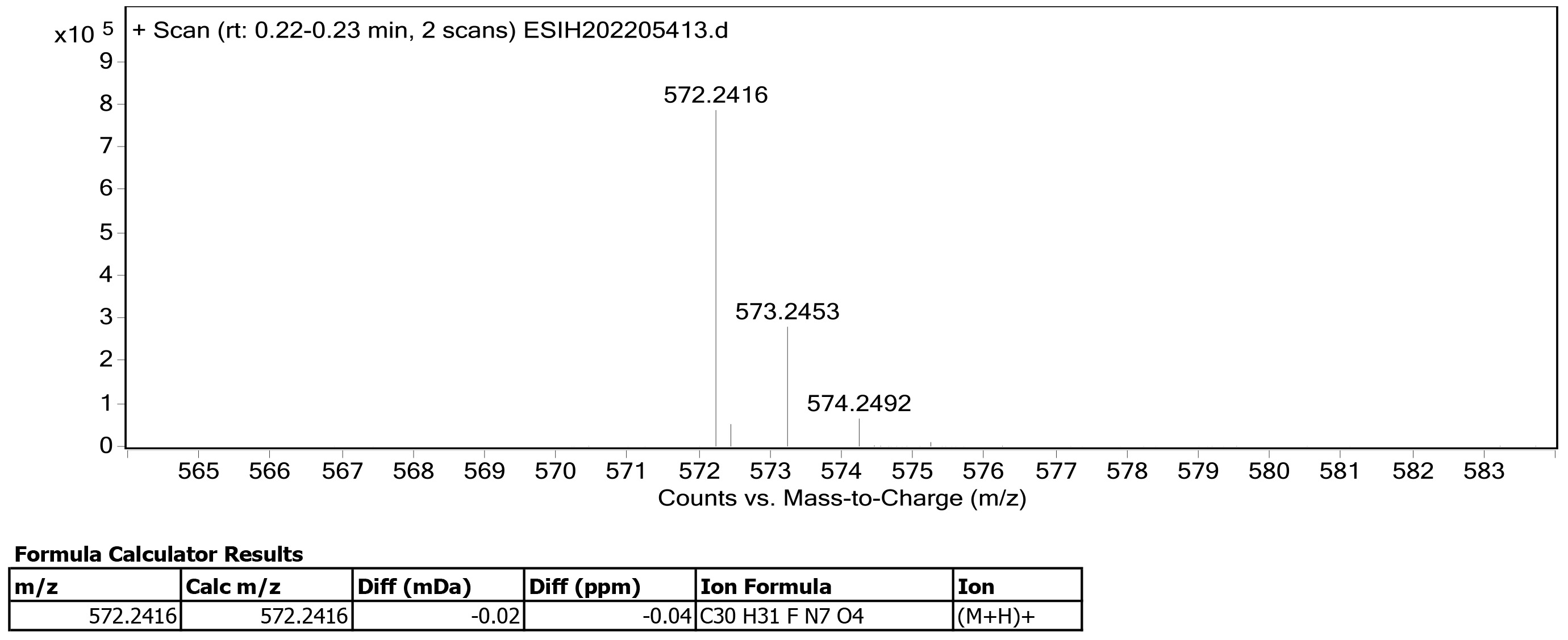
**

**^1^H, ^13^C and ^19^F NMR, HRMS spectra of 14i.**


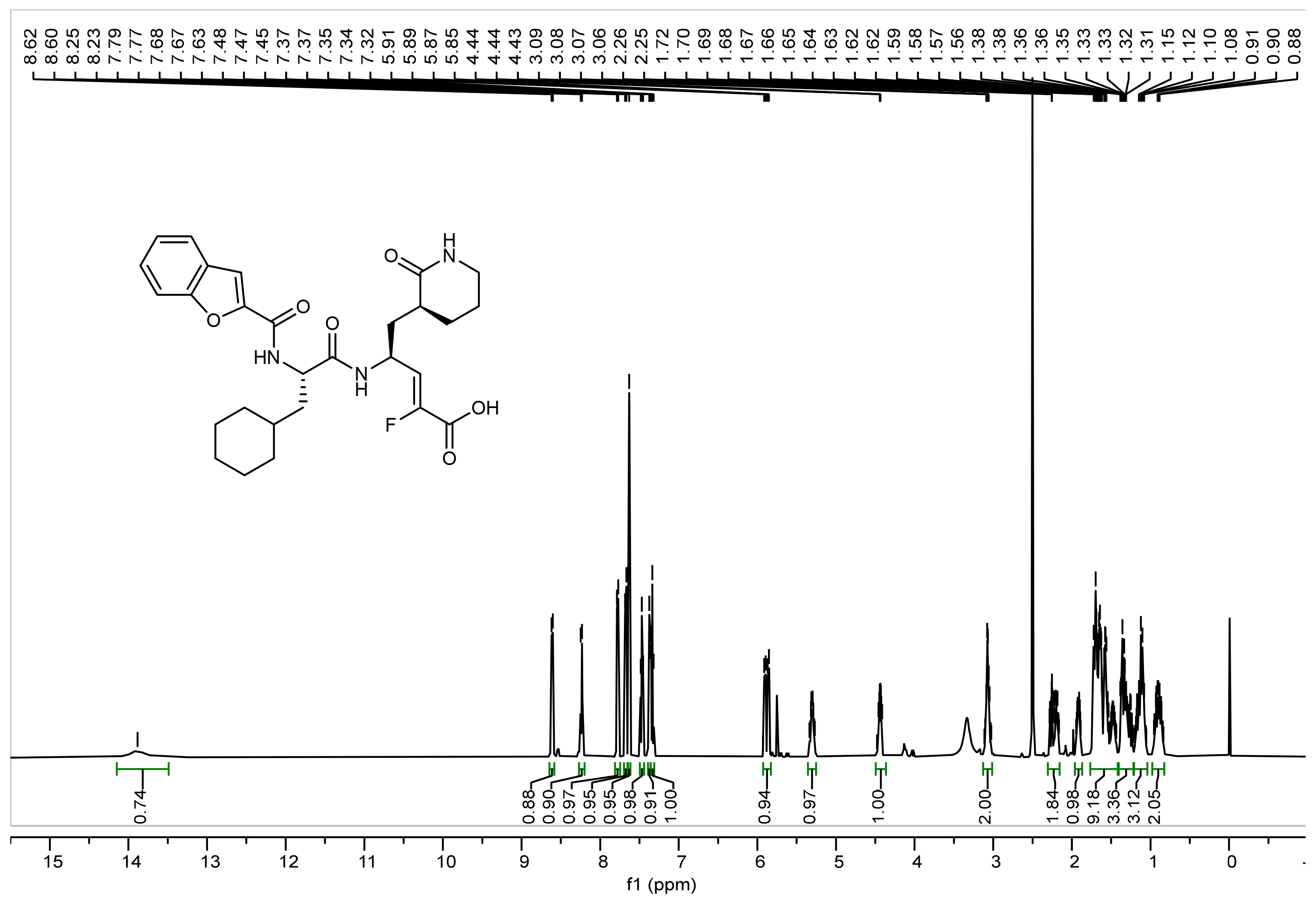


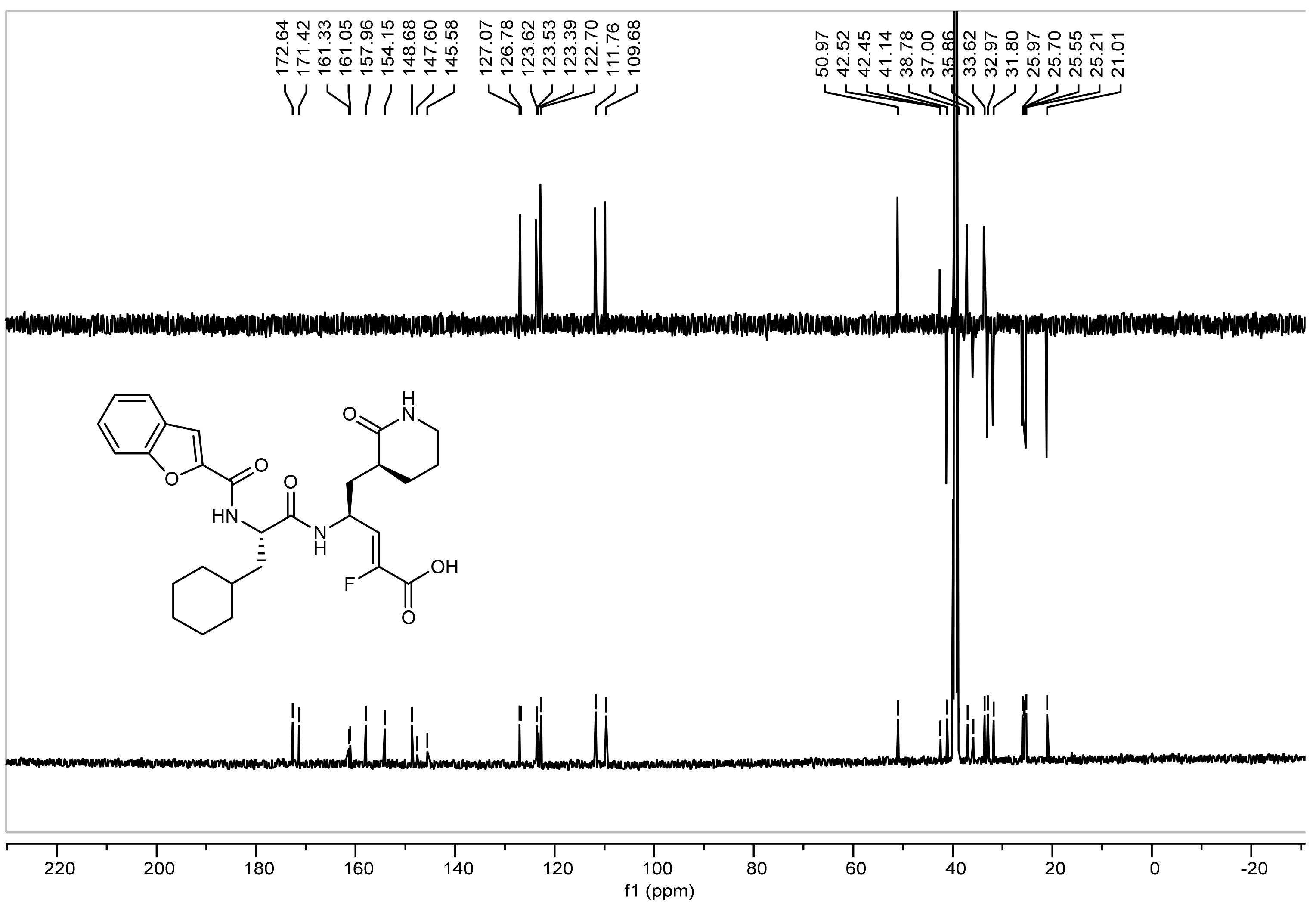


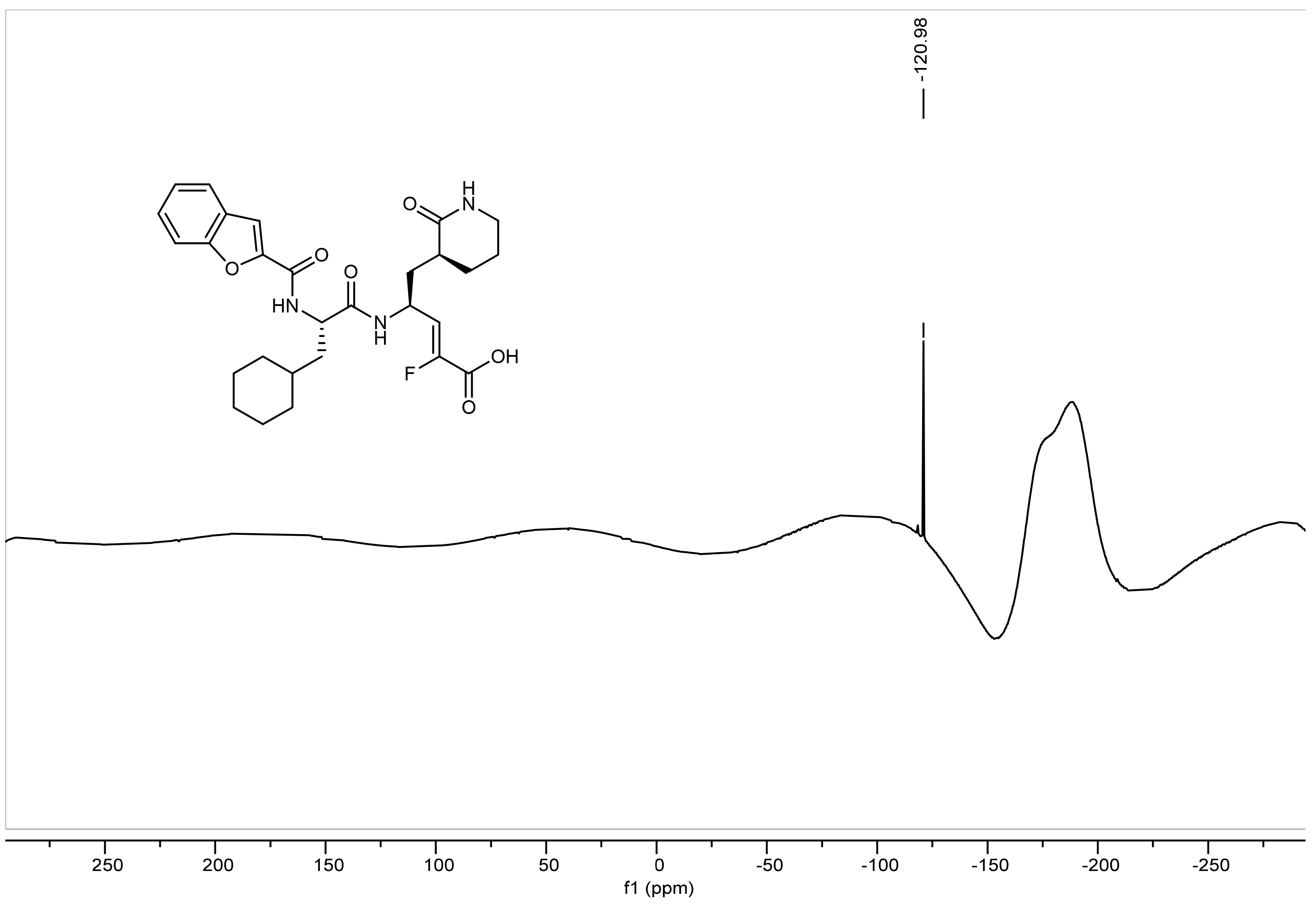


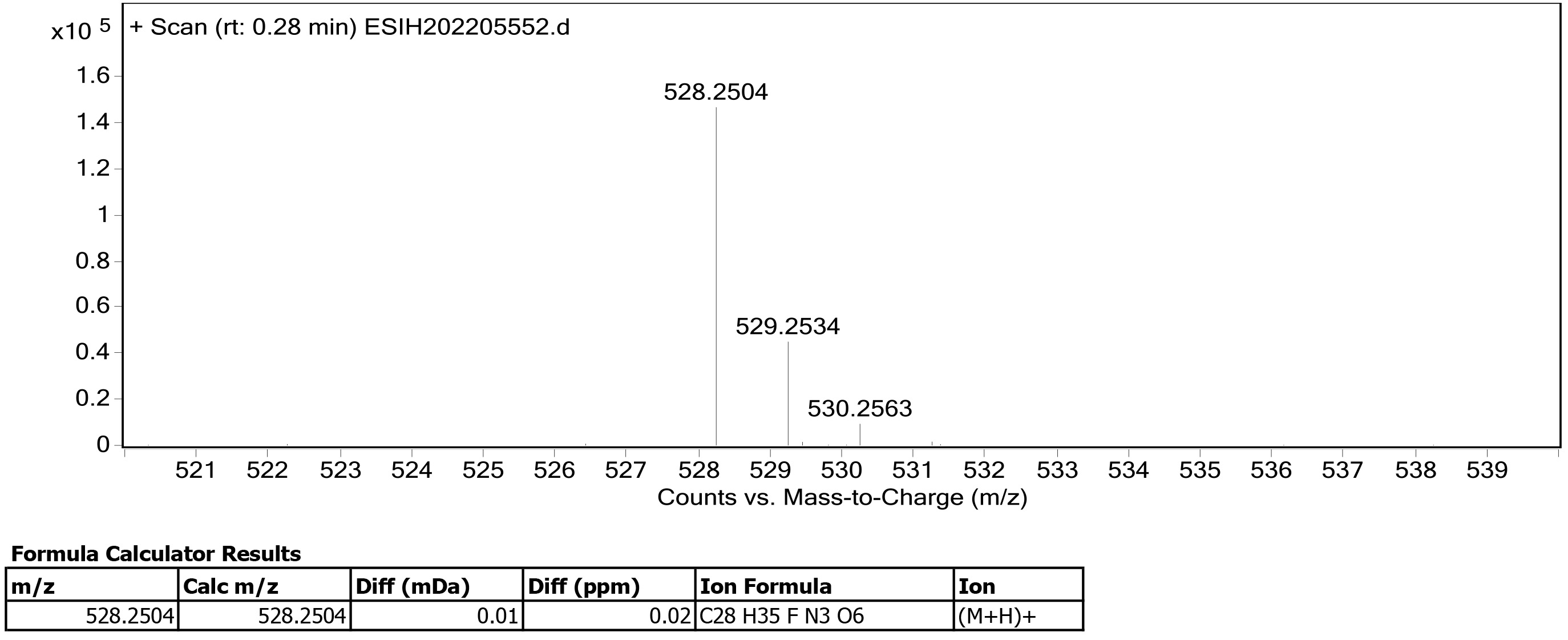


**^1^H, ^13^C and ^19^F NMR, HRMS spectra of 14j.**


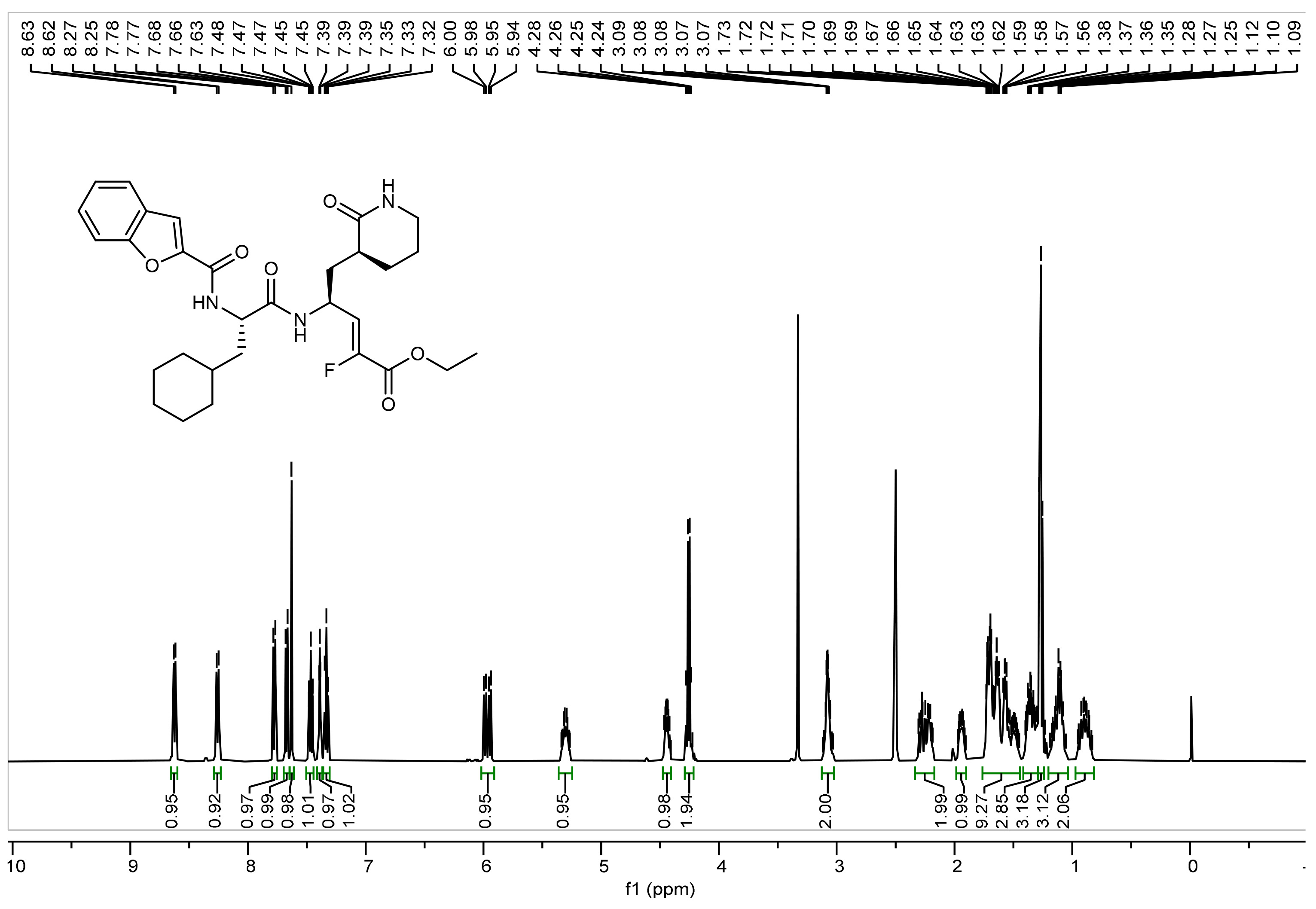


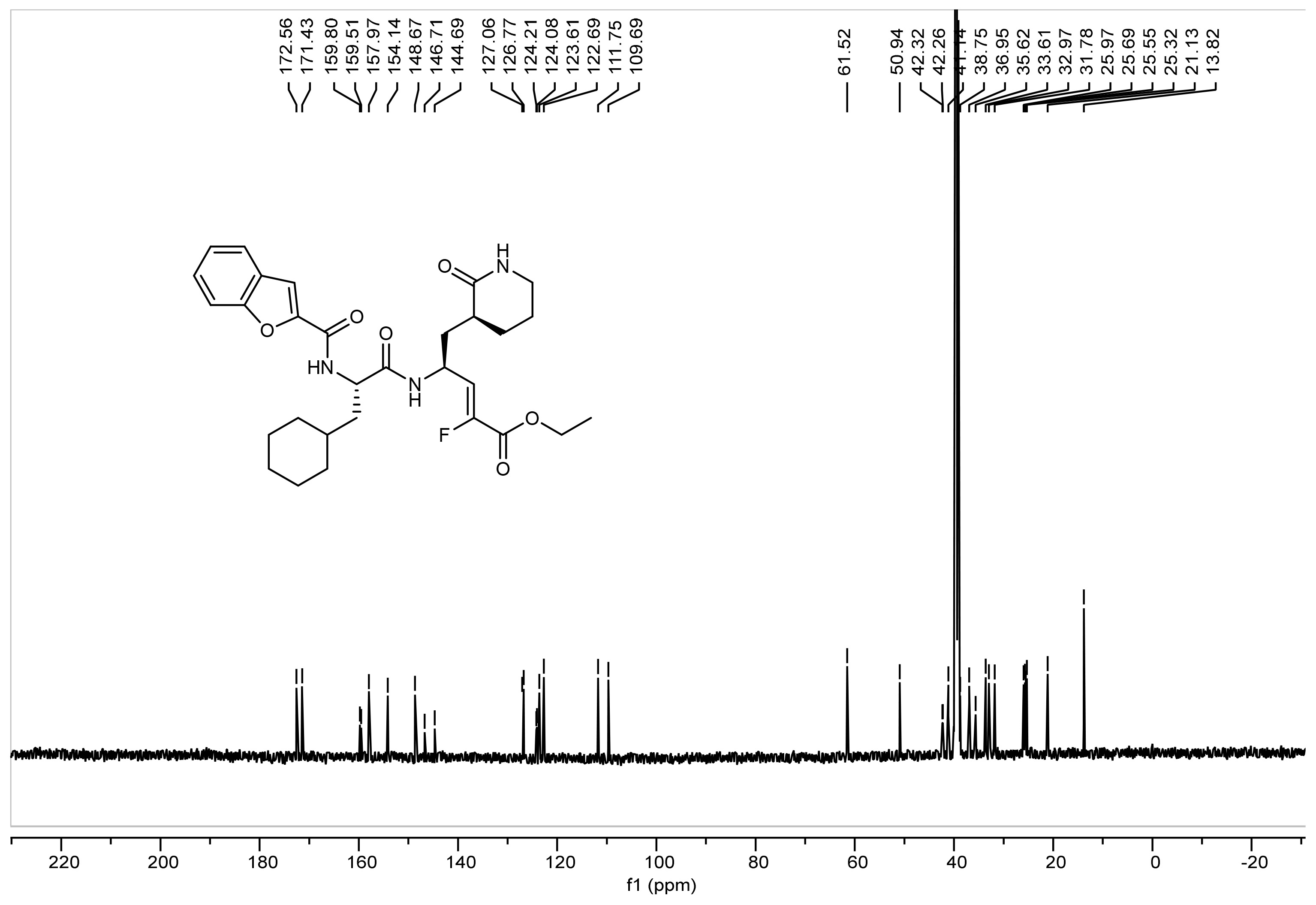


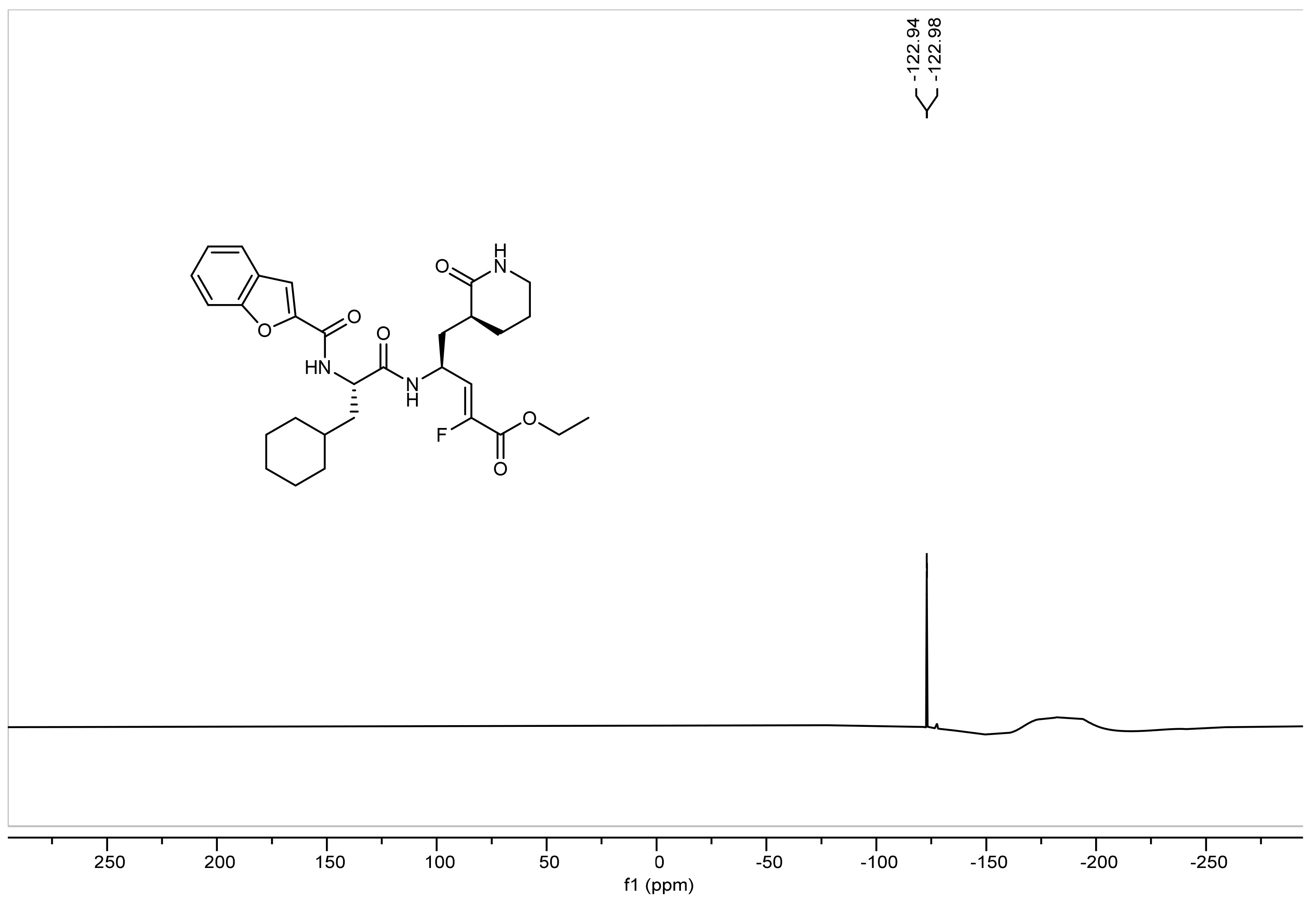


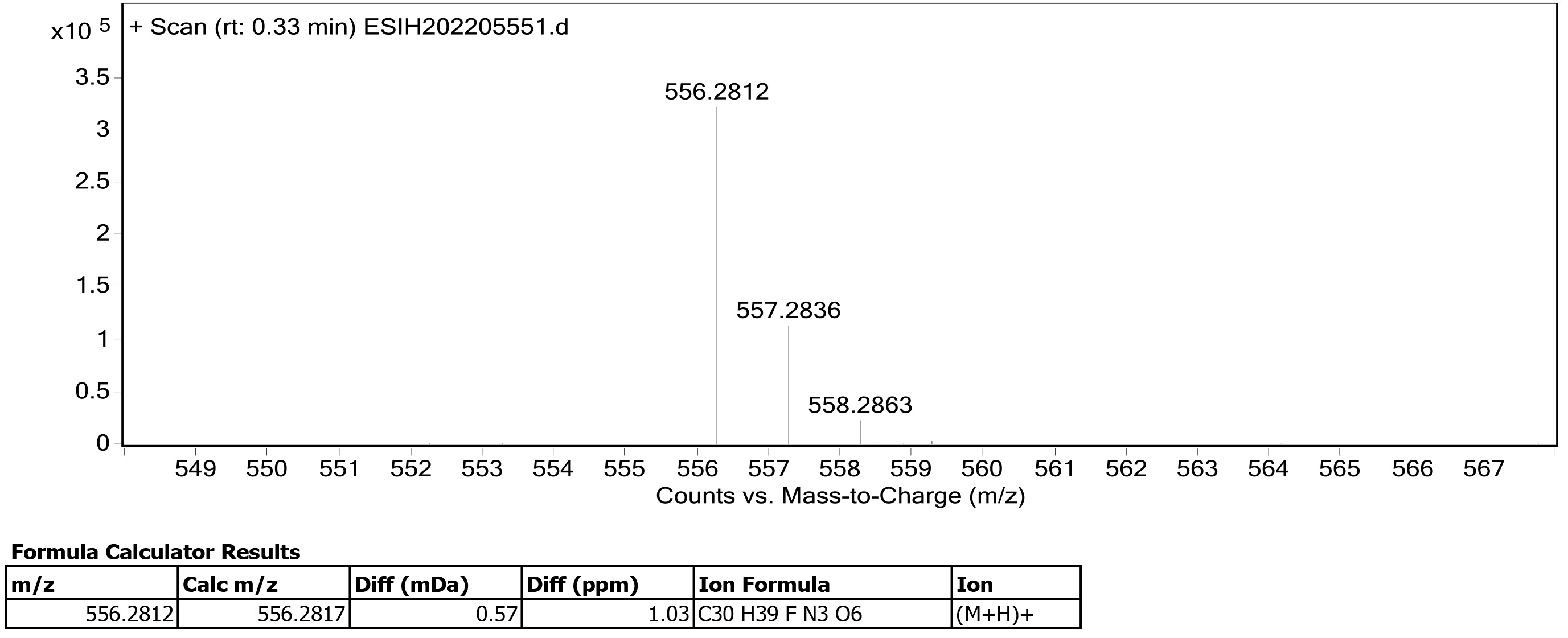


**^1^H, ^13^C and ^19^F NMR, HRMS spectra of 14k.**


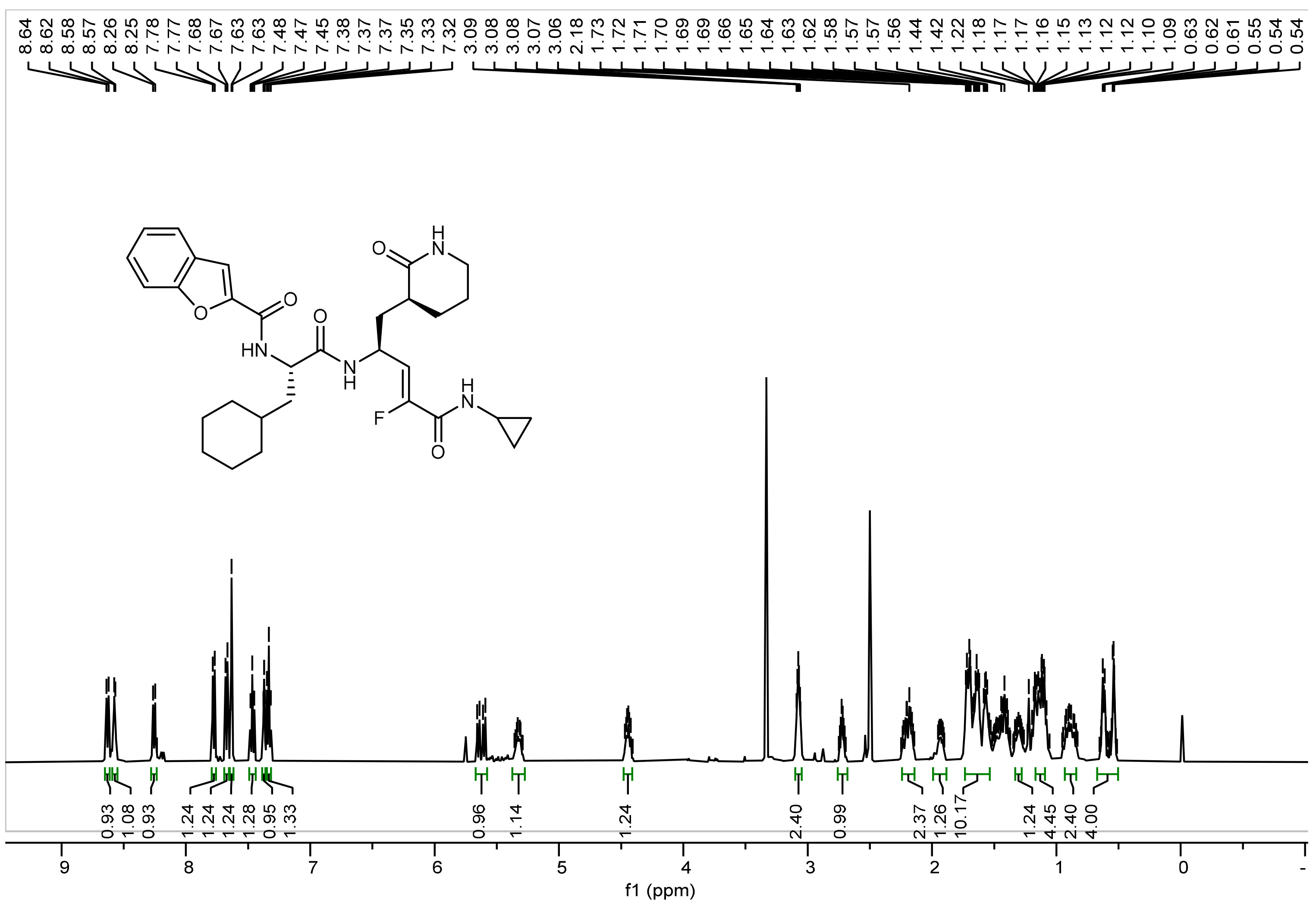


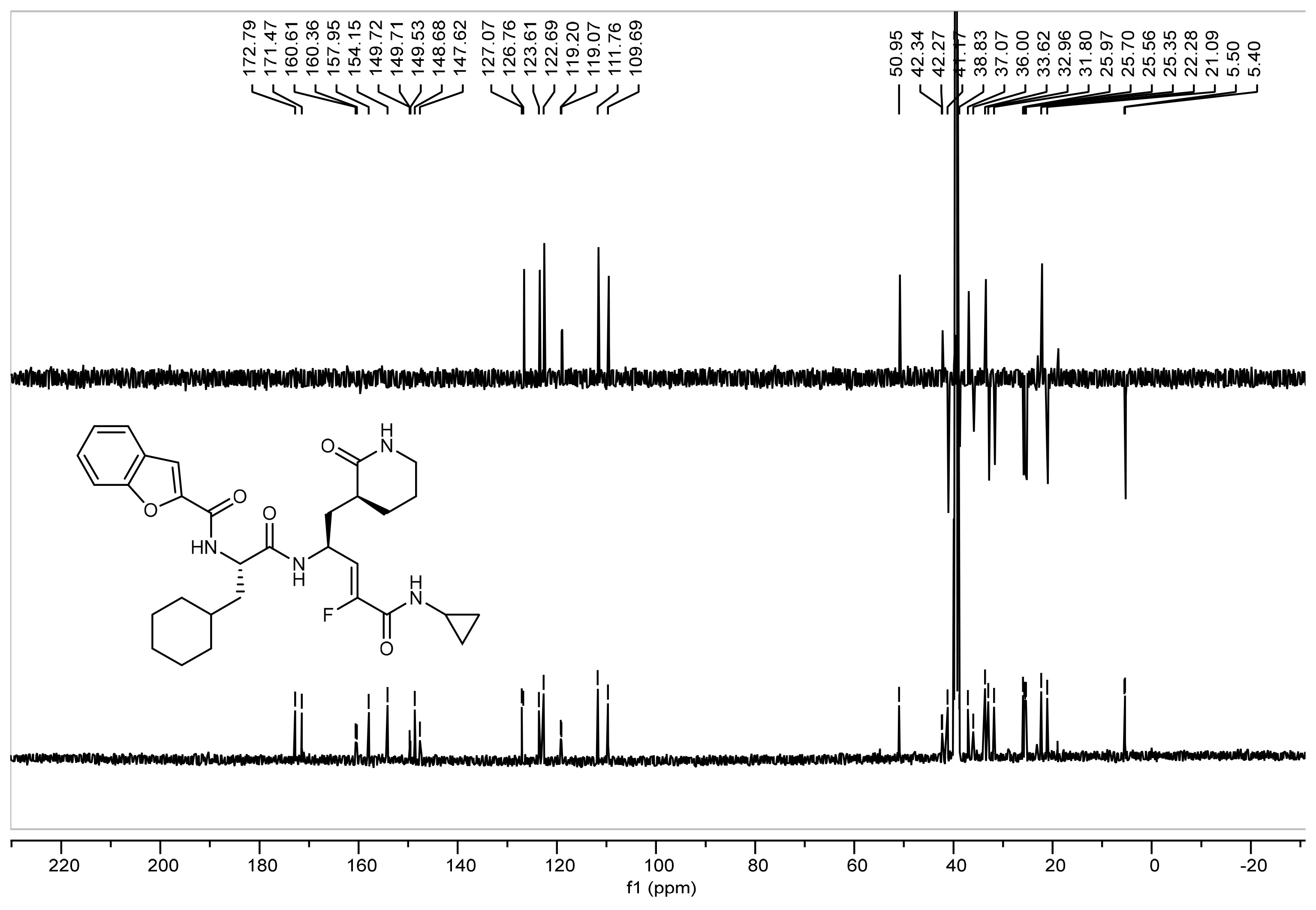


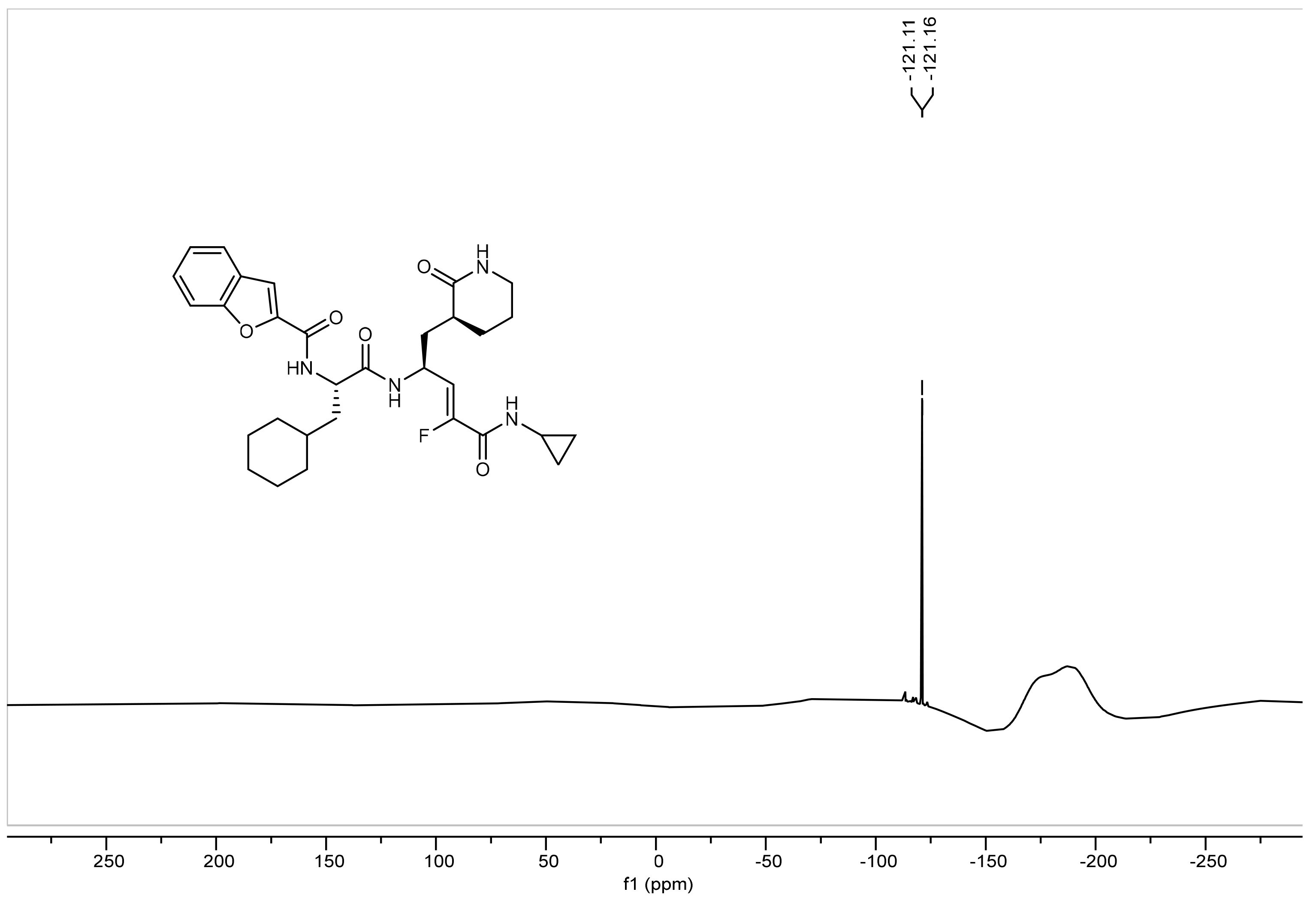


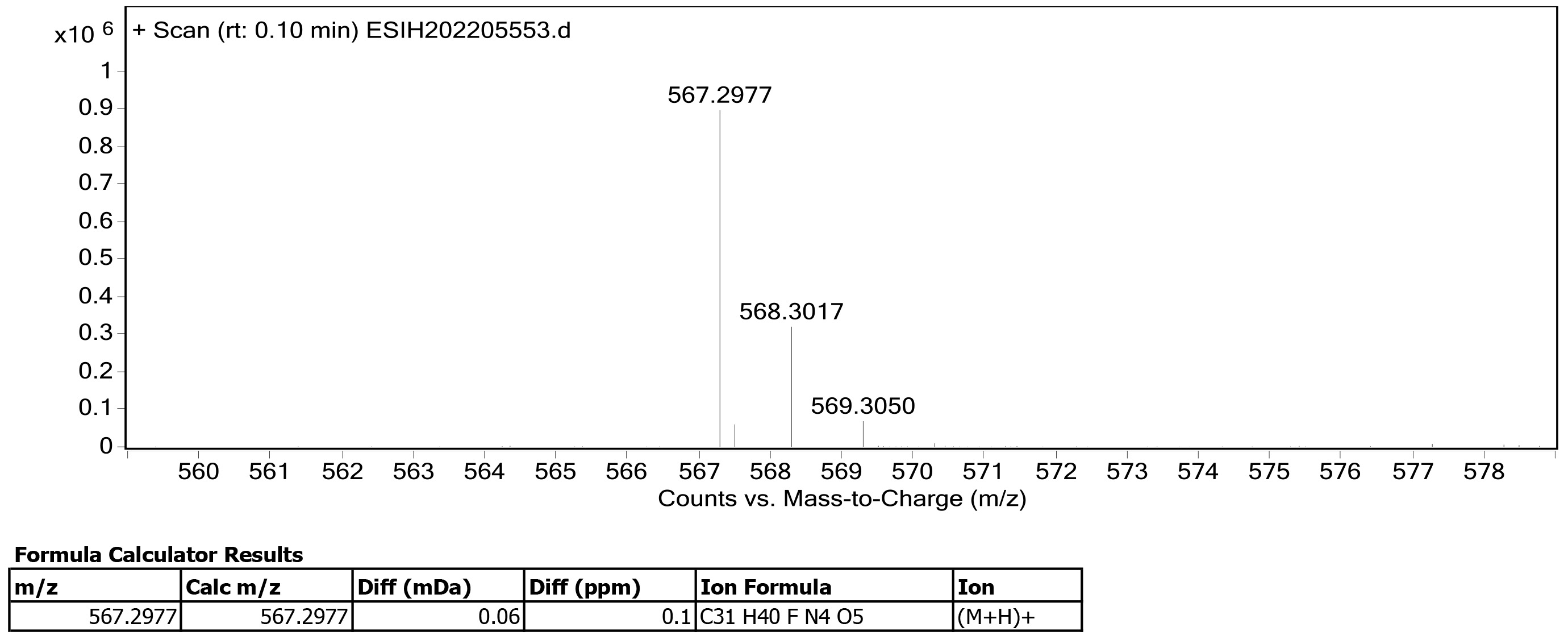


**^1^H and ^13^C NMR, HRMS spectra of 14l.**


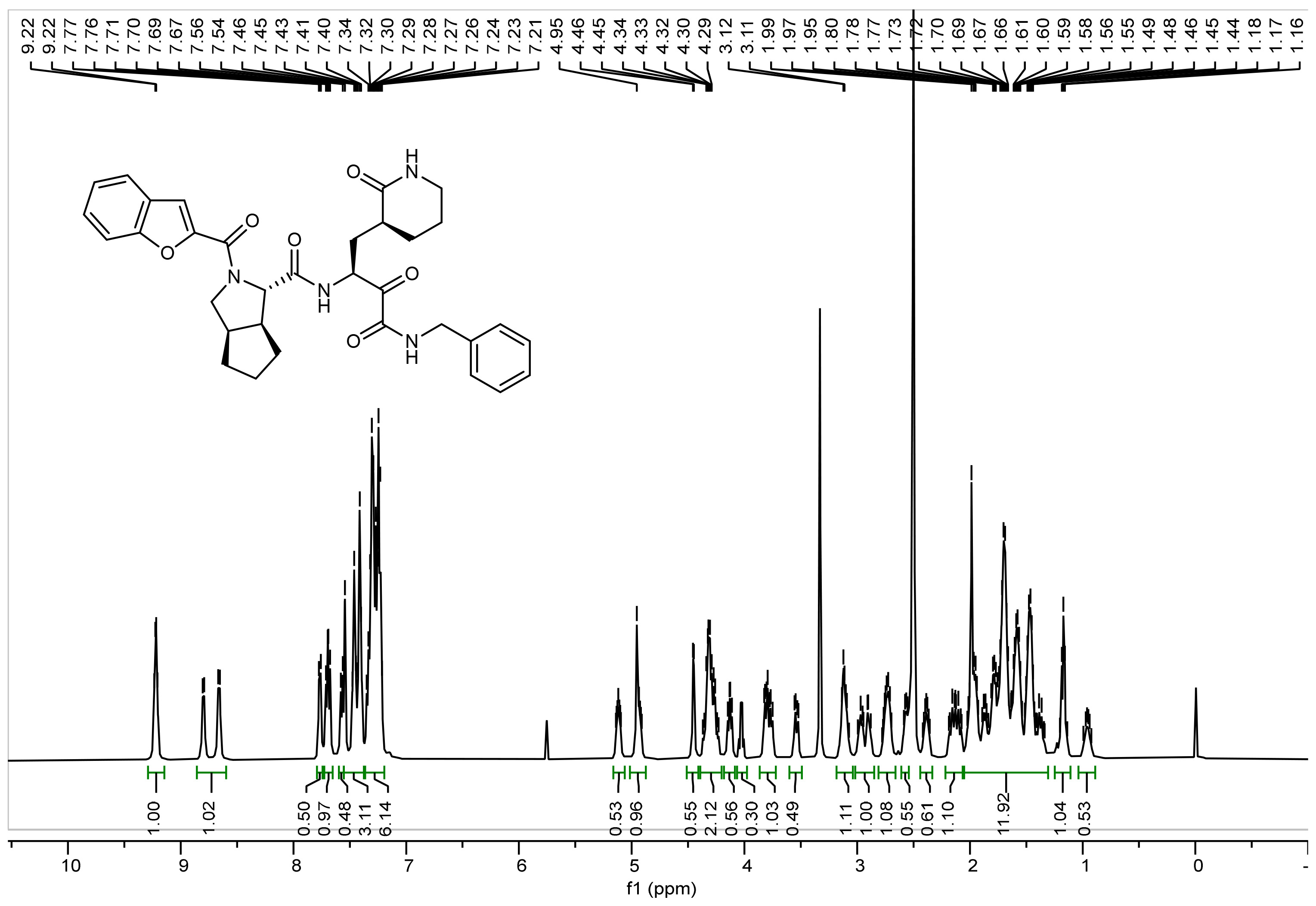


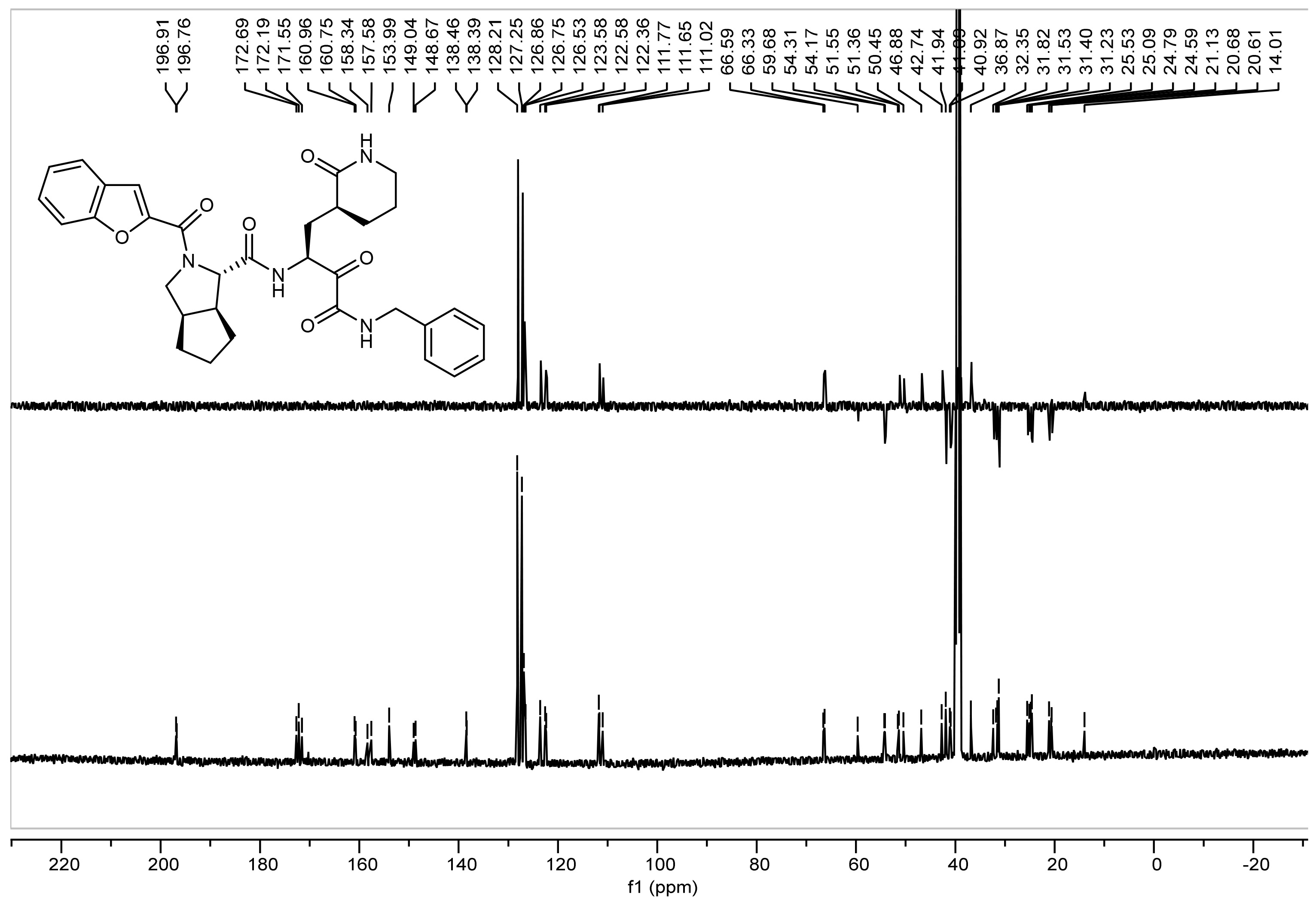


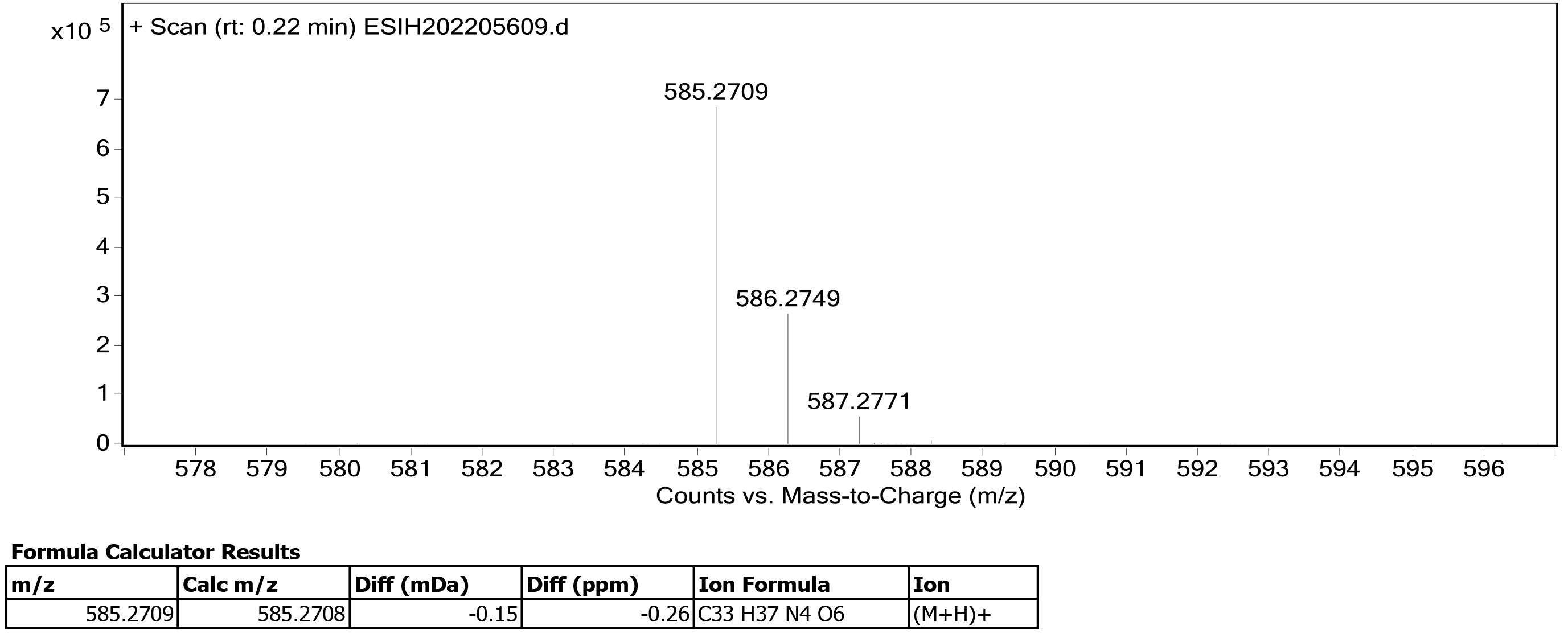


**^1^H and ^13^C NMR, HRMS spectra of 14m.**


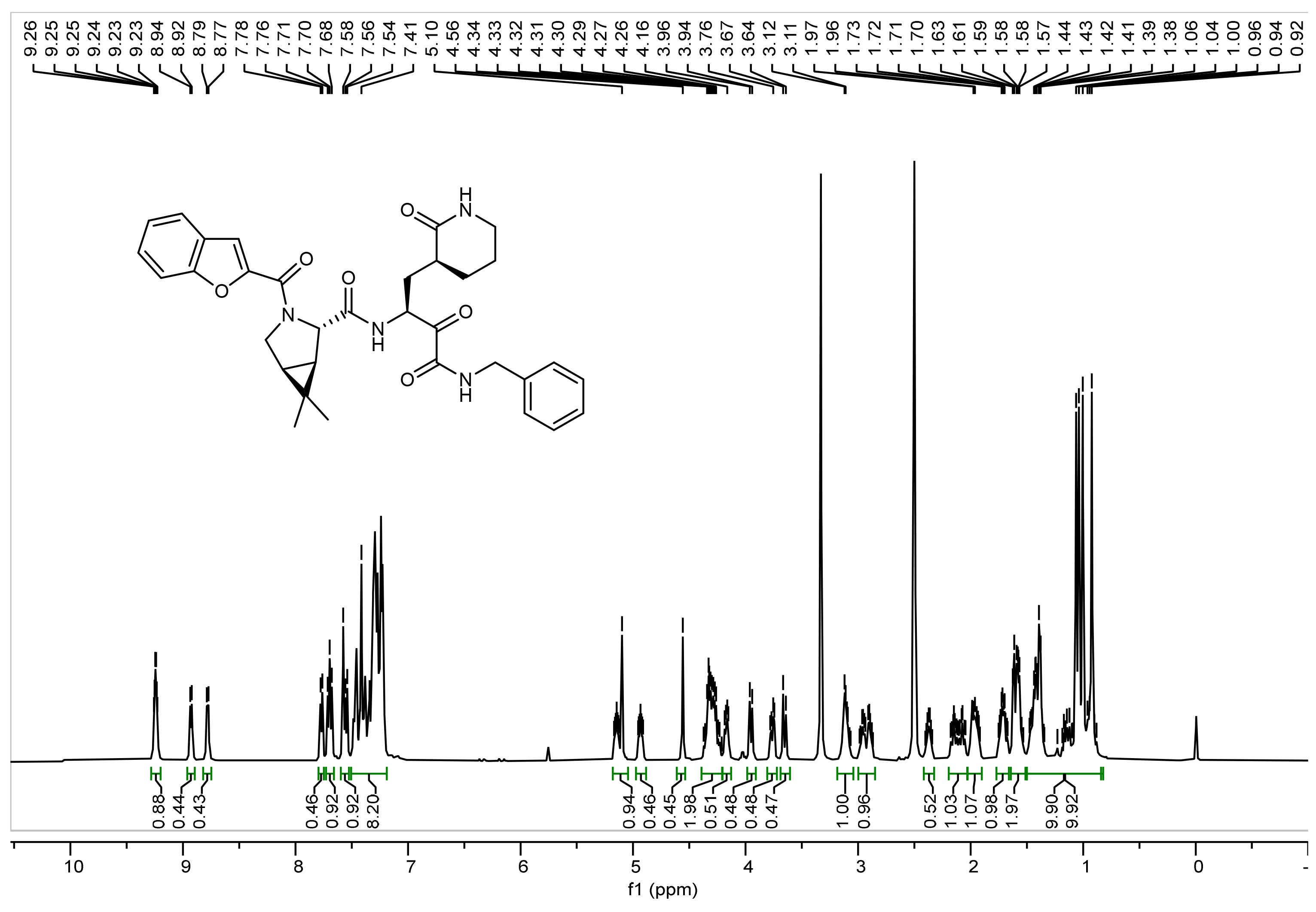


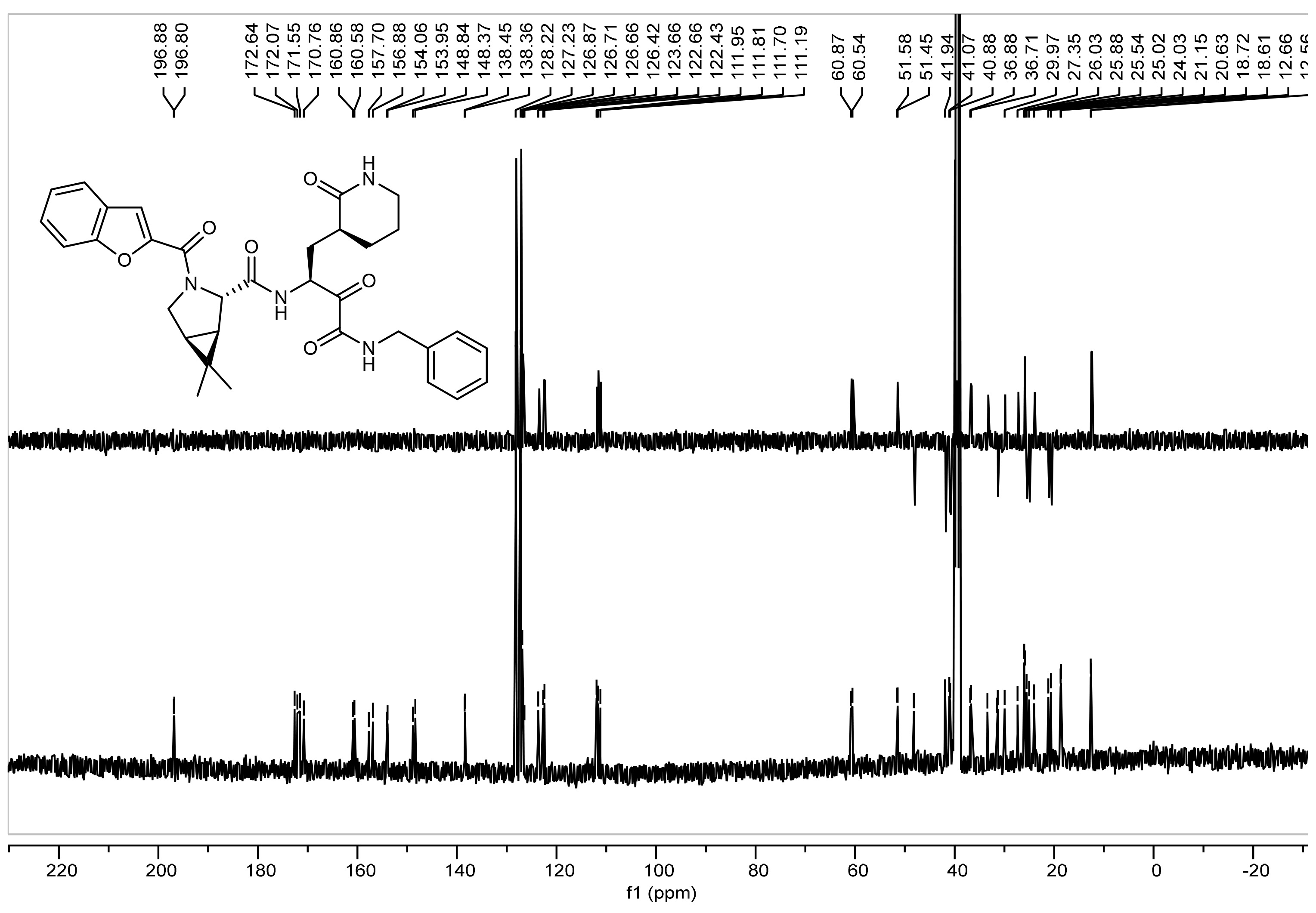


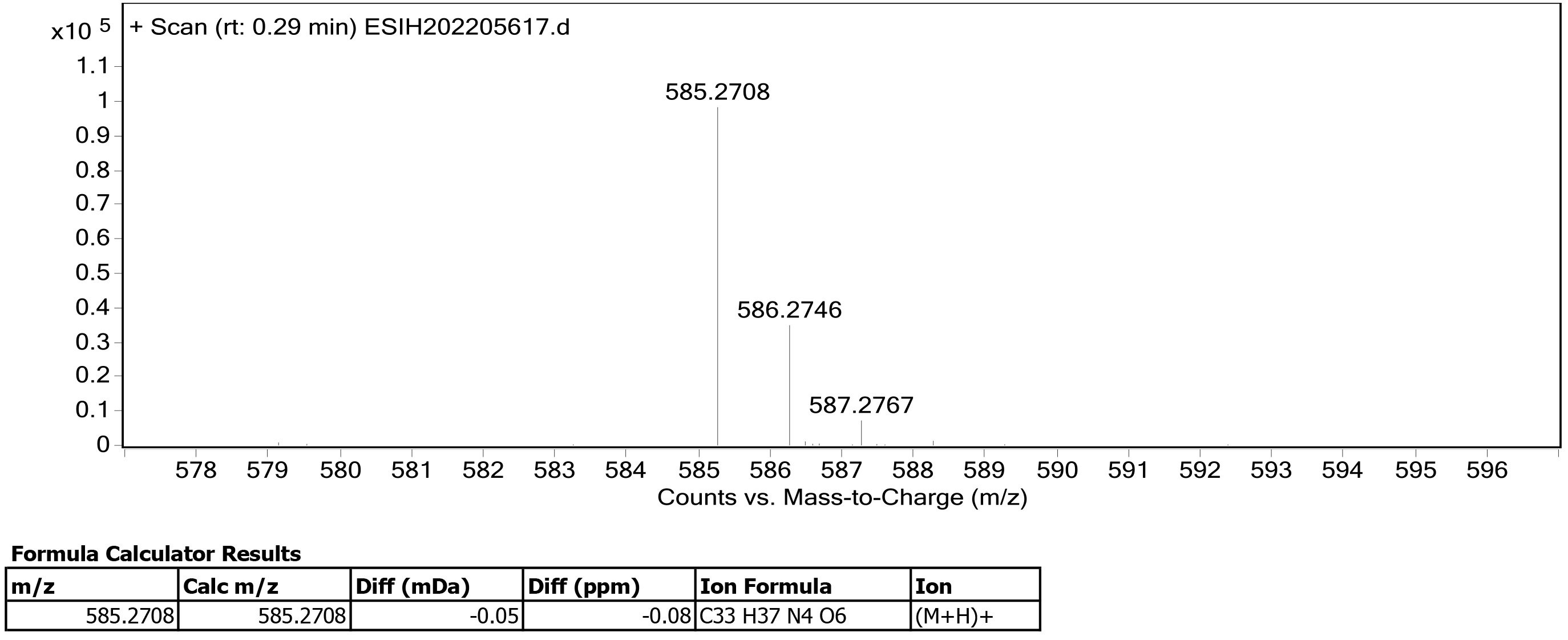


**Table S1 Preliminary structure-activity relationship exploration of synthesized peptidomimetic covalent inhibitors based on their inhibitory activity against human CTSL.**

| **Compound** | **linker** | **warhead** | **IC_50_ (nM)^a^** |
| --- | --- | --- | --- |
| **14a** |  |  | 3.34 ± 0.52 |
| **14b** |  |  | 6.88 ± 0.81 |
| **14c** |  |  | 50.32 ± 12.12 |
| **14d** |  |  | 77.21 ± 12.00 |
| **14e** |  |  | 3,937 ± 3,046 |
| **14f** |  |  | 8,526 ± 8,339 |
| **14g** |  |  | 1,323 ± 461 |
| **14h** |  |  | 1,599 ± 570 |
| **14i** |  |  | 2,162 ± 503 |
| **14j** |  |  | 30%^b^ |
| **14k** |  |  | 37%^b^ |
| **14l** |  |  | 12%^b^ |
| **14m** |  |  | 1,875 ± 899 |

^a^ Each sample was tested in triplicate unless otherwise indicated. Data are shown as mean ± SD. ^b^ Inhibition rate at a concentration of 1 μM. Each sample was tested only once and the data are shown as mean.

**Table S2 Inhibitory activity of 14a and 14b against common human and viral proteases.**

| **Target** | **IC_50_^a^ or inhibition rate^b^** | |
| --- | --- | --- |
|  | **14a** | **14b** |
| Human TMPRSS2 | -1 ± 1%^c^ | 3 ± 1%^c^ |
| Human Furin | 3 ± 4% | -4 ± 1% |
| Human CTSB | 1.23 ± 0.25 nM | 1.01 ± 0.04 nM |
| Human CTSC | 181.1 ± 30.4 nM | 66.22 ± 7.85 nM |
| Human CTSD | 21 ± 1% | 29 ± 2% |
| Human CTSE | 13 ± 1% | 11 ± 2% |
| Human CTSG | 20 ± 3% | 4 ± 4% |
| Human CTSX | 722.0 ± 108.2 nM | 1,306 ± 467 nM |
| Human Caspase 3 | 25 ± 2% | 39 ± 2% |
| Human Thrombin | 9 ± 3% | 4 ± 1% |
| Human Elastase | 5 ± 1% | 2 ± 2% |
| Human Chymotrypsin C | -2 ± 1% | 8 ± 4% |
| Human Trypsin | 3 ± 1% | 1 ± 1% |
| SARS-CoV-2 M^pro^ | 12.57 ± 0.56 μM | 0.99 ± 0.11 μM |
| SARS-CoV-2 RdRp | 8.02 ± 0.04 μM | 3.21 ± 0.25 μM |
| SARS-CoV-2 PL^pro^ | 3 ± 2% | 8 ± 1% |

^a^ Each sample was tested in triplicate unless otherwise indicated. Data are shown as mean ± SD. ^b^ Inhibition rate at a concentration of 20 μM unless otherwise indicated. ^c^ Inhibition rate at a concentration of 10 μM.

**Table S3 Data collection and refinement statistics.**

|  | CTSL-**14a** | CTSL-**14b** | CAPN1-**14a** |
| --- | --- | --- | --- |
|  | PDB code: 7W33 | PDB code: 7W34 | PDB code: 7W7O |
| **Data Collection** |  |  |  |
| Space group | *I422* | *I422* | *P2_1_2_1_2_1_* |
| Wavelength (Å) | 0.9792 | 0.9792 | 0.9785 |
| Cell dimensions |  |  |  |
| *a, b, c* (Å) | 109.20, 109.20, 92.43 | 109.36, 109.36, 92.48 | 50.13, 63.98, 99.75 |
| α, β, γ (°) | 90, 90, 90 | 90, 90, 90 | 90, 90, 90 |
| Resolution (Å) | 34.53-2.39 (2.48-2.39) | 39.69-2.89 (3.00-2.89) | 27.71-1.59 (1.65-1.59) |
| No. of unique reflections | 11297 (1079) | 6524 (630) | 42400 (3849) |
| Completeness (%) | 99.33 (96.33) | 99.68 (98.58) | 96.75 (89.45) |
| *R*_merge_ | 0.3241 (4.072) | 0.5636 (4.567) | 0.07555 (1.188) |
| Mean *I*/σ*I* | 11.19 (1.63) | 10.52 (1.34) | 19.43 (2.03) |
| *CC*1/2 | 99.7 (77.1) | 99.3 (66.4) | 99.9 (74.5) |
| Redundancy | 25.6 (26.1) | 26.0 (25.2) | 13.1 (12.6) |
| Wilson B factors (Å^2^) | 49.14 | 61.79 | 22.29 |
|  |  |  |  |
| **Refinement** |  |  |  |
| Resolution (Å) | 34.53-2.39 | 39.69-2.89 | 27.71-1.59 |
| No. of reflections used | 11271 (1076) | 6513 (626) | 42373 (3849) |
| *R*_work_ / *R*_free_ | 23.19/25.73 | 23.67/29.40 | 16.61/19.63 |
| No. atoms |  |  |  |
| Protein | 1697 | 1697 | 2658 |
| Ligand/ion | 45 | 44 | 48 |
| Water | 5 | 0 | 315 |
| *B*-factors (Å^2^) |  |  |  |
| Protein | 68.58 | 60.95 | 29.16 |
| Ligand/ion | 67.47 | 60.65 | 30.52 |
| Water | 67.24 |  | 41.38 |
| R.m.s. deviations |  |  |  |
| Bond lengths (Å) | 0.012 | 0.004 | 0.016 |
| Bond angles (°) | 1.38 | 0.95 | 1.43 |
| Ramachandran plot (%) |  |  |  |
| Favored (%) | 97.71 | 95.87 | 98.15 |
| Allowed (%) | 2.29 | 3.67 | 1.54 |
| Outliers (%) | 0 | 0.46 | 0.31 |

(Continued)

|  | CAPN1-**14b** | M^pro^-**14a** | M^pro^-**14b** |
| --- | --- | --- | --- |
|  | PDB code: 7X79 | PDB code: 8GXG | PDB code: 8GXH |
| **Data Collection** |  |  |  |
| Space group | *P2_1_2_1_2_1_* | *C2* | *C2* |
| Wavelength (Å) | 0.9793 | 0.9785 | 0.9785 |
| Cell dimensions |  |  |  |
| *a, b, c* (Å) | 50.20, 64.07, 99.58 | 96.40, 81.65, 54.44 | 95.85, 81.78, 54.48 |
| α, β, γ (°) | 90, 90, 90 | 90, 117.19, 90 | 90, 117.03, 90 |
| Resolution (Å) | 49.79-1.80 (1.86-1.80) | 25.94-1.69 (1.75-1.69) | 43.25-1.60 (1.65-1.60) |
| No. of unique reflections | 30348 (2971) | 41356 (3974) | 49708 (4871) |
| Completeness (%) | 99.49 (98.84) | 99.05 (95.52) | 99.49 (96.69) |
| *R*_merge_ | 0.1177 (1.23) | 0.04645 (0.9625) | 0.03369 (1.188) |
| Mean *I*/σ*I* | 16.73 (2.24) | 21.71 (2.02) | 25.65 (1.46) |
| *CC*1/2 | 99.9 (78) | 99.9 (81.8) | 100.0 (77.6) |
| Redundancy | 13.0 (12.8) | 6.8 (6.6) | 6.7 (6.3) |
| Wilson B factors (Å^2^) | 23.10 | 26.76 | 27.43 |
|  |  |  |  |
| **Refinement** |  |  |  |
| Resolution (Å) | 49.79-1.80 | 25.94-1.69 | 43.25-1.60 |
| No. of reflections used | 30338 (2971) | 41338 (3967) | 49638 (4856) |
| *R*_work_ / *R*_free_ | 16.65/20.13 | 16.75/20.45 | 17.26/19.47 |
| No. atoms |  |  |  |
| Protein | 2576 | 2348 | 2389 |
| Ligand/ion | 47 | 45 | 44 |
| Water | 311 | 286 | 271 |
| *B*-factors (Å^2^) |  |  |  |
| Protein | 29.15 | 40.48 | 43.58 |
| Ligand/ion | 34.45 | 50.07 | 52.17 |
| Water | 39.01 | 51.87 | 54.77 |
| R.m.s. deviations |  |  |  |
| Bond lengths (Å) | 0.011 | 0.016 | 0.016 |
| Bond angles (°) | 1.07 | 1.42 | 1.38 |
| Ramachandran plot (%) |  |  |  |
| Favored (%) | 97.50 | 98.33 | 99.00 |
| Allowed (%) | 2.19 | 1.67 | 1.00 |
| Outliers (%) | 0.31 | 0 | 0 |

**Table S4 Preliminary pharmacokinetics (PK) evaluation of compounds 14a and 14b^a^.**

| **Species** | **Compd.** | **Admin.** | **T_1/2_** | **T_max_** | **C_max_** | **AUC_last_** | **AUC_INF_obs_** | **CL** | **MRT_INF_obs_** | **Vss__obs_** | **F** |
| --- | --- | --- | --- | --- | --- | --- | --- | --- | --- | --- | --- |
|  |  |  | **(h)** | **(h)** | **(****ng/mL)** | **(h*ng/mL)** | **(h*ng/mL)** | **(mL/min/kg)** | **(h)** | **(mL/kg)** | **(%)** |
| **mice** | **14a** | i.v. (5 mg/kg) | 18.5 | **-** | **-** | 1220 | 1260 | 66.0 | 2.40 | 9500 | - |
|  |  | i.p. (5 mg/kg) | 7.01 | 0.25 | 1120 | 1260 | 1270 | - | 1.82 | **-** | 101 |
|  |  | p.o. (10 mg/kg) | 1.09 | 0.25 | 124 | 125 | 126 | - | 1.09 | **-** | 5 |
|  | **14b** | i.v. (10 mg/kg) | 3.34 ± 2.38 | - | - | 2896 ± 1021 | 2976 ± 1055 | 62.5 ± 27.4 | 1.88 ± 1.08 | 6419 ± 2698 | - |
|  |  | i.p. (20 mg/kg) | 5.77 ± 0.77 | 0.18 ± 0.12 | 3270 ± 374 | 7298 ± 782 | 7635 ± 600 | - | 5.34 ± 1.81 | **-** | 128 |
|  |  | p.o. (20 mg/kg) | 1.32 ± 0.77 | 0.25 ± 0.00 | 290 ± 189 | 463 ± 225 | 488 ± 219 | - | 2.11 ± 0.87 | **-** | 8 |
| **rats** | **14a** | i.v. (5 mg/kg) | 6.00 ± 0.75 | - | - | 4110 ± 756 | 4130 ± 756 | 20.6 ± 3.4 | 1.08 ± 0.07 | 1300 ± 150 | - |
|  |  | i.p. (5 mg/kg) | 4.14 | 0.25 | 2080 | 4755 | 4765 | - | 1.90 | **-** | 115 |
|  |  | p.o. (10 mg/kg) | 2.52 ± 2.55 | 0.75 ± 0.84 | 416 ± 79 | 1050 ± 276 | 1060 ± 281 | - | 2.06 ± 0.49 | **-** | 13 |
|  | **14b** | i.v. (5 mg/kg) | 5.11 ± 0.10 | - | - | 4220 ± 189 | 4250 ± 188 | 19.6 ± 0.9 | 1.94 ± 0.12 | 2280 ± 184 | - |
|  |  | i.p. (5 mg/kg) | 8.24 ± 1.72 | 0.25 ± 0.00 | 1630 ± 99 | 9300 ± 994 | 10600 ± 557 | - | 10.6 ± 1.8 | **-** | 249 |
|  |  | p.o. (10 mg/kg) | 3.86 ± 1.10 | 0.33 ± 0.14 | 982 ± 58 | 2620 ± 250 | 2680 ± 221 | - | 3.95 ± 1.27 | **-** | 32 |
| **dogs** | **14a** | i.v. (1 mg/kg) | 1.04 | **-** | **-** | 732 | 735 | 24.4 | 0.74 | 1050 | - |
|  |  | s.c. (1 mg/kg) | 3.96 | 0.50 | 116 | 881 | 893 | - | 5.14 | **-** | 120 |
|  |  | p.o. (5 mg/kg) | 1.33 | 0.75 | 154 | 351 | 357 | - | 1.86 | **-** | 10 |
|  | **14b** | i.v. (1 mg/kg) | 1.07 ± 0.29 | - | - | 544 ± 148 | 546 ± 148 | 31.9 ± 7.8 | 0.82 ± 0.11 | 1590 ± 557 | - |
|  |  | s.c. (1 mg/kg) | 3.19 ± 1.57 | 2.08 ± 1.88 | 151 ± 36 | 948 ± 46 | 973 ± 54 | - | 4.69 ± 1.25 | **-** | 178 |
|  |  | p.o. (5 mg/kg) | 2.00 ± 0.85 | 0.58 ± 0.38 | 235 ± 173 | 271 ± 166 | 283 ± 167 | - | 2.19 ± 0.86 | **-** | 10 |

^a^n = 3 animals, data are shown as mean ± SD, if not, data are shown as mean. See PK procedures for detail

**Table S5 Real-time quantitative PCR primers sequences.**

| 1 | *β*-Actin forward | tgagctgcgttttacaccct |
| --- | --- | --- |
| 2 | *β*-Actin reverse | gccttcaccgttccagtttt |
| 3 | IL-1*β* forward | tcgctcagggtcacaagaaa |
| 4 | IL-1*β* reverse | catcagaggcaaggaggaaaa |
| 5 | IL-6 forward | acaagtcggaggcttaattacacat |
| 6 | IL-6 reverse | ttgccattgcacaactcttttc |
| 7 | CCL3 forward | accatgacactctgcaaccaa |
| 8 | CCL3 reverse | ttctcttagtcaggaaaatgacacc |
| 9 | CXCL10 forward | atcatccctgcgagcctatcct |
| 10 | CXCL10 reverse | gaccttttttggctaaacgctttc |
| 11 | TNF*α* forward | aggctgccccgactacgt |
| 12 | TNF*α* reverse | gactttctcctggtatgagatagcaaa |
| 13 | CSF3 forward | catgaagctaatggccctgc |
| 14 | CSF3 reverse | ggcctggatcttcctcacttg |

**Table S6 *In vivo* toxicity study of 14a and 14b in mice.**

| **Study** | Acute toxicity study | |
| --- | --- | --- |
| **Compound** | **14a** | **14b** |
| **Vehicle** | 5% dimethyl sulfoxide, 7.5% Kolliphor HS 15 and 87.5% saline (volume ratio) | 5% dimethyl sulfoxide and 95% hydroxypropyl methyl cellulose solution (0.5% hydroxypropyl methyl cellulose in deionized water in weight ratio) (volume ratio) |
| **Administration** | oral | |
| **Dosage** | 1000 mg/kg/day and 2000 mg/kg/day | |
| **Animals** | 4 mice (2 male and 2 female) per group, 6 groups | |
| **Frequency** | Twice daily | |
| **Results** | No obvious toxicity  1. No mice died after compounds treatment.  2. No abnormalities in body weights, food mean daily consumption and general status were observed in all groups during the period of study.  3. No obvious organs and tissues damage was observed in all groups at the end of observation. | |

**Table S7 Gradient elution systems for separation of 14a and 14b**

| compound | mobile phase A | mobile phase B | gradient | |
| --- | --- | --- | --- | --- |
|  |  |  | time / min | %B |
| **14a** | 0.01 M NaH_2_PO_4_ in water (pH 6.0) | MeOH | 0.00 | 10 |
|  |  |  | 20.00 | 90 |
|  |  |  | 25.00 | 90 |
|  |  |  | 25.01 | 10 |
|  |  |  | 30.00 | 10 |
| **14b** | 0.1% H_3_PO_4_ in water | MeOH | 0.00 | 20 |
|  |  |  | 10.00 | 60 |
|  |  |  | 25.00 | 90 |
|  |  |  | 30.00 | 100 |
|  |  |  | 31.00 | 20 |
|  |  |  | 37.00 | 20 |
